# Global burden and inequalities of drug use disorders from 1990 to 2021 with projections to 2036

**DOI:** 10.7189/jogh.15.04344

**Published:** 2025-12-05

**Authors:** Fuxin Zhang, Zhaowei Xue, Zhen Cao, Lichun Qiao, Xiangyu Fan, Jing Xiao, Zhe Zhang

**Affiliations:** 1 Department of Pharmacy, The Second Affiliated Hospital of Army Medical University, Chongqing, China; 2 Modern Research Center for Traditional Chinese Medicine of Shanxi University, Taiyuan, China; 3 College of Biological and Food Engineering, Changshu Institute of Technology, Changshu, China; 4 School of Public Health, Health Science Center, Xi’an Jiaotong University, Xi’an, China; 5 NHC Key Laboratory of Drug Addiction Medicine, School of Forensic Medicine, Kunming Medical University, Kunming, China

## Abstract

**Background:**

The COVID-19 pandemic further exacerbated the burden of drug use disorders (DUDs), and systematic quantification of inequalities in DUDs remains limited. Thus, a comprehensive evaluation of the global burden and inequalities of DUDs following the COVID-19 pandemic is necessary.

**Methods:**

We used data from the Global Burden of Disease 2021 study to evaluate the global burden of DUDs from 1990 to 2021, stratified by sex, age, country, region, socio-demographic index (SDI), and drug category. The slope index of inequality and the concentration index of inequality are applied to quantify absolute and relative inequalities in both overall and drug-specific burdens across SDI regions. Future trends through 2036 were projected using an autoregressive integrated moving average model and Bayesian age-period-cohort model.

**Results:**

This study revealed that the global burden of DUDs increased greatly from 1990 to 2021, with the highest burden observed among individuals aged 15–49 years and consistently greater in males. High-income North America and the USA bore the highest burden at the regional and national levels, respectively. The analysis by drug category indicated that opioid use disorder represented the predominant contributor to the overall burden of DUDs. Both absolute and relative inequalities in the overall burden of DUDs increased across SDI levels, with marked variations in inequality patterns across drug categories. Inequalities have intensified for opioid and amphetamine use disorders, whereas those related to cannabis use disorders have declined. Both models predicted increasing incidence, deaths, and age-standardised mortality rate accompanied by declining age-standardised prevalence rate, but showed opposite trends for prevalence, disability-adjusted life years (DALYs), age-standardised incidence rate, and age-standardised DALY rate.

**Conclusions:**

Over the past three decades, the burden of DUDs has increased markedly, accompanied by wide disparities. Addressing these challenges requires strengthened surveillance, context-specific interventions, and cross-country learning.

Drug use disorders (DUDs) are marked by the persistent and compulsive use of addictive substances, such as opioids, cannabis, amphetamines, and certain prescription drugs, for non-medical purposes [1,2], leading to dependence and repeated craving for their psychoactive effects. These behaviours result in serious health damage and public health risks, making DUDs an increasingly severe global public health problem [3–9].

The COVID-19 pandemic further exacerbated this burden, which may have been related to disruptions in drug supply chains, reduced access to mental health and addiction treatment services, and increased substance use as a coping mechanism for stress or emotions related to COVID-19 [10–13]. Against this background, timely updates and accurate assessments of global trends and distribution patterns of DUDs are crucial for informing public health strategies and guiding effective interventions.

DUDs not only impose a heavy overall burden but also exhibit pronounced inequalities. However, most previous study mainly focused on assessing the overall burden of DUDs. For instance, Shen et al. utilised data from the Global Burden of Disease (GBD) 2019 study to systematically evaluated DUD-related burden at global, regional, and national levels, revealing substantial variation across regions [3]. Kim et al. analysed trends in DUD-related mortality across 73 countries using World Health Organization mortality data, and examined associations with socioeconomic indicators [10]. Although these studies have provided valuable insights into DUDs, they generally lack a systematic quantitative analysis of health inequalities. In recent years, the slope index of inequality (SII) and the concentration index of inequality (CII) have been increasingly applied in mental health and addiction research, providing important methodological approaches for quantifying inequalities in the burden of DUDs [14–16].

Thus, this study utilises the GBD 2021 data to evaluate the global burden of DUDs from 1990 to 2021, stratified by sex, age, country, region, sociodemographic index (SDI), and drug category. The SII and the CII are applied to quantify absolute and relative inequalities in both overall and drug-specific burdens across SDI regions. This analysis provides a comprehensive evaluation of the burden and inequalities of DUDs following the COVID-19 pandemic, offering new insights to inform resource allocation and the design of fairer public health policies.

This study adhered to JoGH’s Guidelines for Reporting Analyses of Big Data Repositories Open to Public (GRABDROP). Details of adherence are provided in Table S1 in the **Online Supplementary Document**.

## METHODS

### Data collection and case definition

This study utilised publicly available data from the GBD Study 2021, coordinated by the Institute for Health Metrics and Evaluation (IHME). Data were extracted from the GBD Results Tool for the years 1990 to 2021, covering 204 countries and territories, 21 GBD regions, five SDI levels, and global-level estimates [17]. Detailed methods for data collection, processing, modelling, and estimation in the GBD 2021 framework are described before [3].

In GBD 2021, DUDs were defined in accordance with diagnostic criteria from the ICD-10 and DSM-IV-TR, and included opioid use disorders, cocaine use disorders, cannabis use disorders, amphetamine use disorders, and other drug use disorders. These were mapped to ICD-10 codes F11-F11.99 and R78.1 (opioid use disorders); F14-F14.99 and R78.2 (cocaine use disorders); F12-F12.99 (cannabis use disorders); F15-F15.99 (amphetamine use disorders); and F13-F13.99, F16-F19.99, P96.1, and R78.3-R78.9 (other drug use disorders) [18].

Countries and territories were stratified by the SDI, a composite indicator based on average income per capita, educational attainment, and total fertility rate. SDI scores range from 0 to 1, with higher values indicating greater socio-demographic development. Based on their SDI values, countries were classified into five levels: low, low-middle, middle, high-middle, and high SDI. These SDI groups were used to facilitate comparative analyses of disease burden across varying levels of development [19].

### Statistical analysis

We assessed the burden and inequalities of DUDs with projections to 2036 based on GBD 2021 data set. Indicators included incidence, prevalence, deaths, disability-adjusted life years (DALYs), as well as the corresponding age-standardised incidence rate (ASIR), age-standardised prevalence rate (ASPR), age-standardised mortality rate (ASMR), and age-standardised DALY rate (ASDR). Analyses were conducted at the global level and further stratified by sex, age, country, region, SDI, and drug category. To assess health inequalities across SDI levels, associations between SDI and ASRs were evaluated using Spearman correlation. The SII and CII were further used to quantify the absolute and relative inequalities, respectively; these analyses were performed for both overall and drug-specific burdens across SDI regions. Projections for 2022 to 2036 were generated using the autoregressive integrated moving average (ARIMA) and Bayesian age-period-cohort (BAPC) models, which were applied to incidence, prevalence, deaths, DALYs, and their ASRs. The detailed methodology is provided in the **Online Supplementary Document**. All analyses and visualisations were performed in *R* software, version 4.3.3 (R Foundation for Statistical Computing, Vienna, Austria).

All analyses were conducted following the Guidelines for Accurate and Transparent Health Estimates Reporting (GATHER) statement to ensure transparency and reproducibility.

## RESULTS

### Global burden of DUDs

Between 1990 and 2021, the global burden of DUDs rose significantly in terms of incidence, prevalence, deaths, DALYs, ASMR, and ASDR, while ASIR and ASPR showed modest declines (**Table 1**). The number of incident cases rose to 13 609 362.38 in 2021, representing a 35.50% increase compared with 1990, although the ASIR declined by 8.09% (from 184.31 to 169.39 per 100 000 population). Prevalent cases reached 53 115 936.38, with an increase of 34.1%, while the ASPR decreased by 6.39% (from 709.15 to 663.80 per 100 000 population). In terms of mortality, DUDs accounted for approximately 137 277.92 deaths in 2021, more than doubling since 1990, with the ASMR rising by 30.73% (from 1.26 to 1.65 per 100 000 population). Disability-adjusted life years also increased substantially, reaching 15 562 161.53 in 2021, a 74.65% increase compared with 1990, while the ASDR rose by 14.74% (from 166.44 to 190.97 per 100 000 population).

**Table 1:** Global incidence, prevalence, deaths, DALYs, and corresponding ASRs of DUDs from 1990 to 2021

	Absolute numbers (95% UI)			ASRs (per 100 000 population, 95% UI)			
	**1990**	**2021**	**Change (%)**		**1990**	**2021**	**Change (%)**
**Both**							
Incidence	10 043 456.25 (8 541 086.21, 11 526 399.67)	13 609 362.38 (11 625 287.78, 15 667 184.20)	35.50		184.31 (156.91, 211.67)	169.39 (145.14, 195.01)	−8.09
Prevalence	39 620 619.24 (34 071 984.72, 46 420 658.80)	53 115 936.38 (46 999 805.19, 60 949 054.28)	34.06		709.15 (618.81, 824.54)	663.80 (584.52, 766.14)	−6.39
Deaths	61 774.49 (57 328.57, 66 897.82)	137 277.92 (129 268.62, 146 181.36)	122.22		1.26 (1.17, 1.37)	1.65 (1.55, 1.75)	30.73
DALYs	8 910 603.44 (7 055 603.29, 1 063 0912.05)	15 562 161.53 (12 752 221.99, 18 119 263.56)	74.65		166.44 (132.55, 198.40)	190.97 (156.11, 222.79)	14.74
**Male**							
Incidence	5 492 790.83 (4 711 061.73, 6 275 603.45)	7 441 387.15 (6 451 385.82, 8 533 369.25)	35.48		197.41 (169.42, 224.95)	183.99 (159.71, 211.51)	−6.80
Prevalence	23 458 847.49 (20 079 478.04, 27 756 438.22)	31 497 541.37 (27 623 062.04, 36 535 985.81)	34.27		829.31 (718.57, 971.49)	781.25 (683.39, 909.07)	−5.80
Deaths	42 438.92 (38 433.25, 46 768.42)	97 823.41 (92 789.92, 103 995.01)	130.50		1.74 (1.58, 1.92)	2.37 (2.24, 2.51)	36.21
DALYs	5 353 145.49 (4 327 189.48, 6 328 753.66)	9 656 105.18 (8 185 306.53, 11 042 243.67)	80.38		198.52 (161.30, 234.34)	235.88 (199.50, 270.25)	18.82
**Female**							
Incidence	4 550 665.42 (3 842 927.34, 5 292 605.98)	6 167 975.23 (5 217 454.56, 7 167 941.94)	42.13		170.75 (143.40, 198.12)	154.12 (130.49, 179.18)	−9.74
Prevalence	16 161 771.75 (14 054 022.51, 18 836 159.86)	21 618 395.01 (19 253 944.32, 24 604 457.14)	33.76		586.02 (515.68, 678.26)	542.90 (480.87, 620.72)	−7.36
Deaths	19 335.57 (17 088.03, 21 882.68)	39 454.52 (36 090.89, 43 023.37)	104.05		0.79 (0.70, 0.89)	0.93 (0.85, 1.01)	17.72
DALYs	3 557 457.95 (2 719 992.05, 4 315 647.52)	5 906 056.35 (4 616 817.21, 7 109 842.66)	66.02		133.73 (102.80, 161.92)	145.25 (113.42, 175.63)	8.61

ASRs – age-standardised rates, DALYs – disability-adjusted life years, DUDs – drug use disorders, UI – uncertainty interval

Age- and sex-specific analyses demonstrated marked heterogeneity in the burden of DUDs (Figure S1 in the **Online Supplementary Document**). In 2021, the number of incident cases was concentrated among individuals aged 15–54 years, with the highest rate observed in the 15–19 age group. Prevalence peaked at ages 20–24, and prevalent cases were predominantly observed among individuals aged 15–39 years. Across ages 10–49, males consistently exhibited a higher burden than females in terms of incidence and prevalence. The number of deaths was greatest among individuals aged 20–64 years, with mortality rate increasing between ages 15–39, declining at 40–74, and rising again in those ≥75 years. Males had higher mortality across nearly all age groups. Disability-adjusted life years were concentrated in the 20–49-year age range, with rates peaking at ages 25–29 and remaining consistently higher in males than in females across ages 10–69. Overall, the DUDs burden in 2021 was primarily concentrated among young and middle-aged adults (15–49 years), with males bearing a disproportionate share across all indicators.

### Regional and national variation in DUD-related burden

At the GBD regional level, the burden of DUDs showed marked variation across the 21 global regions from 1990 to 2021 (Table S1–4 in the **Online Supplementary Document**). High-income North America consistently ranked highest across multiple indicators. It recorded the greatest number of prevalence, deaths, and DALYs in 2021, representing increases of 121.45%, 1115.62%, and 531.47%, and the highest ASRs across all indicators. Oceania recorded the lowest absolute numbers of incident cases, prevalent cases, deaths, and DALYs, while Western sub-Saharan Africa had the lowest ASRs. East Asia was the only region that exhibited consistent declines across all indicators, each showing a reduction of over 15% from 1990 to 2021.

In 2021, the USA, China, and India ranked among the top countries in terms of the burden of DUDs across multiple indicators (Table S5–8 in the **Online Supplementary Document**). The USA ranked first in prevalent cases (12 146 953.91 cases; 95% uncertainty interval (UI) = 11 024 582.17, 13 461 043.90), deaths (70 893.11 deaths; 95% UI = 64 047.09, 78 963.69), DALYs (6 484 690.40 DALYs; 95% UI = 5 471 717.29, 7 481 321.26), and all ASRs (ASIR = 531.19, ASPR = 3821.43, ASMR = 19.52, ASDR = 1944.08 per 100 000). China reported the highest number of incident cases (2 451 314.00 cases; 95% UI = 2 046 472.04, 2 907 370.53).

### Health inequality in the burden of DUDs across SDI levels

In 2021, countries in the highest SDI quintile recorded the greatest burden of DUDs across nearly all indicators (Table S1–4 in the **Online Supplementary Document**). It ranked first in the number of prevalent cases (19 257 655.43 cases; 95% UI = 17 598 069.60, 21 398 277.69), deaths (87 936.65 deaths; 95% UI = 80 866.65, 96 448.75), and DALYs (8 333 571.68 DALYs; 95% UI = 7 048 826.98, 9 607 578.71), and also reported the highest ASRs, including an ASIR of 350.90 per 100 000 population (95% UI = 307.36, 400.20), ASPR of 1897.69 (95% UI = 1 710.93, 2137.33), ASMR of 7.07 (95% UI = 6.54, 7.71), and ASDR of 752.61 (95% UI = 630.61, 872.87).

**Figure 1:**
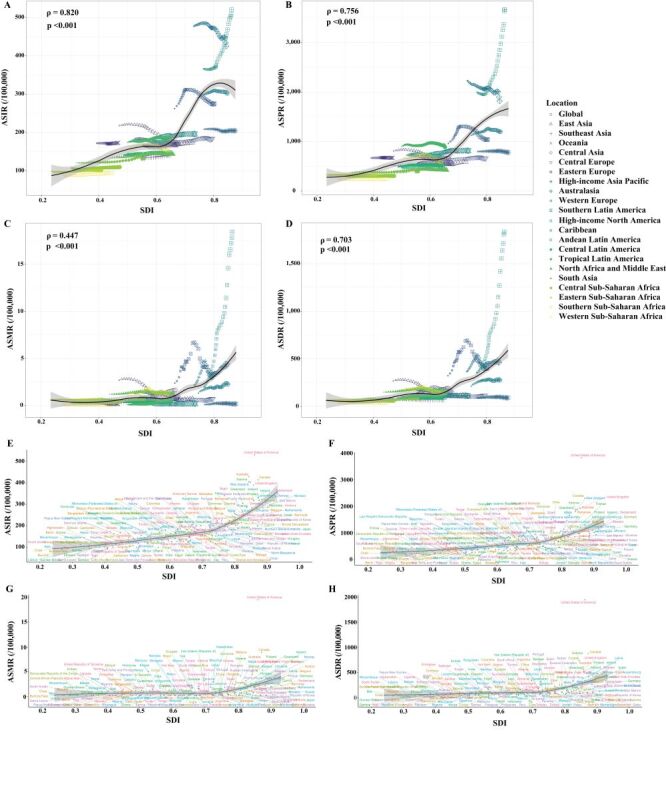
ASRs of DUDs globally and across 21 GBD regions by SDI from 1990 to 2021, and across 204 countries and territories in 2021. **Panels A–D.** ASIR, ASPR, ASMR, and ASDR at the global and regional levels, respectively. **Panels E–H.** ASIR, ASPR, ASMR and ASDR for 204 countries and territories, respectively. ASIR – age-standardised incidence rate, ASPR – age-standardised prevalence rate, ASMR – age-standardised mortality rate, ASDR – age-standardised DALY rate, DUDs – drug use disorders, GBD – global burden of disease, SDI – sociodemographic index.

Spearman correlation analysis demonstrated a significant positive association between SDI and ASRs of DUDs at the global, regional, and national levels from 1990 to 2021 (**Figure 1**). While ASRs remained relatively low and stable in low-SDI regions, high-SDI regions showed marked heterogeneity. Notably, High-income North America recorded ASRs substantially higher than expected for its SDI level, whereas the High-income Asia Pacific region showed much lower rates (**Figure 1**, Panels A–D). At the national level, ASRs in most countries were close to the values expected based on their SDI. However, the USA exhibited substantially higher-than-expected ASRs across all indicators in 2021 (**Figure 1**, Panels E–H).

**Figure 2:**
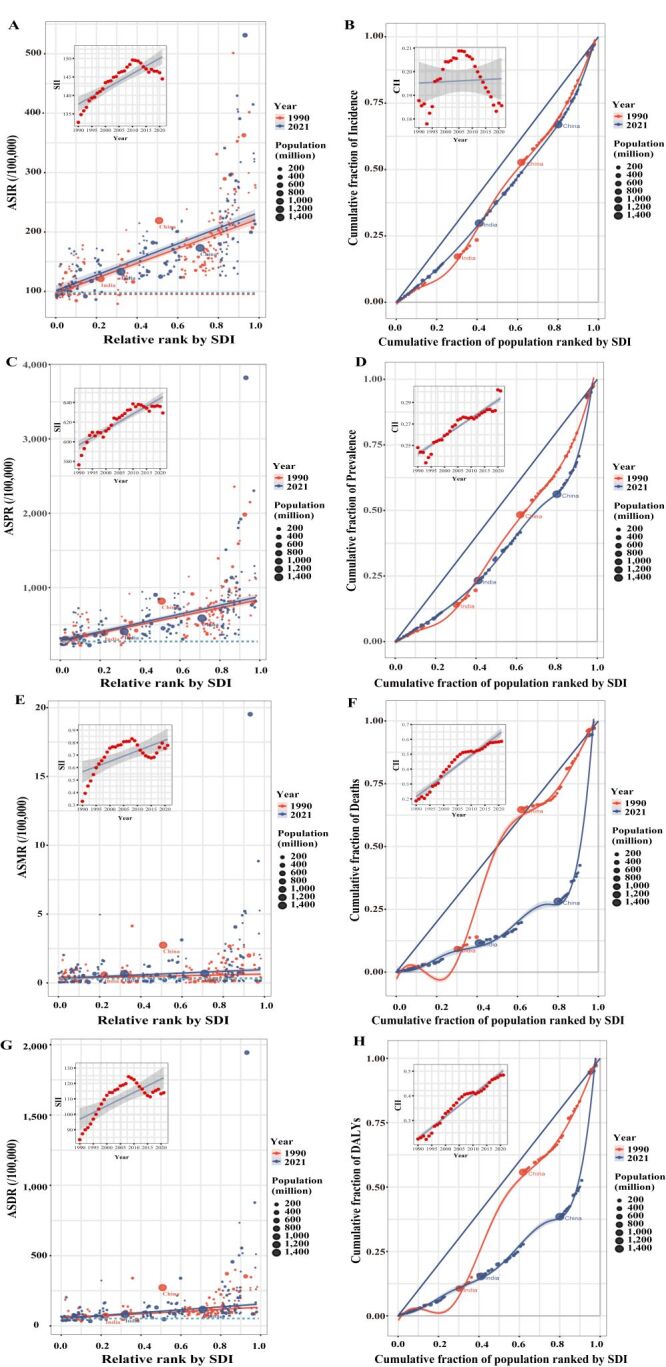
Absolute and relative inequalities in the burden of DUDs by SDI from 1990 to 2021. **Panels A** and **B.** Incidence. **Panels C** and **D.** Prevalence. **Panels E** and **F.** Deaths. **Panels G** and **H.** DALYs. In each pair, the left panel displays the SII, while the right panel presents the CII. ASIR – age-standardised incidence rate, ASPR – age-standardised prevalence rate, ASMR – age-standardised mortality rate, ASDR – age-standardised DALY rate, CII – concentration index of inequality, DALY – disability-adjusted life year, DUDs – drug use disorders, SDI – sociodemographic index, SII – slope index of inequality.

Inequality in the burden of DUDs across SDI levels was assessed using both absolute (SII) and relative (CII) measures (**Figure 2**, **Table 2**). The slope index of inequality quantified the absolute difference in burden between the highest and lowest SDI quintiles. From 1990 to 2021, SII values increased across all ASRs, indicating widening absolute difference (**Figure 2**, Panels A, C, E, and G). Relative inequality, measured by the CII, remained largely unchanged for incidence but increased for prevalence (0.25–0.30), deaths (0.19–0.59), and DALYs (0.22–0.48) (**Figure 2**, Panels B, D, F, and H).

**Table 2:** SII and CII for DUDs by drug categories

	Incidence (95% CI)				Prevalence (95% CI)				Death (95% CI)				DALY (95% CI)			
	**SII**		**CII**		**SII**		**CII**		**SII**		**CII**		**SII**		**CII**	
	**1990**	**2021**	**1990**	**2021**	**1990**	**2021**	**1990**	**2021**	**1990**	**2021**	**1990**	**2021**	**1990**	**2021**	**1990**	**2021**
Total	132.74 (116.75, 148.72)	144.45 (128.37, 160.53)	0.18 (0.13, 0.24)	0.18 (0.10, 0.27)	576.41 (499.74, 653.08)	629.32 (545.00, 713.64)	0.25 (0.19, 0.31)	0.30 (0.21, 0.39)	0.33 (0.12, 0.53)	0.78 (0.48, 1.07)	0.19 (0.14, 0.23)	0.59 (0.48, 0.69)	83.53 (68.46, 98.60)	113.94 (91.85, 136.04)	0.22 (0.17, 0.28)	0.48 (0.38, 0.60)
Opioid	9.74 (6.52, 12.96)	13.65 (10.07, 17.23)	0.13 (0.08, 0.18)	0.24 (0.13, 0.34)	88.37 (65.23, 111.51)	104.11 (80.38, 127.84)	0.22 (0.16, 0.28)	0.44 (0.32, 0.56)	0.16 (0.00, 0.32)	0.44 (0.22, 0.66)	0.20 (0.16, 0.25)	0.61 (0.50, 0.72)	44.15 (30.90, 57.40)	59.94 (43.22, 76.66)	0.22 (0.17, 0.27)	0.51 (0.39, 0.63)
Amphetamine	15.27 (12.22, 18.31)	16.15 (12.76, 19.53)	0.17 (0.13, 0.21)	0.32 (0.29, 0.36)	105.90 (82.10, 129.71)	111.31 (85.08, 137.54)	0.17 (0.13, 0.21)	0.34 (0.30, 0.37)	0.02 (0.01, 0.02)	0.07 (0.06, 0.08)	0.08 (0.07, 0.10)	0.62 (0.56, 0.68)	15.01 (11.72, 18.31)	19.48 (15.33, 23.63)	0.16 (0.12, 0.19)	0.42 (0.38, 0.45)
Cannabis	44.57 (34.91, 54.23)	39.21 (29.68, 48.74)	0.18 (0.09, 0.27)	0.09 (−0.00, 0.18)	279.82 (215.82, 343.82)	242.21 (179.66, 304.75)	0.24 (0.14, 0.34)	0.14 (0.04, 0.23)	no data	no data	no data	no data	8.16 (6.31, 10.01)	7.03 (5.22, 8.85)	0.24 (0.14, 0.34)	0.14 (0.04, 0.23)
Cocaine	5.82 (4.58, 7.06)	6.14 (4.84, 7.43)	0.54 (0.45, 0.63)	0.45 (0.33, 0.58)	76.68 (59.07, 94.29)	84.53 (65.78, 103.29)	0.65 (0.56, 0.73)	0.57 (0.44, 0.69)	0.04 (0.02, 0.06)	0.05 (0.02, 0.07)	0.28 (0.18, 0.38)	0.53 (0.38, 0.67)	13.34 (10.10, 16.59)	14.69 (10.96, 18.41)	0.53 (0.43, 0.63)	0.54 (0.40, 0.67)
Other drugs	54.06 (47.09, 61.03)	58.11 (51.00, 65.23)	0.20 (0.14, 0.25)	0.19 (0.12, 0.27)	15.81 (13.36, 18.27)	18.14 (15.61, 20.66)	0.30 (0.23, 0.36)	0.36 (0.29, 0.44)	0.09 (0.06, 0.11)	0.15 (0.11, 0.19)	0.14 (0.11, 0.17)	0.48 (0.40, 0.55)	6.45 (5.06, 7.84)	9.44 (7.23, 11.65)	0.16 (0.12, 0.20)	0.46 (0.39, 0.53)

CI – confidence interval, CII – concentration index, DUDs – drug use disorders, SII – slope index of inequality

### Burden and inequalities of DUDs by drug categories

When stratified by drug categories, distinct patterns were observed (Table S9–13 in the **Online Supplementary Document**). Opioid use disorder emerged as the predominant type of DUDs, with deaths, DALYs, ASMR, and ASDR consistently higher than those of other substances. All indicators for opioid use disorder increased substantially over time. For amphetamine use disorder, incidence, prevalence, DALYs and related ASRs declined, whereas deaths and ASMR increased. Cannabis use disorder and other drug use disorders showed increases in absolute numbers but decreases in ASRs. For cocaine use disorder, all indicators except ASIR and ASPR increased.

Consistent with the overall inequality analysis of DUDs, the burden of different drug categories was also concentrated in high-SDI regions (**Table 2**). However, the patterns of inequality across different drug categories varied substantially. Opioid and amphetamine use disorders showed increases in both SII and CII across all indicators in 2021 compared with 1990, whereas cannabis use disorder showed decreases across all indicator except for deaths. For cocaine and other drug use disorders, SII increased across all indicators, and CII for deaths and DALYs also increased.

### Projections of DUD-related burden 2022–2036

**Figure 3:**
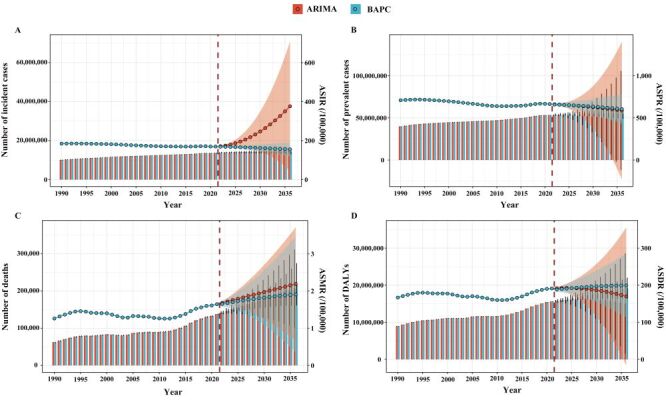
Projected global burden of DUDs from 2022 to 2036. **Panel A.** Number of incident cases and ASIR. **Panel B.** Number of prevalent cases and ASPR. **Panel C.** Number of deaths and ASMR. **Panel D.** Number of DALYs and ASDR. Bar charts represent the number of cases or DALYs, and line charts represent the corresponding ASRs. ASIR – age-standardised incidence rate, ASPR – age-standardised prevalence rate, ASMR – age-standardised mortality rate, ASDR – age-standardised DALY rate, ASRs – age-standardised rates, DALYs – disability-adjusted life-years, DUDs – drug use disorders.

Trends in DUD-related burden 2022–2036 were projected using the ARIMA and BAPC models for all indicators. Both models showed increasing trends in incidence, deaths, and ASMR, but a declining trend for ASPR. However, discrepancies were observed for prevalence, DALYs, ASIR, and ASDR: the ARIMA model projected an increase in ASIR but a decline in prevalence and ASDR, while DALYs were predicted to rise initially and then decrease thereafter. In contrast, the BAPC model projected a decrease in ASIR but increases in prevalence, DALYs, and ASDR (**Figure 3****,** Table S14–15 in the **Online Supplementary Document**).

## DISCUSSION

This study revealed that the global burden of DUDs increased greatly from 1990 to 2021, with the highest burden observed among individuals aged 15–49 years and consistently greater in males. High-income North America and the USA bore the highest burden at the regional and national levels, respectively. The analysis by drug category indicated that opioid use disorder represented the predominant contributor to the overall burden of DUDs. Both absolute and relative inequalities in the overall burden of DUDs increased across SDI levels, with marked variations in inequality patterns across drug categories. Inequalities have intensified for opioid and amphetamine use disorders, whereas those related to cannabis use disorders have declined. Both models predicted increasing incidence, deaths, and ASMR accompanied by declining ASRP, but showed opposite trends for prevalence, DALYs, ASIR, and ASDR.

Compared with the findings of Shen et al. based on GBD 2019 data [1], our study showed a larger decline in ASIR (−8.09 *vs*. −0.73%) and a reversal in ASPR (−6.39 *vs*. 3.17%), likely reflecting underreporting during the pandemic and the impact of competing mortality from COVID-19 [20–23]. Both studies found increases in ASMR and ASDR, though our estimates were smaller (30.73 *vs*. 45.45% for ASMR; 14.74 *vs*. 19.66% for ASDR), which may also relate to pandemic-related competing risks as well as differences in study periods and modelling methods.

Although our study and that of Kim et al. differ in data sources (GBD 2021 *vs*. WHO Mortality Database) [10], both consistently identified a sustained rise in DUD-related mortality since 1990 and projected a continued increase over the forecast period based on the BAPC model. The two studies demonstrated strong concordance in the trajectories of ASMR, underscoring the robustness of these temporal patterns across different data sets.

The burden of DUDs also shows marked variation by sex and age. Globally, DUDs are concentrated in individuals aged 15–49 years, with males consistently bearing a higher burden than females across most age groups. The heavier burden in males may be related to greater sensitivity of the reward system [24], a higher tendency to cope with stress through substance use [25,26], and sociocultural expectations for strength and independence that impose gendered pressures [27].

At the regional level, DUD-related ASRs were strongly correlated with the SDI, with the highest burden observed in high-SDI regions, particularly in high-income North America. This disproportionately high burden may be linked to several factors [6,9,28–33]. Advanced health care systems and pharmaceutical supply chains, as well as insufficient primary prevention, may increase access to addictive prescription medications, which could contribute to over-prescription and misuse, as seen in the USA opioid crisis [9,28,29]; Although the USA has implemented a series of measures, the issue of drug abuse and overdose deaths has not been effectively mitigated due to excessive prescribing in the early stages and delays in policy implementation [30]. High-SDI regions have more fully undergone a three-stage progression of substance use, with each phase potentially compounding the overall burden [31]; the presence of well-established illicit drug markets might exacerbate the risks of addiction and overdose [6]; the higher prevalence of mental illness and comorbidities in these settings may promote drug use as a form of self-medication [32]. More robust health surveillance and reporting systems likely contribute to more complete burden estimation [33].

In contrast, East Asia showed declines in all indicators. Given that China accounts for the dominant share of the regional population, these declines may be mainly drive by China. The sustained reduction in China's DUD-related burden may be partly explained by comprehensive governance measures, including strengthened anti-drug legislation, expanded methadone maintenance treatment programmes, community-based rehabilitation initiatives, and psychosocial interventions [34,35]. Moreover, measures such as cross-border drug control, digital surveillance of drug-related content, targeted interventions among high-risk populations, and addiction monitoring systems comprise multi-layered prevention strategy [36,37]. Nonetheless, possible data limitations, underreporting, and cultural influences on case ascertainment may also contribute to the observed declines.

Further inequality analysis highlighted substantial absolute and relative difference in the burden of DUDs across SDI levels. The SII, which reflects the absolute difference between the highest- and lowest-SDI regions, increased for ASIR, ASPR, ASMR, and ASDR from 1990 to 2021, indicating that the absolute gaps between high- and low-SDI regions in these ASRs widened over time. Similarly, the CII, which measures the relative difference of burden, showed that deaths and DALYs became increasingly concentrated in high-SDI regions. In simple terms, this means that not only has the absolute difference in burden between regions at the highest and lowest SDI levels grown larger, but the share of the most severe outcomes, particularly deaths and DALYs, has increasingly shifted toward high-SDI regions, thereby intensifying global health inequalities. Consistent with the overall inequality analysis of DUDs, the burden of different drug categories was more concentrated in high-SDI countries, although the specific patterns varied. For opioid and amphetamine use disorders, the absolute gap between high- and low-income regions widened further, and their relative concentration in high-income regions also increased. The burden of cannabis use disorder remained higher in high-income countries, but both the absolute gap and the degree of concentration declined over time. Cocaine and other drug use disorders showed a more complex pattern of inequality, with absolute difference increasing but relative concentration displaying mixed trends.

This study applied a dual-model strategy combining ARIMA and BAPC to enhance the robustness of projections. As a classical time-series model, ARIMA can effectively capture recent trends; however, it relies heavily on historical continuity and is sensitive to abrupt policy or market shifts. In contrast, BAPC decomposes age, period, and cohort effects, placing greater emphasis on long-term demographic and structural shifts. Our results demonstrated that both models project an upward trend for incidence, deaths, and ASMR, and a decline for ASPR. However, discrepancies were observed in prevalence, DALYs, ASIR, and ASDR. These discrepancies might result from the models' different methodological presumptions, which emphasises the value of using a variety of forecasting techniques to make sure that both short-term trends and long-term structural factors are taken into account in order to provide more thorough evidence for public health policy.

The findings of this study could carry several implications for public health policy and governance. First, the global monitoring system for DUDs remains fragmented, with gaps in coverage and timeliness, particularly in low-SDI settings. Future strategies should integrate DUD prevention and control into primary health care, and establish routine surveillance within national health information systems [38–43]. Second, intervention priorities should be tailored to contextual needs. In high-income settings such as North America, the high burden of DUDs highlights the importance of stricter regulation of prescription opioids. In resource-limited environments, priority should be given to low-cost, wide-coverage early interventions, including health education, community-based prevention, and sentinel surveillance [44,45], together with localised harm reduction measures such as needle and syringe programmes, opioid substitution therapy, provision of naloxone as an emergency anti-dote to opiate overdose [46]. Moreover, international experience demonstrates the importance of coordination between criminal justice and public health systems. Programmes combining judicial supervision with medical referral, drug courts, medication-assisted treatment in correctional settings, and continuity-of-care arrangements before release have proven effective in reducing recidivism, enhancing treatment uptake, and improving public safety [47–50]. Finally, the comprehensive approaches adopted in East Asia also provide valuable lessons for other regions.

Although GBD 2021 captures recent trends such as the impact of COVID-19, thereby offering a stronger foundation for evaluating the burden of DUDs, there are still deficiencies in the available data. In low-income countries, particularly in conflict and unstable environments, there is a lack of effective administrative capacity and data collection systems, and severe data deficiency is a widespread issue [51,52]. As reported by the National Survey on Drug Use and Health, high-risk groups such as incarcerated populations, displaced persons, and the homeless are often excluded [53]. Moreover, because deaths caused by cannabis use disorders are not estimated in the GBD 2021, the total number of DUD-related deaths may be underestimated. Although the GBD database uses the CODEm and DisMod-MR 2.1 models to address the missing data [3], estimates for situations described above remain less reliable. Beyond these data limitations, the GBD 2021 deﬁnes DUDs based on ICD-10 or DSM-IV-TR criteria, whereas DSM-5 consolidates drug abuse and drug dependence into a single diagnostic category of DUDs, which may result in higher reported estimates of the overall burden of DUDs. The use of different diagnostic standards therefore limits direct comparability across countries. Finally, we did not conduct an in-depth analysis of the driving factors behind the burden and inequalities of DUDs, such as population effects (*e.g*. aging, migration) and health system effects (*e.g*. health care policies, resource distribution). These factors should be explored further in future research.

## CONCLUSIONS

Over the past three decades, the burden of DUDs has risen greatly, with pronounced disparities across sex, age, countries, regions, SDI levels, and drug categories. Addressing this challenge requires strengthening monitoring systems, implementing context-specific interventions, and drawing on international experiences to promote more equitable and effective governance. Future research should further explore the driving factors of DUD-related burden and inequalities, and conduct subnational analyses to better capture within-country disparities.

## Additional material


Online Supplementary Document
