# Supplementary material for: Global burden and inequalities of drug use disorders from 1990 to 2021 with projections to 2036
Source: J Glob Health. 2025 Dec 5;15:04344. doi: 10.7189/jogh.15.04344 (PMC12679053; doi:10.7189/jogh.15.04344)
Supplement: Online Supplementary Document [file jogh-15-04344-s001.pdf]

Supplement to: Zhang F, Xue Z, Cao Z, Qiao L, Fan X, Xiao J, Zhang Z. Global burden and inequalities of drug use disorders from 1990 to 2021 with projections to 2036. J Glob Health. 2025;15:04344.

METHODS

Data collection and case definition

This study utilized publicly available data from the GBD Study 2021, coordinated by the Institute for Health Metrics and Evaluation (IHME). Data were extracted from the GBD Results Tool (https://vizhub.healthdata.org/gbd-results/) for the years 1990 to 2021, covering 204 countries and territories, 21 GBD regions, 5 SDI levels, and global-level estimates. GBD 2021 synthesizes multiple input sources, including vital registration systems, household surveys, hospital and claims data, and epidemiological studies, which are adjusted and modeled within the GBD framework. For missing or uncertain data, GBD 2021 addresses this through established modeling approaches, including DisMod-MR 2.1 for non-fatal estimates and CODEm for mortality, incorporating relevant covariates to improve estimates. Detailed methods for data collection, processing, modelling, and estimation in the GBD 2021 framework are described before.

In GBD 2021, DUDs were defined in accordance with diagnostic criteria from the ICD-10 and DSM-IV-TR, and included opioid use disorders, cocaine use disorders, cannabis use disorders, amphetamine use disorders, and other drug use disorders. These were mapped to ICD-10 codes F11-F11.99 and R78.1 (opioid use disorders); F14-F14.99 and R78.2 (cocaine use disorders); F12-F12.99 (cannabis use disorders); F15-F15.99 (amphetamine use disorders); and F13-F13.99, F16-F19.99, P96.1, and R78.3-R78.9 (other drug use disorders).

Countries and territories were stratified by the SDI, a composite indicator based on average income per capita, educational attainment, and total fertility rate. SDI scores range from 0 to 1, with higher values indicating greater socio-demographic development. Based on their SDI values, countries were classified into five levels: low, low-middle, middle, high-middle, and high SDI. These SDI groups were used to facilitate comparative analyses of disease burden across varying levels of development.

Statistical analysis

We assessed the burden and inequalities of DUDs with projections to 2036 based on GBD 2021 dataset. Indicators included incidence, prevalence, deaths, DALYs, as well as the corresponding age-standardized incidence rate (ASIR), age-standardized prevalence rate (ASPR), age-standardized mortality rate (ASMR), and age-standardized DALY rate (ASDR).

The calculation formula for age-standardized rates (ASRs) is:

$$\text{ASRs} = \frac{\sum_{i=1}^{A} \alpha_i \omega_i}{\sum_{i=1}^{A} \omega_i} \times 100{,}000$$

Where α_i represents the crude rate for the i-th age group, ω_i represents the corresponding weight (population) of that age group in the standard population, A represents the total number of age groups.

Analyses were conducted at the global level and further stratified by sex, age, country, region, SDI, and drug category, including opioid, amphetamine, cannabis, cocaine, and other drug use disorders, to capture substance-specific patterns.

To assess health inequalities across SDI levels, associations between SDI and ASRs (including ASIR, ASPR, ASMR, and ASDR) were evaluated using Spearman correlation. The SII and CII were further used to quantify the absolute and relative inequalities, respectively. These analyses were performed for both overall and drug-specific burdens across SDI regions. The SII was calculated by regressing the country-level ASRs of DUDs against their relative SDI positions, determined by the midpoint of the cumulative population distribution ranked by SDI. The CII was calculated based on the Lorenz concentration curve fitted to the cumulative population and disease burden distributions (incidence, prevalence, deaths, and DALYs), with the CII value calculated as twice the area between the concentration curve and the line of equality.

Projections for 2022 to 2036 were generated using the autoregressive integrated moving average (ARIMA) and Bayesian age-period-cohort (BAPC) models. The ARIMA model integrates the Autoregressive (AR) and Moving Average (MA) components with differencing (I) to address non-stationarity. It assumes that data series are time-dependent random variables, with their autocorrelation captured by the ARIMA model. This allows for predicting future values based on historical data. The specific steps are as follows: 1) parameter selection: We first performed a stationarity test on the original time series using the Augmented Dickey–Fuller (ADF) unit root test and determined the differencing order (d) accordingly. The differenced series was then subjected to a white noise test to confirm its suitability for further modeling. Based on the autocorrelation function (ACF) and partial autocorrelation function (PACF) plots, we identified candidate values for the autoregressive (p) and

moving average (q) terms. The optimal combination of (p, d, q) was selected using the minimization of the Akaike Information Criterion (AIC) and Bayesian Information Criterion (BIC); 2) Model specification and validation: To validate the final model, we conducted the Box–Ljung test to assess residual independence, the Shapiro-Wilk test to examine residual normality, and an additional ADF test on the differenced series to verify stationarity. These diagnostic checks ensured that the ARIMA model satisfied the key assumptions of stationarity, residual independence, and approximate normality.

We also utilized the BAPC model to forecast the future burden of DUDs, leveraging its capability to handle complex, high-dimensional, and sparse data common in large-scale epidemiological studies like GBD 2021. The BAPC model extends the traditional Generalized Linear Model (GLM) framework within a Bayesian context, dynamically integrating age, period, and cohort effects. These effects are assumed to evolve continuously over time and are smoothed using a second-order random walk, enhancing the accuracy of posterior estimates. A key strength of the BAPC model is its use of the Integrated Nested Laplace Approximation (INLA) method to approximate the marginal posterior distribution, effectively addressing issues such as mixing and convergence often encountered with Markov Chain Monte Carlo techniques while maintaining computational efficiency. The model's flexibility and robustness in handling time series data make it particularly suitable for long-term disease burden predictions. Its comprehensive coverage and ability to capture temporal trends have led to its widespread validation and application in epidemiological research, especially in studies involving age-structured population data and complex cohort effects. This approach enables nuanced predictions of future disease burdens while considering the intricate interactions of age, period, and cohort effects.

All analyses and visualizations were performed in R software, version 4.3.3 (R Foundation for Statistical Computing, Vienna, Austria).

All analyses were conducted following the Guidelines for Accurate and Transparent Health Estimates Reporting (GATHER) statement to ensure transparency and reproducibility.

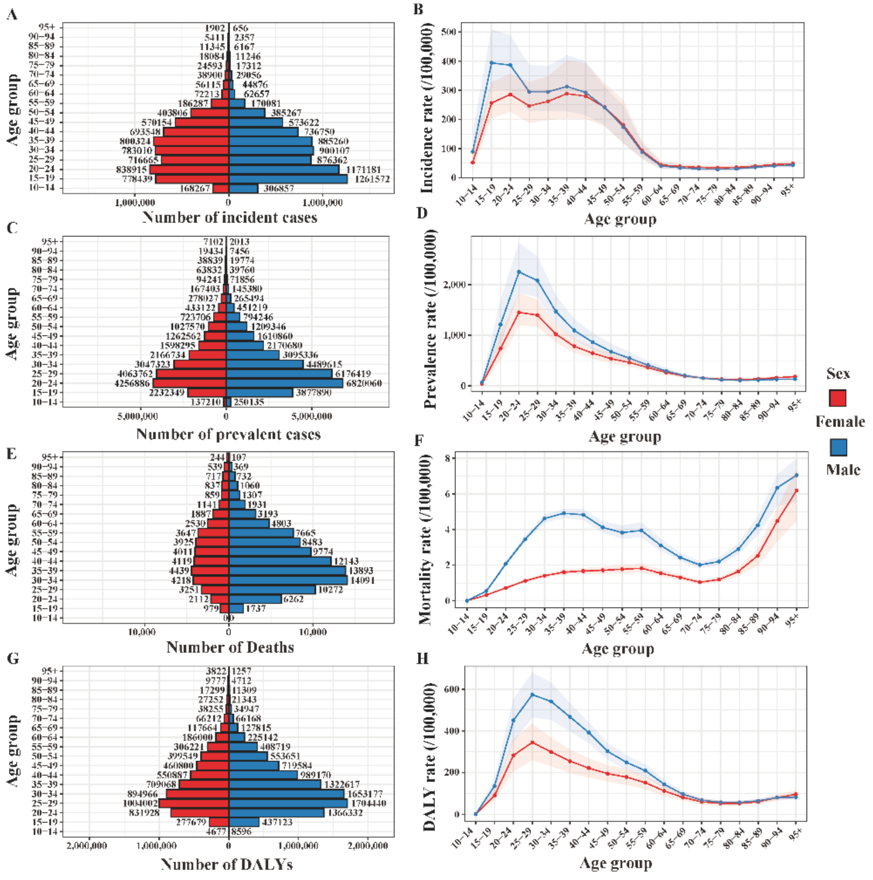

Figure S1. The number of cases and rate per 100,000 population for incidence, prevalence, deaths, and DALYs by age and sex in 2021. **Panel A:** Number of incident cases; **Panel B:** Incidence rate; **Panel C.** Number of prevalent cases; **Panel D:** Prevalence rate; **Panel E:** Number of deaths; **Panel F:** Mortality rate; **Panel G:** Number of DALYs; **Panel H:** DALY rate. DALYs-disability-adjusted life years.

Table S1. Outline of JoGH guideline items

JoGH guideline item	Author's Response
1. Please list all papers published by each co-author in previous 3 years that were based on secondary analysis of a big data repository	[1] **Qiao L**, Li M, Wen X, Deng F, Hou X, Zou Q, Liu H, **Fan X**, Han J. Epidemiological trends in the burden of chronic kidney disease due to lead exposure in China from 1990 to 2021 compared to global, and projections until 2050. Int J Environ Health Res. 2025 Feb 5:1-12. https://doi.org/10.1080/09603123.2025.2461700<br>[2] Luo Y, **Qiao L**, Li M, Wen X, Zhang W, Li X. Global, regional, national epidemiology and trends of Parkinson's disease from 1990 to 2021: findings from the Global Burden of Disease Study 2021. Front Aging Neurosci. 2025 , 16:1498756. https://doi.org/10.3389/fnagi.2024.1498756<br>[3] Wen X, **Qiao L**, Deng F, Zhou J, Li M, Wang L, Deng H, Amhare AF, Han J, Guo Y. Global, regional, and national burden of ischemic heart disease attributable to lead exposure, 1990-2021: decomposition, frontier, and projection analysis. Front Public Health. 2025 , 13:1567747. https://doi.org/10.3389/fpubh.2025.1567747<br>[4] Shi S, **Qiao L**, **Fan X**, Liu Y, Han J, Guo Y. Analysis of brain tumor disease burden in China from 1990 to 2021 and its prediction. Chinese Journal of Cancer Prevention and Treatment. 2025, 32(15):901-910+919. https://doi.org/10.16073/j.cnki.cjcpt.2025.15.01<br>[5] Shi S, Dong X, Zhang Y, **Fan X**, **Qiao L**, Wang L, Han J. Trends and predictions of otitis media disease burden in China from 1990 to 2021. Chinese Preventive Medicine. 2025, 26(06):735-741. https://doi.org/10.16506/j.1009-6639.2025.06.015

The other co-authors have not published such studies during this period.

2. Please explain the key elements of your study design and the use of the available datasets that make your study an original scientific contribution	Key elements of study design:<br>This study conducted a comprehensive secondary analysis of the GBD 2021 dataset for DUDs. The key elements of the design are as follows:<br>(i) systematic assessment of the DUD-related burden (incidence, prevalence, deaths, DALYs, and their corresponding ASRs), stratified by region, sex, age, SDI level, and drug category;<br>(ii) quantification of health inequalities using Spearman correlation, absolute inequality analysis (SII), and relative inequality analysis (CII), applied to both overall burden and drug categories;<br>(iii) application of ARIMA and BAPC models to predict trends from 2022 to 2036.<br><br>Original scientific contribution through use of datasets:<br>Our study adds originality by combining burden estimation, inequality assessment, and predictive model within one framework using the GBD 2021 dataset. We applied SII and CII to quantify absolute and relative inequalities at both overall and drug-specific levels, and used ARIMA and BAPC models to strengthen the robustness of forecasts. This analysis provides a comprehensive evaluation of DUDs following the COVID-19 pandemic, offering new insights to inform resource allocation and fairer public health policies.

3. Please list all publications that addressed similar research questions in the same dataset and indicate where you cited them in your paper	**Based on GBD 2021 dataset**<br>[1] Zhang T, Sun L, Yin X, et al. Burden of drug use disorders in the United States from 1990 to 2021 and its projection until 2035: results from the GBD study. BMC Public Health. 2024, 24(1): 1639. (This study has been cited in the Introduction)<br>[2] Zhang S, Qi X, Wang Y et al. Global burden of drug use disorders by region and country, 1990–2021. Front. Public Health 2024, 12:1470809. (This study has not been cited in our study)<br>[3] Fang M, Zhang Q, Peng J, et al. Global, regional, and national burden of opioid use disorder from 1990 to 2021: a statistical analysis of incidence, mortality, and disability-adjusted life years. BMC Public Health. 2025, 25(1):1988. (This study has been cited in the Introduction)
4. Please explain how you addressed multiple testing through an appropriately rigorous statistical threshold and indicate this in the methods section	As this study did not involve repeated hypothesis testing across multiple comparisons, issues of multiple testing were not considered applicable.
5. Please declare to what extent have AI chatbots been used in developing your paper and to which parts of the paper did they contribute	AI chatbots were used only as a language editing tool to improve grammar and clarity of expression. They did not contribute to study design, data analysis, interpretation, or conclusions. All scientific content was conceived, executed, and validated by the authors.

Table S2. Incidence, ASIRs and their changes in drug use disorders across SDI levels and GBD regions from 1990 to 2021

	Number of incident cases			ASIR (per 100,000 population)		
	1990	2021	Change (%)	1990	2021	Change (%)
	Both (95%UI)	Both (95%UI)		Both (95%UI)	Both (95%UI)	
**SDI**						
Middle	3248325.87 (2786171.41-3721937.43)	3972366.84 (3365469.48-4603391.54)	22.29	178.51 (152.88-204.74)	155.19 (131.25-179.27)	-13.06
High	2543678.43 (2151252.51-2950157.90)	3395020.04 (2984731.74-3860119.75)	33.47	284.65 (242.42-330.56)	350.90 (307.36-400.20)	23.27
Low-middle	1346208.35 (1131388.87-1591129.29)	2606009.21 (2213505.30-3032278.51)	93.58	124.75 (105.84-145.45)	130.55 (110.96-151.41)	4.65
High-middle	2429005.78 (2073812.92-2807202.02)	2464882.33 (2080289.07-2880813.63)	1.48	213.21 (181.57-246.44)	189.65 (161.53-218.09)	-11.05
Low	467472.97 (388093.67-557289.15)	1160433.64 (968967.83-1371410.00)	148.24	107.37 (88.96-125.35)	110.82 (92.59-128.79)	3.22
**GBD regions**						
South Asia	1239652.37 (1023324.10-1479405.10)	2597580.43 (2168940.58-3042955.00)	109.54	120.99 (100.92-142.05)	131.41 (109.78-153.28)	8.62
East Asia	3013584.47 (2546148.50-3494623.58)	2553499.27 (2134777.93-3029328.25)	-15.27	218.15 (184.94-253.87)	173.93 (146.09-204.63)	-20.27
High-income North America	1021790.71 (877231.38-1180896.04)	1713708.00 (1503719.89-1941995.90)	67.72	366.58 (312.82-426.29)	520.07 (454.13-592.82)	41.87
Western Europe	1096631.51 (932290.93-1271152.94)	1158666.62 (1007512.77-1338089.27)	5.66	290.50 (248.87-336.73)	302.00 (262.87-348.16)	3.96
Southeast Asia	659125.17 (543434.94-779425.38)	1049717.45 (869353.95-1231732.54)	59.26	137.34 (113.22-161.97)	141.48 (116.93-166.01)	3.01
North Africa and Middle East	418595.28 (350340.23-495003.52)	950174.79 (796661.95-1121367.53)	126.99	134.12 (112.41-158.09)	143.52 (120.87-169.07)	7.01
Eastern Europe	592543.40 (506283.87-679473.55)	529776.02 (457123.96-607119.51)	-10.59	262.36 (224.86-300.18)	275.72 (238.80-312.90)	5.09
Tropical Latin America	285331.43 (239089.97-346021.71)	422225.68 (359194.35-486314.89)	47.98	174.68 (146.99-208.86)	180.40 (153.43-207.80)	3.27
Eastern Sub-Saharan Africa	167665.02 (135071.42-206476.80)	417961.56 (340921.71-513395.63)	149.28	98.94 (81.32-117.53)	101.09 (83.77-119.60)	2.17
Western Sub-Saharan Africa	142236.43 (117503.74-169016.31)	398797.57 (333659.12-469311.07)	180.38	88.51 (72.58-106.10)	94.68 (79.60-111.36)	6.98
Central Latin America	226444.44 (191216.67-265255.17)	384443.57 (323943.49-446656.30)	69.77	141.32 (118.00-165.85)	144.04 (121.38-167.38)	1.93
High-income Asia Pacific	388116.24 (318787.47-468376.35)	324897.10 (268440.10-387744.63)	-16.29	208.76 (170.85-253.22)	204.38 (168.19-247.27)	-2.1
Central Europe	221416.63 (184626.35-261255.46)	194938.56 (163006.54-228660.21)	-11.96	175.85 (147.21-208.16)	184.24 (155.27-214.63)	4.77
Central Asia	112609.93 (94819.49-133623.65)	165880.08 (140144.05-194128.96)	47.31	165.80 (140.28-195.03)	169.72 (143.57-197.09)	2.36
Central Sub-Saharan Africa	50759.56 (41524.94-61585.09)	138352.33 (114528.91-166973.53)	172.56	107.56 (89.53-127.51)	110.05 (91.77-129.82)	2.31

Southern Sub-Saharan Africa	82455.01 (71109.64-96529.24)	136354.46 (115591.92-157588.06)	65.37	161.58 (139.28-185.37)	161.51 (137.31-186.47)	-0.04
Southern Latin America	92481.98 (77701.93-108917.13)	135147.98 (114721.72-157009.46)	46.13	185.95 (156.40-219.07)	196.13 (167.54-227.34)	5.48
Australasia	100309.47 (87560.17-117258.96)	123722.95 (106674.83-141155.62)	23.34	477.15 (415.54-555.64)	425.48 (369.38-483.04)	-10.83
Andean Latin America	53597.27 (45090.88-62340.76)	102256.01 (86119.97-118551.78)	90.79	144.70 (120.69-169.42)	147.25 (123.64-171.11)	1.76
Caribbean	66652.31 (54650.48-81484.92)	86450.43 (70519.18-104743.81)	29.7	180.64 (149.44-218.09)	180.09 (147.21-220.40)	-0.31
Oceania	11457.63 (9044.73-14625.12)	24811.51 (20002.28-30730.38)	116.55	172.19 (139.24-211.99)	173.25 (141.60-212.00)	0.61

Table S3. Prevalence, ASPRs and their changes in drug use disorders across SDI levels and GBD regions from 1990 to 2021

	**Number of prevalent cases**			**ASPR (per 100,000 population)**		
	**1990**	**2021**	**Change (%)**	**1990**	**2021**	**Change (%)**
	Both (95%UI)	Both (95%UI)		Both (95%UI)	Both (95%UI)	
**SDI**						
High	11716160.99 (10205716.44-13653997.40)	19257655.43 (17598069.60-21398277.69)	64.37	1282.80 (1114.26-1499.92)	1897.69 (1710.93-2137.33)	47.93
Middle	12797455.76 (10927813.40-15186456.21)	13859822.73 (11976147.93-16249385.03)	8.3	677.71 (589.07-788.40)	552.53 (475.41-653.60)	-18.47
High-middle	9136947.24 (7931598.51-10525727.92)	8255012.13 (7332623.83-9380593.97)	-9.65	779.94 (678.13-894.16)	667.46 (584.48-771.03)	-14.42
Low-middle	4423812.80 (3635044.69-5541738.13)	8049658.87 (6825501.98-9817912.44)	81.96	398.90 (337.37-486.57)	395.86 (339.37-479.11)	-0.76
Low	1512866.21 (1193634.80-1960808.37)	3654503.76 (2931631.00-4700261.25)	141.56	336.54 (276.97-420.79)	335.76 (278.02-416.83)	-0.23
**GBD regions**						
High-income North America	5833558.00 (5056468.62-6765849.21)	12918133.53 (11781271.57-14224633.86)	121.45	1997.89 (1722.10-2324.30)	3668.01 (3323.49-4067.36)	83.59
East Asia	11677286.45 (9927237.10-13860436.26)	8012070.41 (6895063.34-9433061.45)	-31.39	810.69 (697.85-954.84)	589.83 (494.67-703.92)	-27.24
South Asia	3970037.67 (3191740.47-5120145.25)	7844210.59 (6513450.04-9819055.31)	97.59	380.92 (311.84-479.25)	391.33 (327.33-483.68)	2.73
Western Europe	4345994.14 (3823479.54-4942184.41)	4519736.55 (4106574.42-5029749.49)	4	1126.22 (986.69-1287.06)	1201.17 (1081.17-1351.18)	6.66
Southeast Asia	2604146.43 (2060760.35-3282541.04)	3872468.94 (3139748.22-4776982.49)	48.7	519.61 (419.67-644.87)	524.30 (424.34-649.16)	0.9
North Africa and Middle East	1233270.01 (1038866.10-1459416.92)	2771196.58 (2422239.49-3187679.94)	124.7	378.78 (326.81-439.00)	422.68 (369.99-485.49)	11.59
Tropical Latin America	1477411.31 (1185378.47-1860113.11)	2099771.44 (1788693.83-2510048.37)	42.13	903.62 (741.68-1122.15)	888.21 (750.00-1066.61)	-1.7
Eastern Europe	2199136.69 (1915744.22-2525300.56)	1935373.74 (1732723.49-2177484.65)	-11.99	963.68 (829.48-1114.46)	1041.24 (908.44-1198.50)	8.05

Central Latin America	823981.15 (699123.08-993826.22)	1420513.92 (1237211.37-1635360.00)	72.4	489.62 (422.16-578.32)	529.59 (460.90-609.70)	8.16
Eastern Sub-Saharan Africa	580466.02 (440470.05-776486.16)	1411567.64 (1067353.55-1896497.99)	143.18	327.74 (260.28-419.69)	325.17 (257.73-422.17)	-0.78
High-income Asia Pacific	1466310.67 (1220529.85-1868929.42)	1203526.58 (1028637.41-1489142.38)	-17.92	798.76 (660.32-1022.92)	781.29 (644.13-995.33)	-2.19
Western Sub-Saharan Africa	399419.03 (321302.92-504249.00)	1065773.81 (875503.32-1332478.03)	166.83	234.19 (195.34-284.58)	236.64 (199.54-285.92)	1.05
Central Europe	773851.05 (645712.91-928214.92)	644902.46 (569858.99-745988.94)	-16.66	631.15 (523.55-762.89)	662.66 (571.49-776.46)	4.99
Southern Latin America	365413.37 (319153.43-423108.36)	565190.58 (505940.75-640496.67)	54.67	729.13 (636.60-843.34)	815.88 (730.49-926.72)	11.9
Central Asia	389626.53 (322331.49-477058.27)	563159.84 (480004.35-667119.29)	44.54	549.62 (461.22-662.75)	574.49 (483.62-687.87)	4.53
Southern Sub-Saharan Africa	344061.81 (294203.85-409545.70)	541071.64 (455101.09-657468.16)	57.26	670.31 (586.96-776.34)	639.35 (539.97-771.88)	-4.62
Australasia	476381.59 (426795.67-541881.51)	530216.75 (478541.18-594230.42)	11.3	2231.60 (1996.93-2541.13)	1819.35 (1632.77-2054.70)	-18.47
Central Sub-Saharan Africa	149823.17 (114081.12-202617.63)	403026.92 (309609.12-542840.80)	169	297.75 (238.08-387.13)	306.32 (246.24-397.68)	2.88
Caribbean	279259.91 (216019.36-366665.10)	355322.75 (277254.27-455909.08)	27.24	727.81 (576.21-930.60)	733.78 (568.30-946.83)	0.82
Andean Latin America	184910.80 (153483.58-224206.91)	339995.28 (286939.55-407831.52)	83.87	477.19 (404.11-573.00)	480.27 (406.37-576.63)	0.65
Oceania	46273.43 (32920.60-64389.70)	98706.44 (72689.38-133720.37)	113.31	668.94 (495.56-902.66)	672.72 (503.93-893.96)	0.57

Table S4. Deaths, ASMR and their changes in drug use disorders across SDI levels and GBD regions from 1990 to 2021

	Number of deaths			ASMR (per 100,000 population)		
	**1990**	**2021**	**Change (%)**	**1990**	**2021**	**Change (%)**
	Both (95%UI)	Both (95%UI)		Both (95%UI)	Both (95%UI)	
**SDI**						
High	11914.38 (11516.25-12297.59)	87936.65 (80866.65-96448.75)	638.07	1.22 (1.18-1.26)	7.07 (6.54-7.71)	478.86
Middle	27226.80 (24148.84-30552.97)	20598.26 (18269.64-22919.29)	-24.35	1.82 (1.62-2.04)	0.78 (0.69-0.86)	-57.26
High-middle	17164.46 (15855.16-18619.53)	14696.13 (13621.20-15642.69)	-14.38	1.56 (1.44-1.69)	0.97 (0.90-1.03)	-37.97
Low-middle	4069.82 (3508.81-4699.61)	10113.13 (8940.59-11258.61)	148.49	0.50 (0.43-0.58)	0.59 (0.53-0.66)	18.19
Low	1374.83 (1074.87-1807.38)	3888.33 (2931.97-4760.14)	182.82	0.45 (0.35-0.59)	0.51 (0.40-0.62)	12.58
**GBD regions**						
High-income North America	6124.50 (5798.37-6478.37)	74450.77 (67591.35-82621.99)	1115.62	1.93 (1.83-2.04)	18.42 (16.81-20.33)	855.33

East Asia	31827.69 (27799.72-36448.59)	11987.35 (9783.26-14384.91)	-62.34	2.69 (2.35-3.08)	0.69 (0.57-0.83)	-74.2
Western Europe	5466.82 (5320.86-5626.72)	11741.86 (11152.34-12283.42)	114.78	1.32 (1.28-1.36)	2.28 (2.19-2.37)	72.72
South Asia	4422.27 (3818.90-5076.91)	10802.93 (9459.56-12215.64)	144.28	0.59 (0.50-0.67)	0.64 (0.56-0.72)	9.69
Eastern Europe	5941.23 (5528.97-6396.39)	7924.00 (7229.05-8651.16)	33.37	2.38 (2.22-2.57)	3.41 (3.11-3.73)	42.78
North Africa and Middle East	3052.27 (2695.10-3587.59)	7579.37 (6665.44-8629.18)	148.32	1.20 (1.05-1.40)	1.24 (1.10-1.42)	3.81
Southeast Asia	969.24 (833.75-1157.57)	2336.48 (1944.56-2865.15)	141.06	0.28 (0.24-0.33)	0.34 (0.29-0.42)	23.45
Eastern Sub-Saharan Africa	600.02 (402.92-864.82)	1919.88 (1290.83-2497.21)	219.97	0.60 (0.40-0.86)	0.67 (0.46-0.86)	11.49
Australasia	475.96 (443.49-509.02)	1521.23 (1361.82-1686.29)	219.61	2.17 (2.03-2.33)	4.41 (3.94-4.89)	102.86
Tropical Latin America	129.79 (122.76-137.00)	1417.52 (1327.08-1528.98)	992.13	0.09 (0.09-0.10)	0.57 (0.54-0.62)	523.99
Central Latin America	515.95 (494.35-541.01)	1136.99 (1005.81-1286.83)	120.37	0.37 (0.35-0.38)	0.43 (0.38-0.48)	16.33
Central Europe	814.79 (740.98-892.70)	975.63 (901.55-1058.49)	19.74	0.61 (0.56-0.67)	0.72 (0.67-0.78)	17.31
Southern Sub-Saharan Africa	508.72 (432.22-568.34)	931.89 (848.88-1039.24)	83.18	1.31 (1.10-1.46)	1.32 (1.20-1.45)	0.62
Central Asia	298.62 (259.27-342.90)	888.31 (744.25-1037.80)	197.47	0.51 (0.44-0.58)	0.92 (0.78-1.08)	81.97
High-income Asia Pacific	205.53 (195.55-216.15)	420.93 (389.96-448.21)	104.8	0.11 (0.10-0.11)	0.17 (0.16-0.18)	62.41
Central Sub-Saharan Africa	118.72 (70.08-178.92)	389.62 (223.82-595.06)	228.18	0.32 (0.19-0.49)	0.38 (0.22-0.58)	19.48
Andean Latin America	114.18 (97.76-131.08)	346.94 (284.00-426.40)	203.85	0.36 (0.31-0.42)	0.52 (0.43-0.64)	44.41
Caribbean	67.34 (60.07-73.88)	202.43 (171.07-235.57)	200.61	0.21 (0.19-0.23)	0.40 (0.34-0.47)	90.44
Southern Latin America	34.49 (31.45-37.49)	155.64 (138.57-175.81)	351.28	0.07 (0.07-0.08)	0.20 (0.18-0.23)	171.4
Western Sub-Saharan Africa	74.49 (52.78-97.08)	129.22 (86.46-167.79)	73.47	0.07 (0.05-0.10)	0.05 (0.04-0.07)	-27.01
Oceania	11.86 (7.55-16.92)	18.94 (13.73-26.02)	59.62	0.23 (0.15-0.33)	0.16 (0.12-0.22)	-29.56

Table S5. DALYs, ASDR and their changes in drug use disorders across SDI levels and GBD regions from 1990 to 2021

Characteristics	Number of DALYs			ASDR (per 100,000 population)		
	1990	2021	Change (%)	1990	2021	Change (%)
	Both (95%UI)	Both (95%UI)		Both (95%UI)	Both (95%UI)	
**SDI**						

High	2103749.77 (1644036.12-2549563.34)	8333571.68 (7048826.98-9607578.71)	296.13	222.34 (173.15-269.45)	752.61 (630.61-872.87)	238.5
Middle	3349720.01 (2667547.56-3959105.86)	2912744.35 (2307572.55-3479456.77)	-13.05	191.36 (154.29-223.58)	112.90 (89.50-135.09)	-41
High-middle	2435884.13 (1915885.92-2897939.49)	2069598.04 (1664133.27-2459504.38)	-15.04	211.32 (166.86-251.33)	153.92 (122.22-185.21)	-27.16
Low-middle	771786.99 (605433.85-962183.98)	1593534.49 (1239892.55-1935327.47)	106.47	74.83 (59.16-91.85)	81.28 (63.74-98.29)	8.62
Low	244557.68 (188283.92-309824.96)	645864.91 (497371.00-797894.62)	164.1	61.21 (47.94-76.72)	66.47 (51.71-81.05)	8.6
**GBD regions**						
High-income North America	1078039.60 (831794.64-1308306.68)	6807462.52 (5764548.81-7844234.40)	531.47	352.05 (270.73-427.06)	1836.34 (1547.74-2122.45)	421.62
East Asia	3614386.80 (2897066.28-4299278.97)	1736106.38 (1342136.60-2120314.19)	-51.97	268.49 (217.53-316.18)	117.23 (89.99-144.50)	-56.34
South Asia	686910.37 (536014.83-854810.86)	1521317.39 (1197076.90-1867550.53)	121.47	71.11 (56.10-87.26)	78.68 (62.13-95.44)	10.65
Western Europe	847230.94 (685035.12-1005491.98)	1163021.52 (975083.75-1348151.24)	37.27	211.34 (170.89-250.97)	276.35 (230.25-322.25)	30.76
North Africa and Middle East	453680.83 (353056.32-564082.79)	1059497.12 (850242.07-1265837.28)	133.53	148.73 (117.32-181.45)	161.95 (129.92-193.35)	8.89
Eastern Europe	768334.28 (614076.36-907539.44)	834080.76 (709961.99-965280.97)	8.56	323.60 (257.79-382.26)	403.12 (337.84-468.51)	24.57
Southeast Asia	324375.96 (232647.55-428994.35)	527235.11 (390182.39-674457.48)	62.54	68.29 (50.24-88.66)	71.21 (52.71-91.13)	4.28
Tropical Latin America	162061.67 (110807.96-213561.19)	312556.23 (238401.54-385074.80)	92.86	102.09 (71.29-133.35)	129.66 (98.32-160.11)	27.01
Eastern Sub-Saharan Africa	86968.65 (66611.07-111945.93)	240519.45 (186124.59-298670.85)	176.56	60.83 (47.07-78.07)	66.58 (51.76-82.03)	9.46
Central Latin America	139214.36 (105074.32-176664.83)	237478.51 (184801.18-294877.94)	70.58	86.88 (65.99-109.39)	88.41 (68.83-109.78)	1.76
Central Asia	92368.72 (67553.27-118438.49)	158577.51 (121995.31-192699.86)	71.68	135.73 (100.13-171.36)	158.77 (122.14-192.69)	16.97
Western Sub-Saharan Africa	60464.33 (42963.92-79862.20)	156481.76 (110916.00-206264.88)	158.8	37.90 (27.48-48.72)	37.27 (26.73-48.09)	-1.67
High-income Asia Pacific	166825.47 (118067.02-220263.36)	152624.14 (111895.91-194901.28)	-8.51	89.87 (63.71-119.15)	90.07 (65.11-117.63)	0.22
Australasia	79156.56 (63933.22-93223.68)	145715.77 (122595.56-167902.01)	84.09	365.23 (294.85-430.61)	464.24 (387.42-539.76)	27.11
Central Europe	124195.04 (97749.04-151799.02)	124133.78 (100101.75-148754.34)	-0.05	98.28 (77.04-120.40)	113.79 (90.95-137.08)	15.78
Southern Sub-Saharan Africa	82678.22 (64117.82-100809.10)	116852.81 (95212.64-138212.28)	41.33	175.43 (138.47-210.37)	143.29 (117.00-169.05)	-18.32
Southern Latin America	51692.88 (35509.65-68800.70)	78443.88 (56505.20-102079.77)	51.75	104.52 (72.10-139.02)	110.29 (79.08-143.92)	5.52
Central Sub-Saharan Africa	23760.59 (17783.58-30833.37)	70604.95 (51499.60-90811.21)	197.15	52.77 (39.86-67.88)	59.27 (43.57-75.47)	12.33
Andean Latin America	30542.19 (22396.51-38683.36)	64360.49 (50048.09-80101.98)	110.73	83.02 (62.22-104.32)	91.59 (71.54-113.89)	10.32
Caribbean	33153.26 (23763.25-42405.06)	45411.78 (34712.35-57359.80)	36.98	90.76 (65.78-116.08)	92.30 (70.37-116.99)	1.7

Oceania	4562.72 (3240.99-6143.33)	9679.65 (6942.86-12722.60)	112.15	71.33 (51.57-93.20)	68.81 (49.71-89.73)	-3.54

Table S6. Incidence, ASIRs and their changes in drug use disorders across 204 countries and territories from 1990 to 2021

Country	Number of incident cases			ASIR (per 100,000 population)		
	1990	2021	Change (%)	1990	2021	Change (%)
	Both (95%UI)	Both (95%UI)		Both (95%UI)	Both (95%UI)	
China	2932127.84 (2478008.36-3400135.92)	2451314.00 (2046472.04-2907370.53)	-16.4	219.22 (185.84-255.05)	173.24 (145.45-203.89)	-20.98
India	987866.27 (809057.29-1177349.31)	2047672.59 (1706130.84-2396268.76)	107.28	120.90 (100.32-142.46)	133.13 (111.12-155.54)	10.11
United States	914697.20 (779153.37-1061430.63)	1583449.64 (1384480.18-1793912.27)	73.11	362.84 (307.51-424.01)	531.19 (462.11-605.02)	46.4
Brazil	280196.70 (234685.38-340129.94)	411752.60 (350243.05-474141.24)	46.95	175.72 (147.79-210.23)	182.11 (154.74-209.69)	3.64
Russian Federation	435930.23 (372209.00-498234.16)	397455.19 (342434.66-458328.60)	-8.83	289.45 (247.71-331.17)	296.27 (256.86-339.41)	2.36
Indonesia	221045.13 (181857.85-263200.96)	381218.01 (314170.72-449546.29)	72.46	116.16 (96.43-137.64)	124.92 (102.86-147.64)	7.54
Pakistan	111970.71 (91924.39-134577.15)	276248.51 (226900.73-327962.98)	146.71	115.30 (96.49-135.90)	117.47 (97.87-138.61)	1.89
United Kingdom	189798.67 (158490.41-223556.96)	235230.00 (201314.42-274067.00)	23.94	352.66 (291.14-421.94)	390.75 (329.91-464.46)	10.8
Bangladesh	119713.69 (99955.83-143700.35)	234784.69 (196162.02-281052.47)	96.12	131.68 (111.35-156.92)	136.65 (114.37-164.56)	3.77
Germany	202103.38 (165950.63-243172.76)	212160.46 (183311.16-253506.58)	4.98	256.69 (214.10-304.99)	287.31 (247.52-339.06)	11.93
Japan	270374.33 (220451.89-324680.31)	207715.97 (171972.48-247140.30)	-23.17	207.25 (171.04-251.07)	199.69 (164.06-241.59)	-3.65
Iran	83719.59 (70707.40-97078.63)	186012.98 (156957.36-216144.49)	122.19	167.84 (141.50-195.18)	200.93 (171.25-230.69)	19.71
Mexico	112450.60 (94139.11-133265.03)	184185.13 (154534.85-215257.92)	63.79	135.36 (112.13-159.30)	133.85 (112.31-155.56)	-1.12
Nigeria	64656.82 (53140.38-76787.89)	179657.43 (150415.86-212240.72)	177.86	84.35 (69.37-100.41)	91.75 (76.79-107.69)	8.77
Vietnam	103998.98 (84699.28-125903.69)	179127.53 (147921.72-210830.50)	72.24	153.87 (127.16-184.81)	173.11 (143.44-203.38)	12.51
France	155115.92 (131122.63-181959.63)	170474.15 (144815.96-199426.68)	9.9	272.02 (230.47-318.12)	286.37 (241.95-336.20)	5.28
Philippines	88956.44 (73244.82-106812.26)	160896.09 (133310.67-190797.27)	80.87	141.52 (117.79-166.11)	133.34 (110.60-156.90)	-5.78
Egypt	62825.89 (51834.03-75555.46)	133380.12 (111326.80-161255.75)	112.3	120.57 (99.49-144.68)	126.23 (105.51-152.24)	4.7
Spain	130119.77 (114643.47-146330.52)	131871.79 (114582.28-152937.65)	1.35	331.05 (290.53-373.38)	341.22 (297.81-384.91)	3.07
Canada	106880.24 (93914.91-119849.78)	130071.09 (115623.44-149937.65)	21.7	401.71 (356.38-446.00)	414.66 (370.16-468.42)	3.22

Italy	169202.92 (140775.16-203011.73)	129112.92 (107184.21-151356.52)	-23.69	297.99 (245.44-364.24)	252.43 (209.57-299.14)	-15.29
Turkey	70214.78 (57948.23-84050.50)	111010.21 (90637.03-134248.53)	58.1	123.73 (102.54-148.36)	123.64 (101.21-149.46)	-0.08
Thailand	101868.74 (84348.56-120291.63)	106412.99 (88074.95-124574.99)	4.46	157.94 (130.72-186.86)	165.55 (138.14-193.87)	4.82
Australia	87440.56 (75998.25-103301.95)	104379.27 (89570.16-120390.32)	19.37	501.31 (435.20-589.14)	429.39 (370.03-491.99)	-14.35
South Africa	63942.37 (55421.10-73871.67)	103854.46 (88293.22-119979.36)	62.42	170.72 (147.24-195.54)	170.56 (144.68-197.27)	-0.1
South Korea	109176.25 (88182.92-133205.52)	103830.92 (84624.17-124886.00)	-4.9	212.13 (170.20-260.13)	213.28 (173.32-261.87)	0.54
Ethiopia	41393.65 (33408.62-51296.64)	103507.63 (84506.60-126272.73)	150.06	94.45 (78.07-112.65)	95.89 (78.94-113.56)	1.53
Colombia	51922.07 (44040.84-60797.68)	92195.19 (78321.81-106817.74)	77.56	154.51 (129.95-181.10)	181.54 (154.32-209.98)	17.49
Ukraine	106951.24 (90761.76-122913.50)	90757.16 (77443.68-104007.54)	-15.14	202.89 (171.24-234.26)	223.21 (191.24-253.42)	10.02
Democratic Republic of the Congo	34512.16 (28177.04-41973.34)	89804.20 (73930.22-107987.71)	160.21	106.14 (87.53-125.52)	107.19 (89.53-126.61)	0.99
Myanmar	62835.12 (51195.58-75238.84)	89376.14 (72473.32-109098.78)	42.24	149.27 (123.01-176.16)	150.76 (122.37-183.85)	1
Argentina	57589.85 (47993.56-68296.01)	84881.64 (71511.19-100693.64)	47.39	177.24 (147.72-209.70)	180.41 (152.92-212.56)	1.79
Saudi Arabia	20397.08 (16864.57-24555.17)	67598.00 (54756.43-81694.98)	231.41	133.90 (110.18-160.14)	139.46 (114.68-165.34)	4.15
Algeria	31160.15 (25874.28-37428.50)	66958.77 (55938.15-79130.15)	114.89	136.97 (113.26-162.28)	147.58 (123.66-172.72)	7.75
Poland	71806.02 (59065.26-87032.81)	65220.82 (53745.25-77179.59)	-9.17	190.44 (157.33-231.95)	189.07 (155.82-225.65)	-0.72
Tanzania	26832.07 (21422.46-32624.80)	62731.40 (50443.47-78904.02)	133.79	112.57 (92.73-133.96)	112.55 (92.45-136.34)	-0.01
Sudan	21962.85 (18150.29-26234.99)	57041.60 (47455.69-66916.87)	159.72	124.56 (103.27-147.70)	132.59 (109.68-155.88)	6.44
Morocco	32528.10 (27138.52-38702.27)	56213.61 (46846.21-65314.87)	72.82	133.60 (111.88-157.64)	143.89 (120.19-166.97)	7.7
Iraq	20795.07 (17397.76-24992.27)	56089.41 (47093.37-66755.48)	169.72	126.62 (106.12-150.92)	129.19 (107.96-153.92)	2.03
Peru	30500.11 (25482.30-35625.36)	55811.25 (46745.52-65445.39)	82.99	144.85 (119.17-171.01)	145.10 (121.44-169.98)	0.17
Taiwan (Province of China)	43980.68 (36049.51-51966.80)	53764.15 (44655.84-64608.59)	22.24	196.10 (162.21-230.21)	219.01 (182.80-259.40)	11.69
Malaysia	27698.97 (22872.28-33151.41)	53637.22 (44262.88-63383.62)	93.64	155.86 (129.71-186.08)	152.58 (127.21-179.50)	-2.1
Uzbekistan	30017.70 (25023.39-36069.13)	53261.56 (44312.57-62803.90)	77.43	150.32 (125.46-179.43)	150.41 (125.37-176.30)	0.06
North Korea	37475.94 (31453.05-44172.92)	48421.12 (40365.48-56669.54)	29.21	173.07 (144.14-203.75)	169.38 (141.36-197.93)	-2.13
Uganda	17379.53 (13345.18-22252.29)	44925.55 (36389.62-54316.52)	158.5	109.89 (88.63-133.70)	108.32 (89.77-127.62)	-1.43
Kazakhstan	33155.62 (27678.41-38810.59)	44527.92 (38094.58-51851.02)	34.3	198.26 (165.84-231.32)	234.72 (201.48-272.60)	18.39

Kenya	15071.35 (12361.95-18183.00)	43656.39 (35796.44-52344.07)	189.66	79.16 (64.81-94.47)	86.25 (71.30-101.66)	8.96
Chile	28218.11 (23933.33-32945.87)	43113.67 (36879.07-49718.61)	52.79	199.38 (169.04-233.05)	234.74 (201.34-270.02)	17.74
Afghanistan	11785.81 (9855.25-13937.92)	41366.69 (34769.75-47786.12)	250.99	142.24 (119.43-166.58)	145.72 (122.13-169.24)	2.45
Yemen	12935.25 (10575.85-15853.68)	40444.64 (33244.46-47878.84)	212.67	121.77 (100.31-146.82)	126.03 (104.33-147.84)	3.5
Netherlands	42116.81 (35446.79-49236.67)	38768.37 (32772.50-45542.85)	-7.95	278.83 (237.35-325.14)	254.29 (216.93-299.97)	-8.8
Nepal	19424.42 (16046.92-23454.18)	37835.74 (30935.99-45439.79)	94.78	111.81 (92.68-133.00)	116.05 (95.99-137.69)	3.79
Sri Lanka	30199.48 (25315.46-35795.38)	36139.44 (29886.15-42838.06)	19.67	166.06 (140.11-195.06)	159.97 (132.29-190.28)	-3.67
Venezuela	25264.23 (20955.00-30237.24)	35517.70 (29368.85-42142.90)	40.58	135.24 (111.11-159.55)	130.70 (107.93-155.33)	-3.35
Ghana	12113.28 (9951.94-14558.99)	32794.89 (26989.58-39050.85)	170.74	93.85 (76.82-112.78)	98.94 (81.71-117.43)	5.43
Angola	9885.33 (8100.94-11982.34)	32402.65 (26666.61-39443.03)	227.79	110.38 (91.15-131.46)	115.22 (95.43-135.90)	4.39
Mozambique	11604.19 (9141.95-14821.60)	31127.06 (24928.24-39073.04)	168.24	98.47 (80.36-119.94)	111.99 (91.35-133.78)	13.74
Cameroon	8170.59 (6743.26-9870.50)	29380.77 (24540.15-35021.20)	259.59	96.27 (79.55-115.97)	104.41 (87.77-123.99)	8.45
Belgium	26028.38 (21420.54-31139.88)	28331.19 (24265.07-32851.99)	8.85	267.81 (220.41-317.74)	276.09 (236.35-317.05)	3.09
Madagascar	10712.33 (8426.62-13670.74)	28279.99 (22499.52-35568.37)	163.99	100.57 (81.14-123.71)	101.36 (82.12-123.19)	0.78
Sweden	19390.60 (15868.65-23408.60)	28009.47 (23688.22-33267.67)	44.45	227.28 (186.10-274.03)	296.81 (251.02-351.21)	30.59
Switzerland	28094.07 (23806.80-32769.75)	27908.54 (23542.32-32803.38)	-0.66	424.88 (360.29-502.32)	353.22 (300.02-411.79)	-16.87
Ecuador	14335.92 (11986.77-16885.69)	27761.95 (23255.32-32275.80)	93.65	143.88 (120.46-169.12)	148.09 (124.09-172.49)	2.92
Romania	32886.89 (27013.60-39163.99)	27101.45 (21944.06-32782.18)	-17.59	138.34 (113.39-164.49)	143.39 (117.66-171.11)	3.65
Cote d'Ivoire	9638.97 (7873.73-11649.58)	25340.18 (20841.62-29988.00)	162.89	94.25 (77.23-113.54)	99.94 (83.27-117.52)	6.04
United Arab Emirates	3612.38 (2882.02-4349.21)	24768.29 (19431.62-30978.85)	585.65	163.77 (134.26-196.41)	183.05 (154.37-214.72)	11.77
Guatemala	11588.19 (9796.25-13572.14)	24639.28 (20507.68-29976.76)	112.62	177.72 (149.19-207.64)	153.64 (127.82-185.75)	-13.55
Cambodia	12706.67 (10298.74-15360.04)	24129.49 (19971.38-28368.18)	89.9	134.50 (111.19-159.63)	134.05 (111.75-156.45)	-0.33
Austria	19084.62 (16035.41-22641.76)	23289.67 (19746.44-27027.40)	22.03	240.77 (201.93-284.34)	284.40 (241.97-325.95)	18.12
Haiti	9629.36 (7799.05-12027.24)	23100.37 (18347.14-28895.99)	139.9	156.71 (129.64-189.16)	167.50 (134.03-206.61)	6.88
Czech Republic	23904.35 (19816.79-28362.41)	22707.91 (19260.71-26411.59)	-5.01	232.00 (191.49-275.15)	253.86 (216.55-294.07)	9.42
Zimbabwe	12846.78 (10466.79-15768.48)	21372.25 (17528.91-25817.73)	66.36	144.22 (120.57-171.48)	143.83 (121.11-169.25)	-0.27

Israel	12032.89 (10080.66-14352.50)	21008.42 (17369.97-25163.14)	74.59	244.60 (204.60-292.57)	229.72 (189.86-276.96)	-6.09
Belarus	22985.96 (19394.10-26938.70)	20088.05 (16889.21-23740.15)	-12.61	220.27 (184.70-259.20)	223.93 (189.66-266.36)	1.66
Portugal	23722.21 (19864.55-28200.75)	20004.91 (16401.18-23731.08)	-15.67	231.70 (194.34-275.54)	210.47 (174.54-246.85)	-9.16
New Zealand	12868.92 (10851.00-15384.37)	19343.68 (16242.91-22853.93)	50.31	359.61 (301.48-431.46)	402.36 (337.68-483.62)	11.89
Malawi	8689.08 (6864.88-11136.85)	19293.62 (15329.33-24508.60)	122.04	99.05 (80.32-121.34)	100.29 (82.00-121.75)	1.25
Dominican Republic	10501.91 (8693.53-12731.34)	19031.92 (15462.40-23429.03)	81.22	144.72 (119.32-173.82)	164.23 (132.97-203.79)	13.48
Somalia	6957.31 (5412.26-9025.49)	18778.01 (14651.07-24133.70)	169.9	96.86 (78.24-119.10)	93.68 (75.99-115.21)	-3.29
Greece	20372.09 (16560.41-24693.06)	18711.03 (15449.55-22594.97)	-8.15	195.62 (159.62-237.45)	206.61 (172.65-245.49)	5.62
Bolivia	8761.24 (7453.38-10236.79)	18682.82 (15676.66-21568.57)	113.24	145.43 (123.27-169.65)	151.36 (127.47-174.13)	4.07
Zambia	7891.69 (6478.73-9527.10)	18678.96 (15529.75-22577.09)	136.69	108.32 (89.46-127.37)	101.88 (85.13-120.37)	-5.95
Syria	12984.51 (10910.31-15577.89)	18563.04 (15509.96-21937.32)	42.96	120.58 (100.14-144.42)	127.07 (105.14-151.63)	5.38
Papua New Guinea	7095.61 (5602.47-9029.72)	18559.32 (14801.90-23158.28)	161.56	170.90 (137.52-210.65)	172.16 (140.02-212.43)	0.74
Cuba	22046.94 (17673.24-27759.79)	18382.77 (14740.68-22544.65)	-16.62	182.69 (145.11-233.00)	179.55 (142.08-228.61)	-1.72
Mali	6216.17 (5063.26-7520.95)	17889.80 (14747.40-21445.21)	187.79	89.39 (73.21-107.69)	92.22 (76.07-109.96)	3.16
Burkina Faso	6454.43 (5278.64-7784.50)	17815.81 (14769.55-20995.51)	176.02	88.41 (72.24-105.79)	94.04 (77.89-110.42)	6.36
Tunisia	10366.63 (8553.30-12343.10)	17637.40 (14530.43-21130.80)	70.14	129.46 (106.81-154.82)	141.78 (117.24-169.16)	9.52
Norway	11952.55 (9896.14-14313.44)	17482.09 (14912.84-20423.94)	46.26	274.49 (226.91-329.46)	330.72 (281.11-384.56)	20.49
Niger	5669.80 (4610.36-6827.50)	17467.68 (14495.48-21100.00)	208.08	90.13 (73.73-108.11)	90.93 (75.51-108.49)	0.9
Jordan	4415.40 (3654.02-5264.94)	17124.72 (14286.42-20464.27)	287.84	127.30 (105.42-151.36)	126.83 (105.25-152.06)	-0.37
Azerbaijan	11731.55 (9763.16-13901.19)	17070.38 (14230.32-20410.80)	45.51	155.46 (130.23-185.35)	152.09 (126.70-180.41)	-2.17
Denmark	16580.68 (14210.52-19715.87)	16671.62 (13964.32-19466.11)	0.55	314.51 (269.79-370.07)	309.32 (258.96-361.01)	-1.65
Honduras	5942.50 (4965.74-7124.39)	16411.18 (13531.92-19666.25)	176.17	146.36 (122.61-171.15)	155.95 (129.61-185.71)	6.55
Rwanda	6648.80 (5237.91-8436.38)	15807.98 (12418.16-19963.23)	137.76	103.39 (84.34-125.22)	113.47 (91.46-139.62)	9.75
Hungary	18262.52 (15246.65-21456.75)	15690.60 (13014.26-18602.53)	-14.08	178.19 (149.25-209.67)	182.80 (152.70-214.08)	2.58
Ireland	11009.96 (9307.22-12890.12)	15612.61 (13340.14-18040.54)	41.8	297.15 (250.84-348.47)	332.36 (285.03-380.24)	11.85
Finland	14267.64 (11838.08-16821.44)	15464.61 (13426.75-17942.58)	8.39	274.64 (228.45-319.94)	317.56 (277.28-365.39)	15.63

Tajikistan	7582.44 (6338.45-9087.92)	15061.86 (12569.79-17927.20)	98.64	152.95 (128.12-182.48)	146.21 (122.00-172.62)	-4.4
Serbia	15475.73 (12550.11-18643.51)	14159.22 (11523.27-16836.21)	-8.51	158.63 (128.39-191.07)	162.80 (133.71-193.21)	2.63
Libya	5570.28 (4622.96-6664.88)	14071.79 (11590.68-16865.88)	152.62	149.96 (122.79-179.78)	171.09 (141.70-203.20)	14.09
Senegal	5746.99 (4689.63-6909.40)	14012.19 (11626.23-16581.96)	143.82	93.84 (77.01-112.38)	97.25 (80.89-115.52)	3.63
Chad	4254.80 (3483.57-5139.28)	12836.80 (10677.31-15387.94)	201.7	89.80 (73.35-107.63)	94.35 (78.29-112.53)	5.07
Singapore	7924.71 (6397.38-9682.50)	12248.39 (9769.35-14825.59)	54.56	215.83 (173.51-264.37)	215.00 (172.89-263.74)	-0.39
Burundi	4808.25 (3807.03-6121.19)	12091.78 (9607.13-15405.72)	151.48	99.18 (80.25-121.36)	96.81 (79.27-119.12)	-2.39
Benin	3585.92 (2922.83-4414.65)	11927.28 (9932.14-14232.93)	232.61	94.27 (77.09-114.07)	101.50 (84.95-120.11)	7.67
Bulgaria	14313.07 (12015.46-16870.40)	11657.72 (9912.97-13715.07)	-18.55	169.37 (142.16-199.71)	201.10 (172.81-230.79)	18.73
Kyrgyzstan	7141.06 (5964.26-8436.49)	11335.90 (9513.75-13346.87)	58.74	168.79 (141.72-197.38)	164.98 (139.12-193.72)	-2.26
Laos	5292.04 (4255.23-6572.33)	11216.82 (9238.52-13501.91)	111.96	137.85 (113.00-165.17)	141.88 (116.42-171.27)	2.92
Guinea	4372.56 (3569.65-5317.28)	10752.18 (8903.00-12843.55)	145.9	90.02 (73.38-109.04)	94.98 (78.77-112.57)	5.51
Paraguay	5134.73 (4350.75-5957.56)	10473.08 (8697.21-12289.49)	103.97	134.91 (113.56-156.78)	136.14 (113.05-159.40)	0.91
Lebanon	4129.19 (3413.07-4891.43)	9945.14 (8344.16-11662.09)	140.85	144.42 (119.49-171.38)	166.64 (140.35-193.43)	15.39
Slovakia	10706.73 (8946.92-12595.02)	9467.51 (7930.54-11175.56)	-11.57	199.51 (167.39-233.86)	187.66 (159.16-218.25)	-5.94
South Sudan	5539.24 (4364.73-7118.80)	9208.37 (7212.30-11954.05)	66.24	103.15 (82.96-126.36)	100.92 (82.20-123.14)	-2.16
Turkmenistan	5599.22 (4702.26-6689.58)	9185.60 (7672.30-10786.16)	64.05	156.48 (130.97-184.97)	170.94 (142.79-200.98)	9.24
Kuwait	2779.97 (2268.86-3340.94)	8843.06 (7093.56-10806.28)	218.1	141.95 (115.99-169.46)	150.77 (124.88-178.21)	6.21
Nicaragua	4708.75 (3923.02-5568.14)	8711.67 (7100.01-10359.46)	85.01	136.74 (113.89-162.14)	123.39 (101.19-147.01)	-9.76
Croatia	9935.53 (8343.55-11720.16)	8425.03 (7211.75-9838.94)	-15.2	204.91 (171.61-241.63)	227.22 (196.20-262.87)	10.89
El Salvador	5913.15 (4929.84-7005.44)	8408.79 (6976.11-10111.55)	42.2	121.53 (100.58-144.26)	127.65 (105.50-153.76)	5.04
Puerto Rico	10190.92 (8488.62-12255.52)	8127.86 (6889.31-9642.35)	-20.24	273.62 (228.54-327.72)	263.75 (219.01-318.10)	-3.61
Sierra Leone	3332.09 (2734.12-4050.29)	7831.71 (6529.29-9324.72)	135.04	92.95 (76.16-112.83)	95.67 (79.76-113.48)	2.92
Costa Rica	4484.33 (3669.55-5481.51)	7657.36 (6393.21-8960.39)	70.76	147.24 (120.73-177.30)	152.88 (128.15-178.57)	3.84
Eritrea	3180.94 (2500.93-4052.53)	7251.83 (5786.20-8942.86)	127.98	102.98 (83.34-125.94)	108.97 (89.04-131.92)	5.81
Oman	2400.14 (1957.51-2919.33)	7220.02 (5809.36-8788.89)	200.82	125.55 (103.73-150.25)	126.45 (104.45-149.99)	0.72

Uruguay	6669.62 (5574.04-7868.83)	7145.21 (6053.99-8316.38)	7.13	217.87 (181.46-256.93)	219.23 (186.17-255.97)	0.63
Togo	2544.99 (2080.41-3116.13)	7058.38 (5951.34-8323.63)	177.34	86.89 (70.69-105.52)	91.32 (77.08-107.44)	5.1
Palestine	2298.73 (1917.64-2801.96)	7038.60 (5814.70-8356.75)	206.2	130.23 (107.88-155.19)	132.97 (109.64-158.76)	2.1
Panama	4170.62 (3452.31-4975.82)	6717.29 (5454.85-8027.29)	61.06	167.81 (139.15-197.78)	153.60 (124.29-184.17)	-8.46
Lithuania	7779.75 (6641.32-9060.79)	6629.51 (5720.82-7593.97)	-14.79	208.15 (177.42-242.18)	273.86 (238.26-313.80)	31.57
Congo	2429.57 (1983.39-2951.15)	6609.58 (5472.56-7955.00)	172.05	115.39 (95.35-137.31)	121.40 (100.90-144.84)	5.21
Moldova	8681.64 (7278.37-10301.95)	6444.78 (5331.81-7673.83)	-25.77	194.81 (163.42-230.59)	178.35 (149.85-207.82)	-8.45
Jamaica	4720.60 (3751.07-5948.12)	5909.56 (4856.35-6956.54)	25.19	189.48 (152.72-234.22)	200.37 (163.85-237.74)	5.75
Mongolia	3343.31 (2775.01-4061.01)	5562.04 (4650.48-6521.82)	66.36	158.58 (132.60-187.03)	167.75 (140.57-196.33)	5.78
Liberia	1985.99 (1629.52-2433.36)	5470.19 (4477.00-6590.99)	175.44	96.27 (78.87-115.51)	102.54 (84.56-121.50)	6.51
Qatar	704.79 (567.70-868.69)	5396.16 (4283.82-6719.93)	665.64	128.49 (106.17-154.01)	129.62 (106.70-154.89)	0.89
Central African Republic	2491.65 (2042.35-3016.53)	5347.60 (4429.74-6485.63)	114.62	103.35 (85.69-122.96)	103.07 (86.20-122.28)	-0.28
Georgia	8369.16 (7038.16-9991.06)	5223.55 (4325.11-6175.84)	-37.59	149.51 (125.70-177.92)	153.61 (128.91-179.60)	2.74
Armenia	5669.88 (4777.45-6733.56)	4651.26 (3882.77-5492.61)	-17.97	161.57 (135.55-191.85)	157.96 (133.58-184.26)	-2.23
Albania	5344.14 (4324.23-6581.12)	4621.33 (3868.18-5424.94)	-13.53	155.31 (125.86-189.19)	175.38 (146.73-206.30)	12.92
Bosnia and Herzegovina	6745.07 (5456.51-8293.82)	4432.60 (3583.76-5355.82)	-34.28	138.78 (111.90-171.15)	140.19 (113.43-168.61)	1.02
Estonia	4028.20 (3501.58-4648.00)	4379.76 (3928.16-4953.10)	8.73	263.85 (228.82-304.36)	406.43 (367.40-454.31)	54.04
Slovenia	4207.54 (3460.36-5047.17)	4066.02 (3442.70-4746.81)	-3.36	213.59 (174.83-255.99)	236.58 (204.43-271.33)	10.77
Latvia	6186.37 (5323.12-7081.35)	4021.57 (3453.70-4664.26)	-34.99	237.76 (204.56-272.13)	249.90 (215.39-284.91)	5.11
Mauritania	1616.66 (1323.37-1955.43)	3778.97 (3133.23-4496.71)	133.75	93.93 (76.26-114.17)	98.44 (81.72-116.24)	4.8
Namibia	1689.69 (1385.66-2064.67)	3553.32 (2959.46-4192.53)	110.29	130.88 (109.07-156.12)	141.00 (117.61-165.36)	7.73
Macedonia	3137.86 (2584.40-3722.44)	3505.05 (2868.47-4164.80)	11.7	150.40 (123.92-178.64)	156.12 (128.87-184.03)	3.8
Botswana	1415.42 (1171.10-1733.35)	3322.13 (2774.43-3925.96)	134.71	120.99 (100.38-144.77)	127.37 (106.89-149.90)	5.27
Cyprus	1743.42 (1429.66-2091.21)	3011.12 (2410.98-3625.30)	72.71	215.87 (178.05-258.15)	210.24 (171.55-249.72)	-2.61
Mauritius	2021.57 (1665.86-2398.83)	2811.01 (2370.04-3359.28)	39.05	167.87 (138.69-199.71)	216.96 (182.50-261.78)	29.24
Bahrain	779.70 (629.80-949.35)	2564.30 (2093.44-3116.59)	228.88	135.48 (111.09-162.71)	133.69 (110.41-160.04)	-1.32

Lesotho	1638.01 (1346.39-2003.90)	2541.59 (2129.47-3011.89)	55.16	121.31 (101.79-144.45)	131.39 (110.07-153.69)	8.31
Trinidad and Tobago	2140.30 (1713.58-2680.30)	2523.29 (2108.40-2972.91)	17.89	171.98 (139.34-212.62)	182.30 (152.54-215.33)	6
Gabon	1057.16 (864.49-1277.17)	2277.40 (1890.97-2723.90)	115.43	122.80 (101.01-147.26)	127.43 (105.72-151.56)	3.77
The Gambia	767.72 (622.05-932.28)	2097.56 (1735.93-2504.38)	173.22	93.67 (76.36-111.99)	96.87 (80.20-114.47)	3.41
Timor-Leste	1092.09 (888.81-1329.59)	2069.71 (1667.50-2539.48)	89.52	142.06 (116.78-170.47)	145.75 (119.49-175.17)	2.6
Luxembourg	1159.20 (964.57-1363.61)	2034.64 (1718.29-2345.77)	75.52	299.39 (249.82-353.71)	322.82 (274.13-369.32)	7.83
Equatorial Guinea	383.68 (314.50-465.47)	1910.89 (1548.09-2292.41)	398.04	108.59 (89.70-129.30)	126.57 (103.58-150.29)	16.56
Guinea-Bissau	737.43 (598.38-889.28)	1735.98 (1444.87-2080.00)	135.41	90.34 (73.16-108.26)	94.62 (79.25-112.59)	4.74
Swaziland	922.73 (768.52-1105.57)	1710.71 (1447.37-1996.03)	85.4	130.48 (109.29-153.27)	144.23 (122.18-166.99)	10.54
Fiji	1382.48 (1102.37-1762.01)	1622.05 (1302.65-2010.89)	17.33	172.18 (139.86-214.90)	171.34 (137.98-212.06)	-0.48
Djibouti	418.82 (328.87-539.76)	1450.82 (1184.31-1785.66)	246.4	102.62 (82.54-126.07)	108.06 (88.85-132.39)	5.29
Guyana	1308.87 (1094.95-1564.15)	1299.38 (1059.52-1595.77)	-0.73	158.72 (132.37-187.17)	162.81 (132.57-200.57)	2.57
Solomon Islands	535.59 (414.63-699.33)	1170.71 (965.87-1400.72)	118.58	161.05 (131.33-196.93)	164.01 (136.37-192.72)	1.84
Brunei	640.94 (518.57-785.85)	1101.81 (899.49-1330.61)	71.91	226.59 (183.63-273.15)	211.91 (171.78-258.82)	-6.48
Suriname	721.02 (576.72-914.21)	1093.20 (904.53-1296.18)	51.62	179.08 (144.05-223.71)	185.34 (153.26-220.02)	3.49
Iceland	704.35 (590.82-844.28)	1074.86 (917.16-1254.47)	52.6	266.23 (223.11-318.38)	318.52 (272.44-368.13)	19.64
Montenegro	1150.33 (943.11-1365.99)	1045.89 (859.68-1242.63)	-9.08	176.89 (144.60-210.79)	171.14 (140.49-203.25)	-3.25
Bhutan	677.27 (554.24-834.57)	1038.90 (859.30-1233.40)	53.4	114.22 (93.42-136.44)	122.13 (101.57-144.83)	6.93
Maldives	302.84 (244.70-374.28)	1025.42 (830.91-1237.51)	238.6	154.56 (127.55-184.59)	161.31 (132.95-193.37)	4.36
Malta	823.67 (678.36-987.17)	1004.13 (827.43-1199.58)	21.91	214.91 (177.30-254.97)	256.21 (215.41-300.47)	19.21
The Bahamas	632.67 (519.07-757.26)	953.64 (788.14-1151.96)	50.73	222.10 (180.94-262.63)	226.10 (185.15-273.82)	1.8
Belize	397.18 (315.41-486.43)	931.22 (763.90-1138.02)	134.46	203.45 (167.48-242.93)	193.01 (159.35-233.68)	-5.13
Comoros	417.95 (327.15-538.32)	808.28 (650.53-984.61)	93.39	101.17 (80.59-123.32)	104.70 (85.23-126.14)	3.49
Cape Verde	276.51 (229.20-330.78)	733.45 (601.30-878.15)	165.25	99.86 (83.10-118.99)	119.78 (98.45-143.88)	19.95
Barbados	587.33 (466.17-723.33)	590.16 (489.32-695.86)	0.48	221.73 (175.74-274.84)	216.77 (179.54-261.00)	-2.24
Vanuatu	237.32 (186.19-304.86)	519.23 (432.17-612.92)	118.79	162.29 (131.47-199.09)	163.05 (136.59-189.71)	0.47

Samoa	332.07 (253.02-446.07)	470.54 (383.11-565.14)	41.7	189.72 (152.98-237.85)	214.83 (177.66-253.30)	13.23
Saint Lucia	359.77 (278.88-456.75)	370.92 (300.50-456.46)	3.1	238.46 (190.50-292.09)	207.61 (164.81-266.34)	-12.94
Guam	286.12 (231.11-351.42)	313.55 (255.59-378.06)	9.59	191.83 (154.79-236.96)	203.01 (162.81-246.88)	5.83
Andorra	166.64 (134.02-199.96)	235.03 (196.04-286.14)	41.04	265.90 (212.56-323.28)	280.53 (233.30-340.67)	5.5
Saint Vincent and the Grenadines	213.09 (164.87-282.76)	228.70 (186.42-278.75)	7.32	186.78 (149.59-235.70)	201.05 (161.57-247.97)	7.64
Kiribati	125.29 (99.86-158.15)	218.85 (179.75-262.24)	74.68	167.29 (135.00-207.12)	172.29 (142.90-203.84)	2.99
Grenada	173.01 (135.48-221.85)	217.95 (177.98-263.53)	25.98	203.70 (163.57-250.98)	204.43 (164.27-251.35)	0.36
Sao Tome and Principe	90.02 (73.53-108.88)	211.67 (173.79-254.09)	135.15	93.79 (76.66-113.40)	98.98 (82.43-118.30)	5.53
Federated States of Micronesia	182.27 (140.85-236.19)	199.96 (161.03-248.11)	9.7	179.51 (144.98-219.04)	180.43 (146.99-218.92)	0.51
Tonga	170.14 (131.81-220.76)	194.88 (158.66-235.60)	14.54	174.83 (141.52-214.25)	185.95 (153.23-220.06)	6.36
Seychelles	153.62 (124.09-188.57)	193.44 (158.40-228.99)	25.92	198.26 (161.51-237.47)	181.25 (148.63-216.36)	-8.58
Antigua and Barbuda	126.17 (101.24-156.96)	184.60 (150.09-223.26)	46.32	200.60 (161.05-246.83)	197.93 (158.70-246.37)	-1.33
Virgin Islands, U.S.	251.10 (203.34-312.18)	180.63 (152.26-216.53)	-28.06	224.93 (183.04-277.29)	240.24 (200.93-295.40)	6.8
Greenland	189.84 (161.90-223.12)	160.41 (136.74-186.53)	-15.51	318.93 (271.26-370.79)	304.91 (258.27-358.25)	-4.4
Dominica	183.64 (140.38-240.01)	154.11 (122.74-189.07)	-16.08	234.58 (185.89-296.67)	223.20 (176.99-275.55)	-4.85
Bermuda	132.78 (108.01-159.68)	122.61 (101.17-148.66)	-7.66	208.01 (167.03-259.52)	215.49 (172.51-268.04)	3.6
Saint Kitts and Nevis	78.73 (61.98-102.19)	121.96 (100.86-146.34)	54.91	189.92 (152.72-238.11)	196.53 (158.69-244.63)	3.48
Marshall Islands	79.34 (61.66-103.47)	116.36 (94.23-141.97)	46.65	185.37 (150.81-227.45)	190.74 (156.08-230.74)	2.9
American Samoa	91.86 (73.28-115.38)	98.53 (79.39-122.96)	7.26	187.48 (152.12-231.50)	193.53 (157.71-235.50)	3.23
Northern Mariana Islands	100.10 (82.26-120.63)	96.23 (78.99-116.90)	-3.87	186.07 (152.15-228.12)	196.20 (159.78-238.28)	5.44
Monaco	74.75 (61.48-89.56)	91.83 (76.52-107.69)	22.85	271.33 (218.70-329.87)	296.40 (245.67-356.50)	9.24
San Marino	65.04 (53.16-78.81)	82.99 (70.37-97.62)	27.61	261.81 (212.24-318.17)	285.13 (236.89-345.93)	8.91
Palau	31.20 (25.17-38.42)	34.76 (28.49-41.58)	11.44	184.23 (149.70-224.58)	190.07 (153.67-231.77)	3.17
Cook Islands	36.74 (29.30-46.03)	33.11 (26.67-40.45)	-9.88	188.31 (152.63-228.93)	194.23 (156.94-237.98)	3.14
Tuvalu	15.60 (12.51-19.23)	22.66 (18.26-27.90)	45.29	170.92 (138.16-209.98)	177.00 (143.43-215.99)	3.56
Nauru	18.28 (14.55-22.89)	21.17 (17.02-26.48)	15.85	181.49 (147.89-223.74)	181.57 (148.66-222.26)	0.04

Niue	4.04 (3.26-5.09)	3.12 (2.55-3.82)	-22.81	184.90 (150.34-227.65)	190.42 (154.29-231.56)	2.99
Tokelau	2.66 (2.11-3.39)	2.50 (2.01-3.10)	-5.9	177.68 (144.03-219.21)	182.31 (147.82-222.16)	2.6

Table S7. Prevalence, ASPRs and their changes in drug use disorders across 204 countries and territories from 1990 to 2021

Country	Number of prevalent cases			ASPR (per 100,000 population)		
	1990	2021	Change (%)	1990	2021	Change (%)
	Both (95%UI)	Both (95%UI)		Both (95%UI)	Both (95%UI)	
United States	5229953.07 (4489807.60-6133671.73)	12146953.91 (11024582.17-13461043.90)	132.26	1982.29 (1680.83-2333.75)	3821.43 (3450.13-4257.62)	92.78
China	11405507.33 (9702397.10-13528164.49)	7680058.66 (6602083.42-9057281.31)	-32.66	818.06 (704.13-963.15)	587.42 (492.04-702.02)	-28.19
India	3245133.09 (2598064.39-4199687.43)	6366009.45 (5297783.08-7997066.80)	96.17	391.83 (317.95-495.67)	409.32 (342.38-508.49)	4.46
Brazil	1458721.84 (1169620.07-1837393.81)	2062463.23 (1758127.35-2464972.61)	41.39	914.20 (749.97-1136.48)	902.83 (762.63-1084.49)	-1.24
Russian Federation	1660375.66 (1442753.22-1897251.56)	1470066.13 (1308204.85-1665674.47)	-11.46	1085.60 (935.54-1259.17)	1134.93 (984.21-1318.26)	4.54
Indonesia	834551.86 (660058.12-1058052.52)	1372610.97 (1100428.10-1725627.88)	64.47	421.62 (338.44-523.85)	452.25 (361.35-571.11)	7.27
United Kingdom	896052.59 (747401.89-1088071.24)	1179402.90 (1027623.69-1373691.70)	31.62	1590.12 (1303.65-1956.39)	1921.57 (1641.18-2286.92)	20.84
Pakistan	366547.36 (295813.83-465707.94)	858878.62 (695921.45-1092073.60)	134.32	376.42 (311.85-466.91)	356.82 (294.55-442.91)	-5.21
Canada	602319.65 (551908.81-664893.37)	770066.78 (726828.19-825876.99)	27.85	2148.62 (1971.06-2358.08)	2301.74 (2162.55-2471.77)	7.13
Japan	996761.50 (838198.75-1243077.48)	766692.19 (655299.05-935827.90)	-23.08	791.60 (657.46-1004.70)	765.43 (633.45-966.83)	-3.31
Germany	674023.24 (612958.66-754136.89)	733657.36 (677888.67-802161.59)	8.85	846.08 (759.60-949.73)	1014.39 (932.06-1119.29)	19.89
Mexico	430622.44 (357540.74-526527.32)	654887.44 (562929.50-768530.68)	52.08	493.14 (419.53-589.45)	477.21 (410.39-560.53)	-3.23
Iran	274874.39 (238602.54-317190.50)	645137.66 (576918.21-723726.43)	134.7	536.78 (477.31-606.02)	711.21 (632.66-809.46)	32.5
Philippines	344916.32 (272112.96-433671.06)	635510.70 (507362.18-794918.59)	84.25	527.38 (424.49-654.32)	514.86 (415.67-640.34)	-2.37
Vietnam	400232.69 (307702.34-520498.91)	627857.37 (517495.36-762563.45)	56.87	555.28 (441.49-706.78)	623.39 (505.54-767.38)	12.27
France	574146.24 (498959.81-663338.19)	620086.39 (548863.23-706616.10)	8	997.36 (864.12-1152.77)	1065.84 (927.89-1234.67)	6.87
Spain	560168.52 (521093.05-608111.84)	555555.37 (517631.78-601557.68)	-0.82	1392.34 (1294.70-1511.59)	1509.57 (1402.90-1644.83)	8.42
Bangladesh	296782.36 (241058.24-383752.45)	506095.74 (434235.59-611693.74)	70.53	300.11 (252.84-375.37)	288.16 (248.90-345.71)	-3.98
Nigeria	181960.24 (148503.51-227004.89)	469689.51 (386896.26-580185.85)	158.13	223.51 (187.74-267.69)	224.12 (190.37-268.09)	0.28

Thailand	482708.17 (382323.43-604468.35)	460438.23 (382538.65-559715.45)	-4.61	721.53 (578.78-891.68)	750.63 (610.67-928.25)	4.03
Italy	746133.82 (598929.10-933961.42)	456505.35 (386016.69-537179.21)	-38.82	1280.98 (1018.97-1613.02)	946.78 (769.67-1152.06)	-26.09
Colombia	205030.67 (175466.13-240187.51)	446866.58 (391312.89-511591.75)	117.95	583.52 (506.16-673.87)	845.97 (741.05-966.56)	44.98
South Africa	284333.30 (247278.35-334534.72)	440362.09 (371007.60-525633.66)	54.88	754.49 (669.73-866.42)	718.61 (606.30-864.41)	-4.76
Australia	417291.25 (374656.15-471268.17)	437073.73 (396829.03-484944.80)	4.74	2355.60 (2117.35-2664.52)	1802.46 (1626.25-2011.99)	-23.48
South Korea	435656.92 (342991.54-575774.03)	389462.34 (324940.92-494569.23)	-10.6	816.48 (651.18-1070.73)	815.83 (647.87-1065.77)	-0.08
Egypt	175458.61 (147446.03-209084.62)	388748.39 (336807.55-454042.11)	121.56	326.59 (279.67-380.69)	366.82 (320.42-425.01)	12.32
Ethiopia	143597.62 (108688.55-192450.04)	361435.04 (273524.46-486000.27)	151.7	319.98 (255.75-411.38)	319.47 (254.79-412.43)	-0.16
Argentina	219206.52 (188485.41-261059.43)	328135.13 (288897.04-378666.16)	49.69	676.88 (582.77-805.86)	696.67 (613.84-806.06)	2.92
Myanmar	252487.16 (193313.38-320918.25)	326029.68 (250922.18-419278.64)	29.13	570.23 (448.80-711.77)	545.68 (422.07-698.54)	-4.3
Ukraine	372497.97 (323293.10-423216.26)	325738.48 (291607.28-363905.64)	-12.55	705.21 (603.60-814.02)	821.95 (722.69-942.02)	16.55
Turkey	194778.45 (163433.54-231285.98)	270131.41 (232816.77-314827.17)	38.69	329.75 (280.35-388.39)	307.48 (263.71-359.97)	-6.75
Democratic Republic of the Congo	102456.05 (77787.00-138951.89)	267861.06 (205237.18-362503.46)	161.44	294.80 (234.46-383.22)	305.21 (244.63-397.51)	3.53
Poland	296060.35 (231654.92-371966.79)	250415.19 (207093.92-302939.20)	-15.42	809.57 (628.07-1022.06)	773.18 (616.46-958.76)	-4.49
Tanzania	98953.63 (74208.89-128043.85)	209212.12 (153021.05-284990.83)	111.42	394.83 (313.56-491.70)	359.46 (278.81-468.31)	-8.96
Chile	116739.10 (103014.12-132749.66)	206182.66 (188170.91-227209.13)	76.62	793.93 (701.52-896.85)	1099.00 (1005.10-1214.81)	38.43
Algeria	89793.35 (71753.41-111819.20)	182535.73 (158929.79-212194.56)	103.28	363.19 (301.79-437.48)	412.61 (357.98-483.00)	13.61
Saudi Arabia	60761.26 (49163.28-74082.81)	178424.00 (150816.58-211054.68)	193.65	377.01 (312.57-451.48)	379.06 (317.91-453.07)	0.55
Uzbekistan	107170.84 (86244.76-133343.09)	176224.02 (146092.63-213680.66)	64.43	507.84 (419.99-617.91)	494.98 (407.21-605.90)	-2.53
Malaysia	94535.08 (74534.57-119523.17)	175844.60 (145949.40-213339.39)	86.01	502.95 (403.80-621.94)	486.79 (404.32-589.39)	-3.21
Peru	99490.96 (82647.12-121161.23)	172028.88 (146515.69-209898.42)	72.91	447.01 (379.33-541.04)	440.26 (374.79-534.38)	-1.51
Iraq	62824.42 (50218.51-77508.36)	168864.24 (143604.60-197692.20)	168.79	361.61 (304.72-433.29)	380.66 (326.26-442.17)	5.27
Taiwan (Province of China)	142341.52 (117255.97-172564.17)	168579.99 (146692.29-193322.75)	18.43	605.85 (501.75-729.07)	733.88 (625.72-856.91)	21.13
Sudan	62677.23 (50840.97-77524.17)	164246.68 (137173.37-200408.47)	162.05	336.10 (283.49-402.18)	363.95 (311.37-430.24)	8.29
North Korea	129437.60 (106392.76-158131.75)	163431.75 (137717.09-197344.41)	26.26	579.71 (479.75-704.42)	584.94 (487.33-715.57)	0.9
Uganda	66067.89 (46119.16-90438.45)	162927.53 (121544.49-212926.41)	146.61	394.17 (294.87-517.82)	369.31 (287.56-464.31)	-6.31

Morocco	97579.05 (79767.53-119554.60)	158992.44 (136979.18-183882.88)	62.94	381.11 (321.30-461.71)	409.28 (352.30-473.90)	7.39
Kazakhstan	107941.06 (91865.85-130001.67)	157731.10 (139744.44-176913.30)	46.13	630.71 (538.77-754.66)	833.74 (731.36-942.99)	32.19
Netherlands	175126.83 (156360.70-197736.07)	149369.09 (131406.42-170694.78)	-14.71	1118.96 (994.92-1266.28)	973.31 (849.00-1122.65)	-13.02
Kenya	45426.20 (36560.15-57778.97)	138361.50 (108806.53-179516.57)	204.59	225.70 (190.41-275.44)	263.63 (216.20-330.66)	16.8
Afghanistan	34588.09 (28566.36-41744.71)	128820.74 (106784.08-153042.53)	272.44	432.23 (372.30-506.15)	436.29 (377.02-504.63)	0.94
Sri Lanka	109345.19 (86005.74-138156.12)	121091.41 (97216.09-153539.57)	10.74	580.78 (464.55-727.39)	550.95 (438.74-704.32)	-5.14
Yemen	35695.28 (28532.64-44611.96)	112497.85 (92909.72-137998.16)	215.16	321.78 (267.71-387.83)	341.47 (289.48-407.20)	6.12
Nepal	59512.23 (46883.08-80596.14)	110312.75 (87451.06-148127.36)	85.36	337.71 (273.56-441.36)	328.41 (264.26-430.19)	-2.76
Switzerland	125884.09 (107626.85-149859.19)	102145.24 (89324.46-118005.29)	-18.86	1827.70 (1537.91-2197.76)	1339.34 (1142.29-1583.10)	-26.72
Haiti	39711.74 (30141.40-53685.95)	100624.85 (74635.40-132968.73)	153.39	644.56 (509.84-842.42)	722.53 (545.49-940.78)	12.1
Sweden	51810.07 (44112.86-63181.06)	98603.87 (89010.34-110512.05)	90.32	624.62 (527.27-773.59)	1048.72 (938.15-1188.21)	67.9
Mozambique	37220.25 (27034.06-51586.76)	98510.03 (71349.41-136466.09)	164.67	310.15 (238.48-414.97)	331.80 (257.71-435.43)	6.98
Belgium	93046.61 (79821.33-109767.97)	97918.90 (88060.11-110541.03)	5.24	951.33 (803.40-1132.83)	978.14 (867.98-1115.46)	2.82
Venezuela	79890.61 (65737.22-100941.58)	97909.52 (82724.67-120425.04)	22.55	410.07 (342.16-503.07)	378.82 (318.76-475.33)	-7.62
Ecuador	52609.34 (42572.69-65439.19)	96858.87 (79467.52-118296.52)	84.11	508.31 (422.07-621.09)	506.32 (416.41-616.28)	-0.39
Ghana	34873.00 (27680.90-44821.83)	94630.06 (77685.18-117234.36)	171.36	256.33 (210.60-316.63)	272.82 (228.99-328.83)	6.43
Madagascar	35783.77 (25820.38-50090.04)	93348.47 (67516.12-129175.13)	160.87	317.62 (242.13-423.86)	317.78 (245.21-421.97)	0.05
New Zealand	59090.34 (48548.47-73247.64)	93143.02 (78245.01-114675.95)	57.63	1628.79 (1336.95-2019.89)	1885.74 (1553.63-2353.97)	15.78
Czech Republic	95173.50 (78953.44-112769.09)	91704.09 (79338.62-105746.54)	-3.65	968.85 (800.20-1149.69)	1148.67 (982.79-1356.41)	18.56
Angola	28677.18 (22087.27-38598.68)	90441.39 (69459.08-121554.86)	215.38	303.79 (243.52-394.47)	307.02 (248.46-396.20)	1.06
Cambodia	48231.33 (36994.79-62363.21)	86266.14 (69652.92-108816.64)	78.86	486.90 (384.33-612.09)	464.99 (377.89-582.90)	-4.5
Austria	72592.88 (62890.16-83951.08)	78085.61 (70516.41-87230.59)	7.57	883.41 (759.68-1023.26)	973.47 (864.41-1104.80)	10.19
Cuba	100225.08 (73888.36-135175.41)	77777.80 (61049.39-100575.31)	-22.4	779.25 (584.08-1038.18)	763.78 (572.27-1027.52)	-1.99
Cameroon	22717.36 (18064.79-29075.18)	77228.20 (61925.02-98631.07)	239.95	251.14 (206.43-308.00)	256.58 (213.05-314.68)	2.16
Papua New Guinea	28744.56 (20329.95-39955.85)	74112.29 (53711.95-101552.76)	157.83	665.00 (488.78-896.88)	667.88 (495.08-897.07)	0.43
Dominican Republic	39070.30 (32098.62-49190.56)	72927.12 (56433.54-96774.89)	86.66	513.36 (424.30-636.46)	615.46 (477.09-814.91)	19.89

Bolivia	32810.49 (28009.95-38998.57)	71107.53 (59678.43-85285.19)	116.72	534.29 (462.51-625.86)	562.23 (472.82-671.95)	5.23
United Arab Emirates	9689.22 (8405.50-11374.81)	70482.66 (63139.19-78290.77)	627.43	430.40 (372.13-503.94)	599.59 (536.70-671.51)	39.31
Somalia	23023.82 (16496.59-32161.22)	66059.62 (46874.11-92915.22)	186.92	320.48 (243.72-428.87)	316.68 (240.73-422.43)	-1.19
Cote d'Ivoire	27110.10 (21647.85-35081.87)	65179.25 (52783.55-83622.63)	140.42	243.87 (200.59-301.42)	244.00 (200.57-302.78)	0.05
Malawi	29908.82 (21555.79-41617.32)	64974.73 (46538.27-90247.25)	117.24	319.89 (244.93-426.20)	319.68 (244.92-422.81)	-0.07
Guatemala	24155.20 (20649.42-28486.48)	64711.26 (53168.85-81209.64)	167.9	345.28 (301.41-398.45)	377.55 (316.46-461.64)	9.35
Zimbabwe	40581.82 (31378.04-53170.76)	63705.56 (50276.25-81843.85)	56.98	439.28 (360.91-542.25)	423.42 (348.87-524.10)	-3.61
Israel	35666.47 (31745.20-40252.83)	63429.91 (54718.09-73715.72)	77.84	720.18 (642.89-811.22)	701.74 (604.28-821.04)	-2.56
Belarus	75692.27 (63401.74-89775.92)	60886.71 (53154.88-70028.72)	-19.56	720.06 (596.94-867.30)	717.84 (601.11-862.94)	-0.31
Portugal	81414.84 (69486.47-94370.27)	60624.38 (53711.00-68685.03)	-25.54	793.04 (675.54-922.80)	675.52 (585.66-780.98)	-14.82
Norway	38863.48 (33831.79-45438.92)	60463.39 (54378.54-67411.81)	55.58	891.16 (771.06-1050.46)	1141.64 (1019.61-1279.16)	28.11
Finland	45290.01 (40134.30-52711.83)	59742.94 (55190.45-65336.70)	31.91	895.13 (787.24-1053.37)	1210.96 (1111.41-1343.19)	35.28
Denmark	57549.02 (51071.87-65941.78)	59736.77 (54482.79-66955.89)	3.8	1092.98 (965.56-1256.59)	1094.63 (987.20-1242.67)	0.15
Rwanda	23400.57 (16563.38-32294.59)	58796.70 (41094.95-80720.59)	151.26	345.84 (258.10-462.04)	403.45 (298.37-534.71)	16.66
Ireland	41278.16 (35658.29-48211.69)	58373.33 (54076.63-64113.49)	41.41	1119.46 (966.52-1301.52)	1289.87 (1188.59-1421.35)	15.22
Greece	57651.16 (51536.93-64651.07)	58025.38 (52481.51-65035.00)	0.65	557.89 (499.18-627.67)	686.91 (614.77-786.78)	23.13
Zambia	28525.21 (22548.22-35151.80)	56593.84 (44694.50-73645.19)	98.4	366.68 (304.76-439.98)	292.84 (241.70-362.32)	-20.14
Azerbaijan	41914.74 (34207.17-51894.43)	55717.71 (47065.74-67098.87)	32.93	522.86 (433.43-637.02)	498.53 (412.32-613.01)	-4.65
Honduras	19359.57 (15619.01-24910.52)	55715.89 (43284.96-72678.43)	187.8	461.46 (386.02-571.49)	506.15 (406.43-646.85)	9.69
Romania	74188.46 (63109.90-89875.91)	54090.83 (48069.91-61022.50)	-27.09	316.48 (268.64-388.30)	320.98 (283.72-371.87)	1.42
Tajikistan	27189.84 (21782.79-34265.08)	52540.71 (43247.70-64966.96)	93.24	519.23 (428.37-636.74)	496.48 (411.57-610.07)	-4.38
Hungary	63935.06 (53919.48-76177.25)	52472.34 (45526.68-60623.33)	-17.93	656.55 (548.76-790.49)	660.61 (561.74-772.12)	0.62
Jordan	13704.02 (10801.70-17055.92)	50165.02 (41238.84-60860.03)	266.06	367.17 (305.17-440.43)	366.25 (304.94-439.11)	-0.25
Niger	15840.89 (12486.67-20511.04)	49806.37 (38663.73-65802.93)	214.42	239.69 (196.55-297.58)	239.12 (196.41-297.12)	-0.24
Mali	17157.41 (13715.55-22146.24)	49377.13 (38417.85-65140.33)	187.79	236.49 (192.69-292.46)	234.42 (192.34-292.55)	-0.88
Syria	36636.80 (30097.01-44767.08)	47504.54 (39907.71-56305.76)	29.66	319.76 (272.21-380.80)	348.55 (299.27-407.97)	9

Tunisia	29930.04 (24515.15-36648.53)	46406.06 (40363.36-53737.04)	55.05	350.67 (295.04-420.47)	388.41 (333.71-454.44)	10.76
Burkina Faso	17318.36 (13775.48-22406.66)	45705.19 (37524.18-58204.92)	163.91	226.49 (185.77-279.01)	228.58 (193.64-278.67)	0.92
Singapore	31267.80 (24826.46-40906.51)	43060.16 (36164.55-53929.17)	37.71	815.62 (648.09-1069.61)	806.16 (640.05-1055.05)	-1.16
Burundi	16144.02 (11733.89-22302.17)	40887.22 (29583.98-56495.99)	153.27	316.00 (242.07-422.31)	316.56 (242.60-422.27)	0.18
Laos	19181.48 (14347.84-25312.28)	39764.24 (30777.08-52084.78)	107.31	484.54 (377.25-627.24)	484.31 (377.55-627.31)	-0.05
Bulgaria	48786.52 (42244.13-56586.65)	38207.00 (34158.16-42884.59)	-21.69	597.34 (513.68-698.03)	729.96 (644.92-825.51)	22.2
Kyrgyzstan	24936.78 (20510.44-30647.34)	37770.67 (31576.12-45571.88)	51.47	564.90 (475.98-680.29)	540.46 (452.72-653.44)	-4.33
Senegal	16233.48 (12856.96-20969.91)	37730.96 (30004.99-48631.75)	132.43	248.85 (204.67-305.98)	244.33 (201.58-302.05)	-1.81
Libya	14860.23 (12087.81-18500.98)	37559.39 (33228.38-42595.61)	152.75	369.35 (311.37-440.54)	470.18 (414.06-534.72)	27.3
Paraguay	18689.47 (15707.93-22169.68)	37308.21 (30750.89-45582.71)	99.62	476.70 (406.55-556.93)	473.89 (393.86-577.12)	-0.59
Serbia	44644.67 (35817.51-54549.96)	37091.24 (32901.73-42492.07)	-16.92	467.85 (370.03-575.87)	451.74 (393.65-526.25)	-3.44
Benin	10435.17 (8106.88-13671.79)	35200.51 (27504.94-44959.83)	237.33	257.51 (208.34-324.65)	276.25 (226.93-342.12)	7.28
Chad	11984.33 (9467.60-15510.38)	34886.10 (27012.97-46134.39)	191.1	239.72 (196.33-297.45)	236.74 (193.72-293.29)	-1.24
Turkmenistan	19959.91 (16138.25-25036.98)	32232.92 (27295.06-38722.69)	61.49	531.27 (443.16-648.77)	592.31 (500.98-709.86)	11.49
Slovakia	37722.98 (31800.40-45282.69)	31387.72 (27965.72-35774.09)	-16.79	716.74 (603.42-862.34)	675.57 (596.84-777.03)	-5.74
Uruguay	29450.40 (24805.07-35136.41)	30841.59 (26606.34-35739.34)	4.72	961.19 (809.74-1149.79)	940.73 (808.17-1093.17)	-2.13
Lebanon	11605.61 (9696.77-13729.07)	30490.78 (26668.27-34654.52)	162.72	391.61 (332.22-457.70)	510.44 (443.21-584.33)	30.34
South Sudan	18840.82 (13464.09-26382.57)	29356.23 (20794.86-41191.81)	55.81	326.54 (249.70-434.29)	316.47 (242.89-421.66)	-3.09
Croatia	35292.45 (29458.62-43335.38)	28798.22 (25938.21-32993.59)	-18.4	743.45 (613.63-923.12)	815.28 (719.94-945.54)	9.66
Jamaica	22115.07 (16635.52-29084.71)	28367.12 (23006.49-35752.15)	28.27	859.33 (668.95-1093.14)	916.33 (740.32-1154.24)	6.63
Guinea	11710.15 (9421.85-14981.34)	28138.59 (22387.59-36841.92)	140.29	232.18 (189.95-287.94)	230.42 (188.35-287.97)	-0.76
Puerto Rico	37364.01 (29721.92-47605.81)	26995.44 (21729.27-33902.82)	-27.75	999.36 (800.21-1266.11)	900.01 (699.20-1159.31)	-9.94
Costa Rica	16400.22 (12883.10-21270.90)	26393.02 (22205.23-31638.09)	60.93	514.88 (415.24-654.77)	526.66 (443.47-630.81)	2.29
El Salvador	17582.44 (14885.08-20701.54)	26025.16 (21390.41-32717.86)	48.02	340.91 (292.56-397.62)	381.56 (316.05-474.27)	11.93
Nicaragua	14894.59 (12180.66-18177.70)	24647.69 (20813.82-29779.34)	65.48	408.79 (342.10-486.66)	341.49 (289.57-411.45)	-16.46
Lithuania	23545.76 (21106.94-26402.52)	23702.12 (22071.42-25589.87)	0.66	624.35 (555.56-703.84)	980.90 (903.30-1071.86)	57.11

Kuwait	8217.60 (6812.92-9962.29)	23368.54 (20250.42-27139.24)	184.37	401.32 (335.51-479.20)	433.49 (367.78-509.79)	8.02
Panama	16045.42 (12652.80-20543.83)	23357.36 (18532.90-30098.38)	45.57	613.48 (499.99-765.61)	529.25 (419.93-680.93)	-13.73
Palestine	7453.05 (5932.85-9264.33)	22870.54 (18157.10-27813.50)	206.86	396.70 (330.05-476.10)	411.37 (334.79-492.65)	3.7
Eritrea	10384.63 (7429.38-14462.18)	22829.90 (16872.26-31208.33)	119.84	319.06 (245.00-425.59)	327.27 (251.46-435.05)	2.57
Sierra Leone	9561.42 (7734.50-12114.77)	21738.31 (17338.41-27868.95)	127.35	250.97 (208.05-307.43)	246.01 (203.35-303.60)	-1.98
Estonia	15011.45 (13239.72-17006.29)	20606.11 (19323.50-21877.93)	37.27	986.68 (861.08-1130.14)	1865.24 (1738.46-1997.62)	89.04
Oman	7111.74 (5854.04-8692.02)	19804.58 (16815.19-23698.04)	178.48	358.34 (300.05-431.31)	353.23 (294.24-423.79)	-1.43
Moldova	28651.09 (24104.43-33597.08)	19736.08 (17283.40-22596.71)	-31.12	643.25 (540.70-758.71)	571.14 (488.93-666.05)	-11.21
Mongolia	12535.30 (9939.85-15849.34)	19667.96 (16416.20-23519.14)	56.9	568.63 (466.28-697.37)	607.13 (500.72-731.46)	6.77
Congo	6978.38 (5328.65-9476.91)	17353.66 (13713.77-22721.49)	148.68	306.29 (246.87-398.02)	314.73 (254.85-404.14)	2.76
Togo	6667.15 (5368.34-8611.38)	16919.15 (14418.11-20590.88)	153.77	209.41 (175.37-258.86)	209.86 (180.47-248.47)	0.22
Liberia	6020.64 (4753.15-7963.60)	16870.36 (12944.78-21706.46)	180.21	275.66 (223.65-349.54)	304.16 (243.23-378.18)	10.34
Central African Republic	7634.67 (5876.70-10280.50)	15954.40 (12259.31-21598.09)	108.97	299.01 (240.35-387.52)	297.14 (237.41-387.78)	-0.63
Armenia	20501.73 (17124.50-24608.21)	15689.60 (13362.85-18240.57)	-23.47	561.91 (472.36-674.14)	541.60 (453.28-641.97)	-3.61
Georgia	27476.33 (23473.73-32246.15)	15585.14 (13511.17-18089.70)	-43.28	481.16 (409.96-565.87)	475.72 (398.68-566.74)	-1.13
Albania	17725.93 (13633.34-22830.61)	14757.02 (12633.00-17594.11)	-16.75	485.52 (379.55-617.89)	553.14 (466.56-668.81)	13.93
Qatar	2079.06 (1740.89-2508.37)	14718.07 (12452.00-17534.96)	607.92	372.32 (311.42-448.26)	366.57 (307.99-442.44)	-1.54
Latvia	23362.50 (20570.68-26834.19)	14638.11 (13276.76-16333.86)	-37.34	895.85 (785.58-1037.64)	948.49 (848.28-1075.44)	5.88
Slovenia	16003.22 (12948.51-20006.14)	13557.72 (12158.33-15158.61)	-15.28	819.59 (653.10-1036.18)	854.36 (758.63-967.70)	4.24
Namibia	5904.58 (4671.93-7524.63)	12396.27 (9975.62-15131.32)	109.94	439.08 (362.33-537.78)	478.90 (394.91-574.85)	9.07
Bosnia and Herzegovina	19045.15 (14988.31-24230.19)	10652.08 (9091.37-12663.50)	-44.07	385.93 (302.33-493.90)	361.88 (299.58-444.88)	-6.23
Mauritania	4528.11 (3619.66-5817.17)	10105.87 (8002.37-13026.85)	123.18	247.94 (203.91-306.10)	247.54 (204.35-305.82)	-0.16
Botswana	4540.21 (3606.96-5760.39)	9983.40 (8447.31-11771.88)	119.89	371.89 (307.86-453.16)	383.49 (326.44-449.02)	3.12
Trinidad and Tobago	8855.50 (6679.61-11840.08)	9868.39 (8117.07-12025.39)	11.44	695.55 (532.48-914.25)	739.97 (593.43-917.16)	6.39
Macedonia	9545.06 (8115.05-11375.87)	9729.47 (8429.66-11400.23)	1.93	456.18 (387.69-543.31)	455.00 (388.67-539.12)	-0.26
Mauritius	8036.08 (6331.34-10207.67)	9468.54 (8154.96-11247.99)	17.83	640.99 (510.09-806.73)	739.08 (624.46-881.20)	15.3

Lesotho	5541.21 (4353.71-7099.10)	8808.53 (7008.97-11092.17)	58.96	412.39 (336.68-508.09)	439.94 (363.44-541.37)	6.68
Cyprus	5516.81 (4701.28-6711.90)	8582.73 (7648.87-9740.16)	55.57	677.87 (577.26-822.09)	633.29 (564.90-721.82)	-6.58
Timor-Leste	4348.24 (3361.10-5663.09)	7935.59 (5987.98-10452.27)	82.5	533.85 (418.10-683.64)	528.42 (415.25-680.57)	-1.02
Luxembourg	4305.32 (3644.22-5201.97)	6917.15 (6087.05-8074.31)	60.67	1097.45 (911.51-1359.50)	1137.93 (981.04-1339.42)	3.69
Bahrain	2277.85 (1887.84-2772.03)	6842.55 (5792.37-8131.86)	200.4	372.43 (314.34-447.14)	367.75 (308.13-439.50)	-1.26
Fiji	5556.64 (4038.21-7643.27)	6251.77 (4647.66-8385.62)	12.51	669.45 (496.66-896.19)	665.25 (494.97-892.52)	-0.63
Gabon	2978.37 (2322.23-3938.88)	5997.57 (4772.98-7814.91)	101.37	320.83 (261.38-411.13)	322.17 (262.96-411.51)	0.42
Swaziland	3160.69 (2539.76-3877.60)	5815.80 (4744.82-7005.47)	84	433.04 (361.80-514.52)	472.65 (397.46-561.18)	9.15
The Gambia	2209.01 (1754.34-2844.21)	5726.61 (4544.92-7430.58)	159.24	249.89 (205.41-305.87)	244.16 (200.99-304.45)	-2.29
Equatorial Guinea	1098.53 (847.20-1481.30)	5418.83 (4222.97-7334.37)	393.28	296.79 (239.74-385.78)	329.64 (266.15-426.57)	11.07
Guyana	5415.25 (4362.45-6866.77)	5332.73 (4041.40-7070.04)	-1.52	629.91 (520.70-782.95)	640.90 (492.76-843.69)	1.74
Solomon Islands	2166.07 (1541.16-2956.84)	4766.86 (3669.74-6058.28)	120.07	639.95 (483.13-848.45)	662.02 (518.74-828.67)	3.45
Guinea-Bissau	2041.13 (1596.56-2666.93)	4637.78 (3677.83-6068.33)	127.22	233.64 (191.40-289.44)	234.65 (191.59-292.81)	0.43
Djibouti	1404.20 (996.97-1963.61)	4503.38 (3417.86-6050.84)	220.71	320.22 (243.34-428.74)	333.26 (256.06-442.79)	4.07
Brunei	2624.45 (2078.27-3443.24)	4311.89 (3486.49-5580.19)	64.3	889.96 (716.34-1148.06)	822.11 (657.03-1071.76)	-7.62
Belize	1800.59 (1430.90-2258.05)	4202.11 (3188.07-5572.70)	133.37	917.44 (752.57-1132.28)	848.63 (656.16-1105.52)	-7.5
Suriname	2972.51 (2177.35-4034.87)	4089.43 (3324.37-5012.75)	37.57	695.97 (531.54-918.32)	700.88 (566.25-864.34)	0.71
Iceland	2429.00 (2090.56-2857.64)	4052.10 (3707.64-4507.63)	66.82	899.92 (774.34-1058.84)	1191.09 (1080.70-1329.81)	32.35
The Bahamas	2800.87 (2201.83-3616.31)	3781.46 (2933.73-4929.29)	35.01	928.01 (744.47-1175.59)	906.76 (699.71-1190.59)	-2.29
Maldives	1127.13 (841.32-1489.20)	3495.52 (2826.00-4408.00)	210.13	532.10 (411.70-684.23)	560.04 (439.60-719.28)	5.25
Malta	2457.39 (2145.74-2825.74)	3281.08 (2933.12-3742.56)	33.52	666.96 (577.80-775.24)	866.47 (762.34-1004.03)	29.91
Bhutan	2062.64 (1586.93-2899.15)	2914.03 (2341.13-3888.03)	41.28	331.95 (266.40-437.63)	334.23 (269.64-439.74)	0.68
Barbados	2835.33 (2129.59-3774.42)	2770.19 (2249.88-3421.12)	-2.3	1024.39 (773.12-1352.91)	1014.99 (804.17-1272.67)	-0.92
Montenegro	3352.36 (2678.43-4099.13)	2652.75 (2247.09-3182.21)	-20.87	510.77 (407.22-626.04)	459.00 (382.29-561.69)	-10.14
Comoros	1369.73 (970.25-1917.09)	2542.43 (1902.23-3438.58)	85.62	312.09 (236.81-415.25)	320.16 (245.24-424.96)	2.59
Samoa	1417.45 (964.49-2035.15)	2063.39 (1608.42-2626.74)	45.57	789.24 (577.57-1078.73)	942.75 (747.44-1184.43)	19.45

Vanuatu	920.84 (669.11-1272.37)	1993.48 (1564.62-2477.25)	116.48	610.10 (460.22-817.76)	609.55 (488.68-753.38)	-0.09
Saint Lucia	1762.32 (1319.61-2337.13)	1661.07 (1256.25-2227.56)	-5.75	1147.16 (888.27-1473.46)	923.04 (674.83-1272.98)	-19.54
Cape Verde	778.52 (613.80-1004.81)	1626.17 (1351.45-1991.09)	108.88	249.43 (206.05-307.43)	259.88 (217.49-316.96)	4.19
Guam	1125.60 (840.50-1514.65)	1088.27 (836.15-1422.96)	-3.32	699.58 (526.91-933.90)	707.48 (536.61-938.09)	1.13
Saint Vincent and the Grenadines	915.18 (649.36-1293.23)	967.84 (747.74-1242.94)	5.75	771.38 (580.57-1052.07)	852.90 (647.61-1103.99)	10.57
Grenada	750.13 (552.77-996.81)	952.02 (738.18-1244.96)	26.91	860.96 (654.63-1111.75)	861.75 (662.52-1124.36)	0.09
Greenland	1151.49 (998.03-1330.20)	910.27 (790.49-1059.06)	-20.95	1740.03 (1497.75-2011.88)	1650.50 (1418.76-1935.74)	-5.15
Kiribati	525.10 (383.81-706.07)	895.25 (686.17-1150.48)	70.49	663.09 (502.59-874.84)	695.31 (542.39-883.73)	4.86
Federated States of Micronesia	701.27 (498.40-966.93)	785.15 (576.30-1065.02)	11.96	686.72 (513.46-912.65)	693.96 (520.85-923.67)	1.05
Tonga	674.89 (478.01-937.75)	766.52 (579.49-1007.89)	13.58	678.04 (511.69-891.47)	725.42 (557.86-934.73)	6.99
Seychelles	678.58 (512.48-877.46)	754.67 (596.57-943.97)	11.21	823.52 (635.04-1057.64)	733.84 (567.50-935.57)	-10.89
Antigua and Barbuda	553.43 (410.18-725.86)	740.95 (575.13-973.06)	33.88	838.37 (631.88-1083.05)	795.96 (602.59-1059.36)	-5.06
Dominica	898.02 (644.22-1215.30)	719.21 (549.41-936.00)	-19.91	1126.51 (829.16-1493.33)	1033.37 (786.01-1352.52)	-8.27
Andorra	548.93 (438.40-689.50)	675.72 (562.08-824.35)	23.1	868.36 (682.85-1117.18)	888.79 (706.00-1134.18)	2.35
Virgin Islands, U.S.	892.40 (684.81-1179.08)	604.23 (487.54-760.58)	-32.29	833.39 (642.79-1091.47)	856.05 (663.11-1119.36)	2.72
Sao Tome and Principe	259.37 (206.06-336.28)	565.27 (456.35-712.82)	117.94	253.00 (210.31-311.19)	257.10 (213.80-315.06)	1.62
Saint Kitts and Nevis	336.68 (244.18-465.57)	480.67 (373.43-625.44)	42.76	772.09 (581.09-1032.86)	780.82 (588.31-1037.90)	1.13
Bermuda	529.40 (413.53-680.55)	435.64 (355.18-544.64)	-17.71	815.29 (621.09-1076.43)	826.27 (632.78-1092.42)	1.35
Marshall Islands	299.15 (211.02-418.47)	425.62 (316.75-576.57)	42.28	682.95 (510.32-916.06)	693.05 (525.07-923.68)	1.48
Northern Mariana Islands	414.74 (319.31-544.33)	367.27 (287.89-466.91)	-11.44	724.02 (557.06-948.06)	795.83 (614.25-1020.35)	9.92
American Samoa	355.84 (257.07-491.49)	332.92 (247.71-448.46)	-6.44	680.75 (507.79-908.64)	687.45 (517.15-920.68)	0.98
Monaco	240.76 (200.80-292.03)	271.39 (228.01-328.54)	12.72	900.11 (716.98-1146.91)	927.55 (742.78-1175.77)	3.05
San Marino	226.03 (180.86-285.06)	250.75 (208.27-307.16)	10.94	888.01 (702.24-1127.61)	909.72 (723.66-1148.92)	2.44
Palau	122.88 (90.59-167.42)	120.59 (94.03-154.92)	-1.86	693.35 (521.17-925.20)	714.15 (538.55-951.05)	3
Cook Islands	138.02 (99.34-189.69)	112.17 (85.57-147.75)	-18.73	681.13 (505.04-913.92)	668.60 (506.26-889.06)	-1.84
Tuvalu	62.54 (46.31-85.06)	90.44 (66.66-123.20)	44.6	668.50 (499.49-899.09)	688.78 (515.83-922.01)	3.03

Nauru	70.53 (51.19-97.22)	82.55 (60.43-113.49)	17.04	682.30 (508.67-910.80)	689.09 (519.91-918.85)	0.99
Niue	14.99 (11.00-20.33)	11.05 (8.34-14.75)	-26.29	690.01 (517.52-923.80)	692.55 (520.42-925.53)	0.37
Tokelau	10.32 (7.52-14.20)	9.21 (6.84-12.42)	-10.76	676.71 (507.25-907.27)	682.87 (510.23-912.07)	0.91

Table S8. Deaths, ASMRs, and their changes in drug use disorders across 204 countries and territories from 1990 to 2021

	Number of deaths			ASMR (per 100,000 population)		
	1990	2021	Change (%)	1990	2021	Change (%)
	Both (95%UI)	Both (95%UI)		Both (95%UI)	Both (95%UI)	
United States	5713.86 (5382.45-6055.93)	70893.11 (64047.09-78963.69)	1140.72	2.00 (1.88-2.11)	19.52 (17.73-21.61)	878.01
China	31334.42 (27329.88-35980.94)	11482.75 (9277.95-13907.42)	-63.35	2.75 (2.40-3.15)	0.69 (0.56-0.83)	-74.89
India	3667.59 (3211.05-4223.24)	8782.43 (7643.31-9958.81)	139.46	0.61 (0.53-0.69)	0.66 (0.57-0.74)	8.74
Russian Federation	4396.29 (4110.93-4697.76)	6557.22 (5977.11-7150.94)	49.15	2.66 (2.49-2.85)	4.07 (3.71-4.43)	52.85
United Kingdom	786.74 (768.35-805.98)	3795.42 (3663.26-3949.35)	382.42	1.30 (1.27-1.33)	5.21 (5.02-5.41)	299.56
Canada	409.59 (375.79-444.29)	3555.01 (3256.20-3864.31)	767.94	1.31 (1.21-1.42)	8.85 (8.08-9.60)	574.09
Iran	1687.25 (1450.46-2012.45)	2999.47 (2656.57-3340.41)	77.77	4.14 (3.59-4.90)	3.13 (2.76-3.47)	-24.38
France	542.25 (501.94-581.96)	1970.45 (1720.71-2198.53)	263.38	0.83 (0.77-0.90)	1.93 (1.72-2.14)	132.74
Germany	1152.85 (1079.24-1236.22)	1887.69 (1698.99-2080.85)	63.74	1.30 (1.22-1.40)	1.87 (1.67-2.09)	43.99
Australia	450.24 (418.57-483.42)	1416.07 (1259.29-1571.49)	214.51	2.47 (2.30-2.65)	4.91 (4.37-5.46)	98.91
Brazil	128.70 (121.72-135.89)	1404.56 (1314.43-1515.85)	991.34	0.09 (0.09-0.10)	0.59 (0.55-0.63)	527.7
Vietnam	470.95 (351.63-643.39)	1223.18 (887.10-1671.94)	159.72	0.86 (0.65-1.14)	1.21 (0.90-1.63)	40.7
Pakistan	378.75 (276.53-458.03)	1104.41 (857.61-1431.12)	191.59	0.54 (0.39-0.65)	0.66 (0.52-0.85)	23.06
Ukraine	1119.44 (980.04-1273.32)	856.69 (607.71-1150.28)	-23.47	1.83 (1.60-2.09)	1.70 (1.19-2.31)	-7.09
South Africa	454.78 (387.98-513.05)	814.14 (731.22-905.54)	79.02	1.52 (1.28-1.71)	1.49 (1.34-1.65)	-2.28
Spain	597.59 (540.55-663.72)	792.90 (700.31-878.92)	32.68	1.48 (1.33-1.65)	1.31 (1.17-1.46)	-11.17
Bangladesh	324.10 (199.00-441.77)	782.62 (405.88-1121.96)	141.48	0.49 (0.30-0.65)	0.52 (0.27-0.73)	5.82
Algeria	212.36 (158.09-281.84)	692.83 (472.30-931.43)	226.25	1.21 (0.90-1.58)	1.61 (1.12-2.13)	33.25

Morocco	218.50 (166.46-280.46)	602.63 (432.37-863.80)	175.81	1.10 (0.84-1.40)	1.59 (1.15-2.26)	45.06
Iraq	157.25 (120.24-208.22)	600.76 (430.66-885.86)	282.03	1.25 (0.98-1.65)	1.65 (1.21-2.36)	31.97
Mexico	246.81 (235.96-259.29)	579.79 (507.01-660.28)	134.91	0.33 (0.32-0.35)	0.42 (0.37-0.48)	27.85
Sudan	161.29 (117.33-226.12)	579.49 (385.69-817.06)	259.28	1.16 (0.86-1.60)	1.64 (1.12-2.30)	40.99
Indonesia	126.15 (108.40-145.65)	477.78 (394.36-594.78)	278.73	0.10 (0.09-0.12)	0.20 (0.17-0.25)	97.37
Kazakhstan	163.60 (128.64-206.70)	455.04 (342.80-574.14)	178.15	1.07 (0.84-1.35)	2.34 (1.78-2.95)	119.1
Tanzania	129.63 (72.23-209.84)	444.49 (244.09-684.71)	242.88	0.93 (0.53-1.53)	1.08 (0.62-1.63)	16.14
Italy	1051.84 (988.46-1120.79)	442.53 (401.32-481.89)	-57.93	1.76 (1.65-1.87)	0.59 (0.55-0.64)	-66.3
Afghanistan	117.39 (86.24-159.23)	423.03 (311.59-578.62)	260.35	1.67 (1.23-2.24)	2.03 (1.51-2.72)	21.75
Turkey	175.28 (140.89-216.29)	408.66 (323.34-508.33)	133.15	0.38 (0.31-0.46)	0.45 (0.36-0.56)	19.57
Sweden	90.78 (83.28-99.27)	398.71 (353.39-447.15)	339.19	1.01 (0.92-1.11)	3.69 (3.27-4.14)	265.06
Yemen	69.36 (41.14-108.63)	325.24 (203.79-499.10)	368.92	0.94 (0.60-1.38)	1.33 (0.88-2.01)	42.64
Taiwan (Province of China)	301.62 (278.08-324.52)	318.26 (281.07-365.42)	5.52	1.44 (1.32-1.55)	1.08 (0.96-1.23)	-24.46
Poland	254.55 (236.24-273.85)	309.51 (280.88-340.66)	21.59	0.64 (0.59-0.69)	0.73 (0.67-0.80)	14.76
Finland	149.30 (137.94-160.41)	306.03 (273.17-341.54)	104.99	2.60 (2.40-2.79)	5.06 (4.51-5.63)	94.21
Japan	110.08 (105.59-114.82)	297.83 (275.84-317.61)	170.56	0.08 (0.07-0.08)	0.18 (0.17-0.20)	139.51
Belarus	238.58 (162.61-373.46)	279.49 (212.75-365.58)	17.15	2.12 (1.45-3.32)	2.47 (1.87-3.22)	16.53
Denmark	187.31 (163.90-212.84)	265.04 (236.96-292.81)	41.5	3.25 (2.84-3.71)	3.83 (3.41-4.24)	17.95
Greece	79.47 (71.78-88.22)	264.04 (236.89-294.87)	232.24	0.72 (0.65-0.81)	2.28 (2.02-2.59)	215.29
Democratic Republic of the Congo	77.89 (43.48-118.76)	252.62 (133.15-414.26)	224.34	0.31 (0.17-0.47)	0.37 (0.20-0.61)	21.84
Norway	79.98 (74.45-86.10)	250.71 (233.73-270.79)	213.45	1.77 (1.65-1.91)	4.09 (3.80-4.43)	130.59
Belgium	78.28 (72.69-84.90)	242.05 (214.91-270.00)	209.2	0.70 (0.65-0.76)	1.81 (1.61-2.02)	158.21
Switzerland	330.62 (277.87-374.08)	230.50 (207.50-252.09)	-30.28	4.31 (3.62-4.89)	2.10 (1.90-2.30)	-51.32
Uganda	54.19 (27.11-91.75)	220.18 (132.35-334.77)	306.31	0.65 (0.33-1.07)	0.86 (0.52-1.27)	32.47
Libya	41.10 (32.11-51.94)	219.13 (151.31-301.26)	433.11	1.40 (1.10-1.76)	2.83 (1.98-3.86)	102.5
Guatemala	73.52 (67.59-80.15)	207.23 (171.39-248.28)	181.87	1.34 (1.23-1.46)	1.37 (1.13-1.66)	2.54

Tunisia	72.68 (50.54-92.34)	200.02 (136.86-284.17)	175.21	1.10 (0.77-1.38)	1.54 (1.07-2.16)	40.03
Netherlands	72.14 (64.83-80.19)	198.73 (179.26-221.52)	175.5	0.42 (0.38-0.47)	1.04 (0.93-1.17)	145
Ethiopia	82.31 (62.21-130.63)	196.72 (121.14-264.63)	138.98	0.29 (0.22-0.45)	0.26 (0.16-0.35)	-8.07
Romania	218.89 (167.46-276.51)	193.85 (159.39-226.96)	-11.44	0.89 (0.68-1.12)	0.83 (0.68-0.98)	-6.75
Austria	85.66 (78.60-92.51)	192.32 (175.17-209.57)	124.52	0.98 (0.90-1.06)	1.84 (1.66-2.03)	87.58
Ireland	41.15 (37.89-44.37)	190.61 (167.97-214.06)	363.25	1.15 (1.05-1.24)	3.59 (3.18-4.04)	212.72
North Korea	191.65 (134.69-269.94)	186.33 (136.68-265.54)	-2.77	0.95 (0.67-1.33)	0.60 (0.44-0.86)	-36.92
Peru	73.98 (60.34-88.78)	180.35 (136.62-231.63)	143.8	0.41 (0.33-0.48)	0.49 (0.37-0.63)	20.24
Syria	65.75 (51.33-82.74)	156.03 (117.19-212.45)	137.32	0.80 (0.64-1.00)	1.18 (0.90-1.58)	47.87
Zambia	37.66 (24.46-55.85)	155.31 (95.63-241.62)	312.42	0.94 (0.61-1.43)	1.15 (0.71-1.74)	21.56
Colombia	105.12 (96.60-114.52)	153.29 (125.04-183.34)	45.82	0.34 (0.32-0.37)	0.28 (0.23-0.34)	-17.26
Kenya	43.98 (25.76-55.91)	152.46 (101.73-197.65)	246.64	0.46 (0.27-0.59)	0.51 (0.36-0.64)	10.41
Philippines	59.01 (53.08-66.04)	148.02 (123.50-173.82)	150.85	0.14 (0.12-0.15)	0.15 (0.13-0.18)	10.24
Madagascar	48.49 (30.89-71.42)	146.52 (87.14-219.82)	202.15	0.72 (0.45-1.03)	0.76 (0.45-1.13)	5.2
Thailand	56.64 (46.99-66.59)	146.49 (110.51-189.00)	158.62	0.12 (0.10-0.14)	0.18 (0.13-0.23)	52.11
Sri Lanka	158.38 (137.66-184.00)	142.13 (95.96-190.94)	-10.26	1.05 (0.92-1.21)	0.58 (0.39-0.77)	-45.05
Uzbekistan	35.49 (30.50-41.05)	137.20 (108.61-170.52)	286.59	0.22 (0.19-0.25)	0.41 (0.33-0.50)	87.18
Portugal	76.28 (67.81-85.70)	131.32 (118.70-145.85)	72.15	0.74 (0.65-0.83)	1.02 (0.91-1.14)	38.57
Nepal	50.19 (35.58-66.28)	129.77 (88.98-176.51)	158.55	0.41 (0.29-0.53)	0.49 (0.34-0.66)	19.46
Malawi	38.76 (21.49-62.54)	124.06 (70.28-199.06)	220.1	0.73 (0.41-1.16)	0.95 (0.56-1.45)	30.27
Egypt	22.47 (18.68-26.77)	121.42 (97.12-153.96)	440.3	0.06 (0.05-0.06)	0.14 (0.11-0.17)	149.69
Puerto Rico	22.00 (20.14-24.08)	114.89 (94.27-137.73)	422.32	0.61 (0.56-0.67)	3.15 (2.57-3.78)	417.99
Israel	34.38 (31.37-37.83)	111.85 (99.40-124.63)	225.31	0.74 (0.67-0.81)	1.16 (1.03-1.30)	57.46
South Korea	90.08 (81.15-99.15)	111.22 (95.44-129.73)	23.47	0.21 (0.19-0.23)	0.16 (0.14-0.19)	-23.42
Ecuador	18.86 (16.96-21.06)	105.99 (83.36-135.56)	461.97	0.23 (0.20-0.25)	0.59 (0.46-0.75)	159.05
New Zealand	25.72 (23.15-28.55)	105.16 (94.14-117.29)	308.91	0.71 (0.64-0.79)	1.87 (1.67-2.09)	163.6

Czech Republic	56.66 (49.31-65.23)	100.88 (81.42-121.47)	78.06	0.50 (0.43-0.58)	0.77 (0.62-0.92)	53.76
Rwanda	40.21 (26.85-60.46)	99.77 (55.18-155.20)	148.15	0.97 (0.64-1.45)	0.96 (0.55-1.40)	-1.83
Turkmenistan	10.62 (8.64-12.93)	98.72 (65.46-141.94)	829.99	0.36 (0.30-0.43)	1.92 (1.28-2.76)	430.04
Mozambique	22.13 (13.58-31.45)	94.22 (59.97-140.38)	325.73	0.25 (0.16-0.36)	0.46 (0.30-0.68)	82.07
Somalia	34.20 (17.74-64.59)	93.24 (46.60-174.43)	172.6	0.73 (0.38-1.32)	0.69 (0.35-1.22)	-4.66
Angola	23.14 (13.23-36.60)	87.15 (50.86-130.99)	276.64	0.33 (0.19-0.52)	0.39 (0.23-0.57)	17.75
Honduras	29.14 (23.35-36.16)	86.87 (57.85-128.17)	198.09	0.89 (0.72-1.10)	0.99 (0.69-1.41)	10.72
Kyrgyzstan	37.10 (29.11-46.38)	84.96 (62.91-110.20)	129	1.00 (0.80-1.24)	1.33 (1.01-1.71)	32.65
Lithuania	63.34 (56.45-70.87)	83.07 (70.61-96.10)	31.15	1.60 (1.42-1.79)	2.76 (2.33-3.20)	73.12
Zimbabwe	37.47 (27.97-47.04)	82.74 (61.96-112.48)	120.84	0.70 (0.53-0.87)	0.82 (0.63-1.08)	17.36
Serbia	64.98 (52.82-81.03)	81.68 (64.08-103.55)	25.7	0.64 (0.52-0.80)	0.76 (0.58-0.97)	18.49
Myanmar	54.29 (40.20-73.92)	75.85 (55.67-100.71)	39.71	0.18 (0.13-0.24)	0.14 (0.11-0.19)	-19.24
Burundi	26.06 (16.14-43.75)	73.72 (38.13-121.41)	182.87	0.79 (0.48-1.29)	0.84 (0.44-1.35)	6.38
Chile	14.17 (12.92-15.47)	72.04 (64.23-81.34)	408.43	0.12 (0.11-0.13)	0.32 (0.28-0.36)	174.45
Saudi Arabia	8.59 (5.77-11.84)	68.83 (48.95-97.45)	701.41	0.07 (0.05-0.10)	0.15 (0.11-0.20)	100.52
Argentina	16.64 (14.92-18.41)	68.17 (59.56-79.83)	309.71	0.05 (0.05-0.06)	0.13 (0.12-0.16)	152.89
Croatia	49.84 (43.66-56.59)	65.39 (54.37-78.05)	31.2	0.92 (0.81-1.04)	1.24 (1.04-1.49)	34.84
United Arab Emirates	11.67 (8.29-15.53)	64.91 (48.69-84.51)	456.17	0.69 (0.51-0.90)	0.67 (0.52-0.85)	-3.16
Estonia	46.74 (41.52-52.08)	63.38 (50.12-75.80)	35.61	2.74 (2.43-3.05)	4.35 (3.44-5.17)	58.63
Nigeria	37.84 (24.97-52.11)	62.05 (36.83-88.95)	64	0.08 (0.05-0.10)	0.06 (0.04-0.08)	-24.54
Bolivia	21.34 (16.67-27.29)	60.59 (42.67-84.17)	183.88	0.42 (0.34-0.54)	0.53 (0.39-0.74)	26.34
Bulgaria	39.42 (33.44-46.07)	55.93 (44.93-70.29)	41.89	0.42 (0.36-0.49)	0.71 (0.56-0.90)	68.49
Eritrea	14.42 (9.01-25.10)	54.79 (29.85-91.61)	279.95	0.75 (0.48-1.29)	1.05 (0.59-1.68)	38.87
Malaysia	24.78 (16.63-29.67)	54.59 (41.97-64.82)	120.33	0.19 (0.12-0.22)	0.17 (0.13-0.20)	-8.18
Hungary	34.72 (30.40-39.04)	50.21 (41.01-61.27)	44.64	0.30 (0.27-0.34)	0.45 (0.36-0.55)	47.31
South Sudan	23.22 (12.75-36.78)	43.73 (23.61-71.55)	88.28	0.72 (0.41-1.11)	0.75 (0.41-1.22)	4.25

Kuwait	5.70 (4.68-7.52)	43.32 (32.51-60.06)	660.59	0.38 (0.32-0.50)	0.79 (0.60-1.07)	105.49
Latvia	44.24 (39.65-49.79)	42.76 (35.65-50.72)	-3.35	1.53 (1.37-1.74)	2.03 (1.69-2.40)	32.61
Haiti	11.30 (8.59-15.00)	42.22 (28.76-59.70)	273.82	0.22 (0.17-0.29)	0.35 (0.24-0.49)	54.38
Moldova	32.60 (29.17-36.45)	41.38 (33.71-48.95)	26.95	0.72 (0.65-0.80)	0.93 (0.75-1.11)	29.61
Azerbaijan	17.03 (13.21-21.87)	36.06 (22.37-52.77)	111.74	0.25 (0.20-0.32)	0.31 (0.20-0.45)	24.97
Slovakia	28.12 (22.82-34.78)	34.21 (26.17-44.21)	21.67	0.51 (0.41-0.63)	0.53 (0.40-0.70)	3.83
Venezuela	19.86 (16.86-22.98)	33.13 (22.77-45.68)	66.82	0.12 (0.10-0.14)	0.12 (0.08-0.17)	0.01
El Salvador	24.28 (20.16-29.06)	29.08 (22.58-36.26)	19.73	0.56 (0.47-0.66)	0.45 (0.35-0.56)	-19.58
Mauritius	0.64 (0.57-0.73)	27.37 (23.74-30.89)	4207.35	0.06 (0.06-0.07)	1.77 (1.53-2.01)	2643.75
Lebanon	11.98 (9.19-15.74)	26.91 (21.90-33.18)	124.54	0.49 (0.38-0.64)	0.43 (0.34-0.53)	-12.65
Tajikistan	10.14 (7.72-13.10)	26.46 (15.40-42.54)	160.91	0.25 (0.19-0.32)	0.28 (0.17-0.43)	11.12
Slovenia	17.80 (15.50-20.58)	25.64 (20.59-31.76)	44.02	0.81 (0.70-0.95)	1.04 (0.82-1.29)	27.46
Albania	22.08 (17.44-27.57)	24.97 (17.84-34.55)	13.08	0.69 (0.55-0.87)	0.81 (0.58-1.12)	16.47
Cambodia	11.11 (8.60-14.84)	24.11 (16.72-32.97)	116.92	0.18 (0.14-0.23)	0.16 (0.12-0.22)	-7.92
Congo	7.01 (4.20-10.36)	22.62 (13.53-35.21)	222.95	0.42 (0.25-0.61)	0.48 (0.29-0.73)	13.66
Georgia	15.75 (13.27-18.95)	22.09 (17.40-27.48)	40.24	0.27 (0.23-0.32)	0.50 (0.39-0.64)	87.98
Costa Rica	6.31 (5.43-7.23)	20.98 (17.83-24.45)	232.46	0.25 (0.21-0.28)	0.40 (0.34-0.46)	59.6
Iceland	5.77 (5.20-6.40)	20.57 (18.02-23.16)	256.31	2.13 (1.92-2.35)	5.26 (4.59-5.93)	147.44
Mongolia	3.97 (2.40-5.90)	19.19 (13.43-26.30)	383.96	0.24 (0.15-0.34)	0.60 (0.42-0.82)	151.92
Panama	6.32 (5.65-7.01)	16.52 (12.81-20.00)	161.28	0.29 (0.27-0.32)	0.38 (0.29-0.45)	27.43
Cyprus	8.85 (7.20-10.97)	16.37 (13.09-20.27)	84.94	1.14 (0.93-1.41)	1.05 (0.83-1.30)	-7.71
Luxembourg	8.63 (7.91-9.51)	16.08 (14.27-18.21)	86.44	2.01 (1.84-2.21)	2.05 (1.82-2.32)	2.1
Uruguay	3.68 (3.39-4.00)	15.42 (13.81-17.37)	319.21	0.11 (0.10-0.12)	0.35 (0.32-0.40)	224.01
Oman	4.98 (3.17-6.99)	14.38 (10.42-19.16)	188.95	0.34 (0.22-0.47)	0.29 (0.22-0.37)	-13.65
Central African Republic	6.40 (3.53-10.81)	13.79 (7.04-22.88)	115.56	0.33 (0.19-0.56)	0.33 (0.17-0.54)	-0.61
Jordan	4.79 (3.96-5.70)	13.25 (10.29-16.46)	176.49	0.20 (0.17-0.24)	0.12 (0.10-0.15)	-38.18

Paraguay	1.09 (0.93-1.29)	12.96 (9.94-16.63)	1084.62	0.03 (0.03-0.04)	0.18 (0.14-0.23)	451.05
Djibouti	2.20 (1.11-3.42)	12.18 (6.45-19.02)	452.52	0.87 (0.45-1.31)	1.06 (0.59-1.64)	22.46
Macedonia	7.20 (5.26-9.58)	11.83 (8.71-16.06)	64.24	0.35 (0.26-0.46)	0.46 (0.33-0.64)	31.87
Namibia	4.92 (3.72-6.50)	10.58 (7.16-14.82)	115.13	0.62 (0.48-0.79)	0.62 (0.43-0.84)	-0.01
Nicaragua	4.58 (3.83-5.34)	10.11 (8.18-12.74)	120.9	0.17 (0.14-0.19)	0.15 (0.13-0.19)	-6.91
Cameroon	4.57 (3.12-6.30)	10.09 (6.59-14.36)	120.68	0.09 (0.06-0.13)	0.06 (0.04-0.09)	-31.04
Papua New Guinea	5.12 (2.06-9.20)	9.62 (4.92-15.96)	87.87	0.17 (0.07-0.29)	0.12 (0.06-0.19)	-29.07
Laos	4.80 (3.53-6.77)	9.55 (6.80-13.01)	99.13	0.18 (0.13-0.25)	0.15 (0.11-0.20)	-13.87
Botswana	4.61 (3.18-6.50)	9.54 (6.35-13.29)	107	0.64 (0.45-0.87)	0.50 (0.35-0.66)	-21.98
Lesotho	4.43 (3.40-5.72)	9.36 (6.60-12.78)	111.14	0.47 (0.37-0.61)	0.70 (0.51-0.95)	47.73
Singapore	3.71 (3.30-4.14)	9.21 (8.06-10.51)	148.43	0.11 (0.10-0.12)	0.13 (0.11-0.15)	13.33
Cuba	21.78 (16.45-25.30)	8.87 (7.41-10.42)	-59.26	0.19 (0.14-0.22)	0.06 (0.05-0.08)	-66.5
Armenia	4.94 (4.07-6.03)	8.60 (6.94-10.77)	74.27	0.15 (0.12-0.18)	0.24 (0.19-0.30)	57.85
Dominican Republic	3.59 (2.89-4.40)	8.60 (6.49-11.39)	139.55	0.06 (0.05-0.07)	0.08 (0.06-0.10)	24.61
Cote d'Ivoire	4.56 (3.22-6.51)	8.47 (5.57-12.12)	85.7	0.09 (0.06-0.12)	0.06 (0.04-0.09)	-32.16
Gabon	3.33 (2.11-4.75)	7.67 (4.43-11.39)	130.25	0.46 (0.29-0.64)	0.49 (0.28-0.73)	7.08
Malta	2.37 (2.11-2.68)	7.42 (6.37-8.81)	212.98	0.61 (0.55-0.70)	1.53 (1.31-1.84)	149.35
Comoros	2.11 (0.88-3.25)	6.82 (4.00-10.09)	223.74	0.83 (0.38-1.27)	1.06 (0.64-1.56)	27.72
Trinidad and Tobago	0.85 (0.78-0.94)	6.43 (4.68-8.42)	656.17	0.08 (0.07-0.09)	0.41 (0.30-0.53)	421.4
Ghana	3.49 (2.34-4.97)	6.40 (4.06-9.48)	83.44	0.03 (0.02-0.04)	0.02 (0.01-0.03)	-28.75
Qatar	0.95 (0.69-1.24)	6.27 (4.22-8.57)	559.53	0.27 (0.21-0.33)	0.21 (0.15-0.27)	-22.8
Senegal	3.21 (2.08-4.57)	6.04 (3.81-8.74)	88.57	0.09 (0.06-0.12)	0.07 (0.04-0.09)	-23.88
Bosnia and Herzegovina	6.42 (5.20-8.08)	5.96 (4.37-8.03)	-7.05	0.13 (0.11-0.17)	0.15 (0.11-0.20)	12.88
Mali	3.14 (1.82-4.59)	5.79 (3.03-8.37)	84.25	0.08 (0.04-0.11)	0.06 (0.03-0.08)	-26.97
Equatorial Guinea	0.96 (0.55-1.56)	5.76 (3.20-9.36)	499.02	0.34 (0.19-0.55)	0.47 (0.26-0.74)	39.26
Kiribati	2.51 (1.93-3.21)	5.58 (3.88-7.72)	122.59	3.84 (3.05-4.83)	4.97 (3.54-6.82)	29.23

Swaziland	2.51 (1.90-3.28)	5.52 (3.60-7.83)	119.97	0.63 (0.48-0.80)	0.70 (0.47-0.96)	11.34
Burkina Faso	3.19 (1.88-4.91)	5.03 (3.09-7.50)	57.65	0.07 (0.04-0.11)	0.05 (0.03-0.07)	-32.67
Bahrain	0.95 (0.78-1.16)	4.66 (3.70-5.77)	387.73	0.25 (0.22-0.28)	0.29 (0.24-0.35)	18.25
Niger	2.18 (1.12-3.43)	4.40 (1.88-7.35)	101.49	0.07 (0.03-0.10)	0.04 (0.02-0.07)	-32.72
Jamaica	2.11 (1.72-2.61)	4.01 (2.93-5.48)	89.82	0.11 (0.09-0.14)	0.13 (0.09-0.18)	17.69
Chad	1.99 (1.14-2.92)	3.89 (2.30-5.89)	95.82	0.06 (0.03-0.09)	0.05 (0.03-0.08)	-11.89
Bhutan	1.64 (0.90-2.29)	3.72 (1.97-5.31)	126.81	0.44 (0.26-0.60)	0.53 (0.29-0.74)	20.45
Guinea	2.43 (1.45-3.39)	3.72 (2.26-5.47)	52.92	0.07 (0.04-0.10)	0.06 (0.03-0.08)	-21.02
Benin	1.82 (1.23-2.52)	3.27 (2.03-4.77)	79.87	0.08 (0.05-0.11)	0.05 (0.03-0.07)	-35.43
Suriname	0.43 (0.32-0.51)	3.13 (2.41-4.10)	628.84	0.12 (0.09-0.15)	0.51 (0.39-0.67)	310.23
Brunei	1.67 (1.33-2.11)	2.67 (2.19-3.18)	60.12	0.69 (0.57-0.84)	0.51 (0.43-0.61)	-25.32
Togo	1.21 (0.83-1.74)	2.39 (1.53-3.56)	97.26	0.08 (0.05-0.11)	0.05 (0.03-0.08)	-32.88
Sierra Leone	1.63 (1.08-2.35)	2.36 (1.39-3.54)	45.03	0.07 (0.05-0.10)	0.05 (0.03-0.07)	-29.69
Maldives	0.20 (0.14-0.28)	2.01 (1.44-2.72)	907.27	0.17 (0.12-0.24)	0.32 (0.24-0.43)	88.81
Virgin Islands, U.S.	1.55 (1.22-1.92)	1.68 (1.18-2.40)	8.25	1.44 (1.13-1.77)	2.33 (1.57-3.42)	61.79
Mauritania	0.98 (0.71-1.28)	1.54 (1.03-2.20)	57.19	0.08 (0.06-0.11)	0.06 (0.04-0.09)	-24.26
Liberia	1.10 (0.64-1.70)	1.49 (0.79-2.53)	35.88	0.08 (0.05-0.13)	0.06 (0.03-0.09)	-33.78
Greenland	0.91 (0.75-1.11)	1.48 (1.14-1.86)	62.9	1.48 (1.23-1.78)	2.31 (1.76-2.91)	55.8
Timor-Leste	0.68 (0.39-0.94)	1.38 (0.96-1.86)	104.32	0.14 (0.08-0.19)	0.13 (0.09-0.18)	-3.09
Montenegro	1.10 (0.85-1.38)	1.37 (1.03-1.79)	25.13	0.17 (0.13-0.21)	0.20 (0.15-0.26)	18.07
Guyana	0.19 (0.16-0.23)	1.22 (0.87-1.62)	527.97	0.03 (0.03-0.04)	0.16 (0.11-0.21)	428.9
Palestine	0.30 (0.20-0.41)	1.05 (0.80-1.36)	252.43	0.02 (0.02-0.03)	0.03 (0.02-0.03)	11.97
The Bahamas	0.32 (0.28-0.36)	0.96 (0.72-1.25)	199.49	0.13 (0.11-0.15)	0.22 (0.17-0.29)	66.2
The Gambia	0.38 (0.25-0.57)	0.86 (0.56-1.24)	123.39	0.08 (0.05-0.12)	0.06 (0.04-0.09)	-23.27
Grenada	0.15 (0.13-0.16)	0.76 (0.63-0.93)	423.84	0.20 (0.18-0.23)	0.68 (0.56-0.83)	235.08
Seychelles	0.22 (0.19-0.24)	0.76 (0.64-0.89)	250.66	0.34 (0.31-0.38)	0.64 (0.54-0.75)	87.67

Solomon Islands	0.44 (0.23-0.76)	0.73 (0.46-1.11)	64.74	0.19 (0.10-0.32)	0.13 (0.09-0.21)	-29.82
Fiji	1.35 (1.05-1.67)	0.72 (0.54-0.95)	-46.77	0.21 (0.17-0.26)	0.09 (0.07-0.11)	-59.78
Bermuda	0.32 (0.29-0.36)	0.63 (0.50-0.79)	98.04	0.46 (0.42-0.52)	0.80 (0.63-1.01)	71.85
Guinea-Bissau	0.43 (0.27-0.65)	0.60 (0.39-0.89)	38.87	0.09 (0.06-0.13)	0.06 (0.04-0.09)	-35.21
Barbados	0.13 (0.12-0.14)	0.53 (0.39-0.70)	307.34	0.05 (0.04-0.05)	0.15 (0.11-0.20)	211.51
Belize	0.04 (0.04-0.05)	0.44 (0.37-0.51)	893.37	0.03 (0.03-0.03)	0.10 (0.09-0.12)	223.41
Cape Verde	0.23 (0.14-0.32)	0.41 (0.30-0.59)	80.39	0.08 (0.05-0.11)	0.08 (0.05-0.10)	-2.44
Sao Tome and Principe	0.10 (0.06-0.14)	0.40 (0.26-0.57)	285.5	0.13 (0.09-0.17)	0.24 (0.17-0.32)	83.21
Saint Lucia	0.07 (0.06-0.07)	0.36 (0.28-0.44)	430.46	0.06 (0.06-0.07)	0.17 (0.14-0.21)	175.99
Dominica	0.08 (0.07-0.10)	0.33 (0.26-0.42)	286.97	0.14 (0.12-0.16)	0.45 (0.35-0.58)	229.58
Vanuatu	0.21 (0.13-0.32)	0.33 (0.22-0.47)	60.66	0.19 (0.13-0.29)	0.13 (0.09-0.18)	-31.29
Samoa	0.30 (0.19-0.41)	0.26 (0.17-0.37)	-13.32	0.23 (0.15-0.32)	0.15 (0.10-0.21)	-35.51
Antigua and Barbuda	0.07 (0.06-0.07)	0.25 (0.22-0.28)	285.85	0.11 (0.10-0.13)	0.24 (0.21-0.27)	112.28
Guam	0.53 (0.39-0.65)	0.21 (0.17-0.25)	-61.08	0.40 (0.31-0.48)	0.11 (0.09-0.13)	-72.77
Saint Vincent and the Grenadines	0.03 (0.03-0.04)	0.16 (0.13-0.19)	425.03	0.03 (0.03-0.04)	0.13 (0.10-0.16)	265.48
Federated States of Micronesia	0.19 (0.14-0.26)	0.15 (0.10-0.21)	-21.78	0.25 (0.18-0.35)	0.16 (0.11-0.23)	-35.56
American Samoa	0.07 (0.06-0.10)	0.13 (0.10-0.16)	70.11	0.20 (0.16-0.26)	0.27 (0.21-0.34)	32.6
Saint Kitts and Nevis	0.06 (0.05-0.07)	0.13 (0.10-0.16)	111.79	0.16 (0.14-0.18)	0.18 (0.15-0.23)	13.49
Tonga	0.13 (0.07-0.18)	0.12 (0.08-0.17)	-8.18	0.18 (0.10-0.24)	0.14 (0.09-0.19)	-24.57
Northern Mariana Islands	0.12 (0.08-0.16)	0.09 (0.07-0.11)	-23.89	0.27 (0.20-0.36)	0.18 (0.15-0.22)	-33.68
Andorra	0.06 (0.04-0.08)	0.08 (0.05-0.10)	34.73	0.08 (0.06-0.12)	0.08 (0.06-0.11)	-6.11
Marshall Islands	0.08 (0.05-0.11)	0.08 (0.06-0.12)	11.44	0.24 (0.18-0.33)	0.17 (0.12-0.25)	-27.8
Monaco	0.01 (0.01-0.02)	0.07 (0.05-0.10)	386.77	0.05 (0.03-0.06)	0.21 (0.14-0.31)	348.55
Cook Islands	0.02 (0.02-0.03)	0.04 (0.03-0.05)	62.08	0.14 (0.11-0.17)	0.18 (0.13-0.23)	28.86
San Marino	0.02 (0.02-0.03)	0.03 (0.02-0.04)	37.41	0.08 (0.07-0.10)	0.09 (0.05-0.13)	6.36
Nauru	0.02 (0.01-0.03)	0.02 (0.01-0.03)	-29.39	0.28 (0.20-0.41)	0.17 (0.11-0.28)	-40.15

Tuvalu	0.02 (0.01-0.03)	0.02 (0.01-0.03)	8.39	0.22 (0.16-0.31)	0.17 (0.12-0.24)	-23.84
Niue	0.01 (0.00-0.01)	0.00 (0.00-0.00)	-43.95	0.25 (0.18-0.34)	0.17 (0.12-0.22)	-33.83
Palau	0.00 (0.00-0.00)	0.00 (0.00-0.00)	49.51	0.01 (0.01-0.01)	0.01 (0.01-0.01)	28.8
Tokelau	0.00 (0.00-0.00)	0.00 (0.00-0.00)	-26.66	0.26 (0.18-0.35)	0.19 (0.15-0.25)	-26.41

Table S9. DALYs, ASDRs, and their changes in drug use disorders across 204 countries and territories from 1990 to 2021

Country	Number of DALYs			ASDR (per 100,000 population)		
	1990	2021	Change (%)	1990	2021	Change (%)
	Both (95%UI)	Both (95%UI)		Both (95%UI)	Both (95%UI)	
United States	973680.24 (754471.58-1185672.41)	6484690.40 (5471717.29-7481321.26)	566	352.71 (272.69-429.65)	1944.08 (1632.99-2249.41)	451.19
China	3550436.06 (2841944.88-4224557.20)	1661208.48 (1278813.66-2030011.63)	-53.21	272.83 (220.61-321.52)	116.47 (89.13-143.59)	-57.31
India	553170.23 (433302.61-684407.39)	1229324.19 (963582.06-1506573.26)	122.23	71.92 (56.97-87.98)	81.52 (64.47-98.94)	13.35
Russian Federation	589034.04 (468880.68-695059.18)	659538.54 (561314.81-757397.06)	11.97	371.69 (295.89-440.78)	456.02 (385.23-526.69)	22.69
United Kingdom	143619.41 (113338.64-172617.63)	370988.45 (318124.07-421932.29)	158.31	243.89 (191.80-294.14)	554.29 (472.29-632.12)	127.27
Canada	104143.56 (79549.37-128800.36)	322467.47 (279922.35-364203.71)	209.64	346.59 (265.58-430.44)	877.19 (759.19-998.21)	153.09
Iran	161546.75 (133012.47-189976.56)	321123.96 (268927.05-373935.04)	98.78	339.74 (281.57-397.18)	339.36 (282.07-397.38)	-0.11
Brazil	159243.86 (108790.85-209643.18)	306463.89 (234174.76-376925.19)	92.45	102.77 (71.68-134.29)	131.43 (99.94-162.15)	27.89
Germany	165212.81 (135413.50-193806.96)	192914.74 (158897.12-225741.95)	16.77	194.12 (159.04-228.19)	235.19 (192.38-277.40)	21.16
Pakistan	67950.14 (52014.93-87158.46)	160868.99 (123985.22-201662.39)	136.75	75.25 (58.15-94.47)	72.36 (56.70-88.86)	-3.83
Vietnam	70105.17 (53980.72-89421.27)	146961.63 (111275.02-185576.44)	109.63	104.09 (81.28-129.59)	140.56 (107.02-176.80)	35.04
Indonesia	83642.92 (56832.13-117186.24)	142366.22 (100397.10-192681.23)	70.21	43.78 (30.28-59.82)	47.44 (33.63-64.09)	8.36
France	84730.20 (67478.98-102826.61)	138400.13 (115480.89-161523.85)	63.34	140.69 (111.80-171.62)	200.59 (165.19-237.62)	42.58
Australia	71951.50 (58464.71-84883.48)	131560.64 (111227.23-151449.22)	82.85	399.17 (324.22-470.93)	502.09 (420.60-582.79)	25.78
Ukraine	126423.45 (102808.21-150239.69)	120508.49 (95752.25-147203.64)	-4.68	223.44 (180.95-267.11)	279.62 (219.77-344.82)	25.14
Mexico	71714.24 (53621.34-91514.93)	119551.15 (91663.69-149850.47)	66.7	85.83 (64.90-109.36)	86.88 (66.56-108.77)	1.23
Bangladesh	55016.28 (40569.37-71752.62)	110108.89 (81723.69-141970.31)	100.14	61.28 (46.17-78.23)	64.28 (48.17-82.75)	4.89

Egypt	44195.45 (29484.70-58950.62)	103306.30 (71061.82-135564.92)	133.75	84.31 (57.37-111.06)	99.09 (68.94-128.83)	17.53
South Africa	70767.90 (55701.40-85711.29)	95461.25 (77951.10-112747.97)	34.89	201.24 (159.91-240.46)	157.73 (129.31-185.46)	-21.62
Japan	111183.58 (79375.73-145766.36)	94571.85 (70378.15-120981.43)	-14.94	85.95 (61.12-113.56)	85.30 (62.08-111.73)	-0.75
Spain	104156.10 (84381.26-125242.64)	93863.50 (75485.15-114315.04)	-9.88	259.39 (210.45-311.40)	218.13 (170.99-268.00)	-15.91
Turkey	59356.94 (42245.43-78388.90)	92809.71 (67604.76-117398.23)	56.36	104.03 (75.48-136.23)	104.49 (76.03-132.91)	0.44
Algeria	29885.35 (23251.53-37785.24)	75711.58 (58693.11-93667.72)	153.34	133.79 (106.04-166.23)	167.13 (129.49-207.11)	24.92
Italy	154889.54 (125984.41-184574.77)	74047.99 (56080.22-90333.03)	-52.19	262.19 (213.15-312.22)	138.92 (104.24-173.13)	-47.01
Philippines	37012.87 (26107.53-48955.16)	69767.90 (48831.06-90873.22)	88.5	60.04 (42.96-78.15)	57.95 (41.23-74.90)	-3.49
Sudan	21464.97 (16364.73-27426.21)	67702.10 (52052.84-83607.75)	215.41	126.87 (97.46-159.88)	161.57 (124.37-198.88)	27.34
Thailand	62472.85 (41567.68-87095.40)	67034.18 (47528.65-88162.62)	7.3	96.15 (65.07-132.14)	103.34 (72.40-136.78)	7.48
Iraq	21425.35 (16538.57-26869.75)	66710.83 (52492.02-84974.83)	211.36	137.77 (108.20-169.29)	159.03 (125.92-202.94)	15.43
Nigeria	26554.43 (18911.40-34650.06)	65977.20 (46950.88-86392.67)	148.46	34.86 (25.36-44.42)	33.93 (24.41-43.68)	-2.67
Morocco	33535.36 (25548.04-42637.75)	64610.83 (50122.18-80784.70)	92.66	140.09 (107.80-175.01)	165.53 (128.50-206.81)	18.16
Kazakhstan	31771.25 (24717.89-39530.52)	61430.03 (49033.50-74072.77)	93.35	189.55 (147.86-233.05)	314.08 (249.39-378.82)	65.7
Afghanistan	13742.20 (10615.32-16887.86)	54152.69 (43238.47-66734.01)	294.06	186.58 (145.33-228.33)	203.76 (162.87-250.12)	9.21
South Korea	51572.48 (36474.84-67811.27)	51828.39 (36683.98-67414.45)	0.5	99.29 (70.42-129.96)	99.31 (69.68-130.25)	0.02
Colombia	32038.22 (23644.06-41419.62)	51814.94 (39787.36-66076.79)	61.73	94.27 (70.11-120.64)	96.46 (73.86-123.08)	2.33
Argentina	33334.06 (22442.87-45035.18)	50326.01 (35767.03-66734.95)	50.97	103.91 (70.08-140.39)	105.17 (74.47-140.02)	1.22
Tanzania	15746.37 (11633.78-20978.92)	46386.29 (33158.47-61106.19)	194.58	82.51 (59.88-109.61)	95.07 (68.53-125.50)	15.22
Democratic Republic of the Congo	15821.88 (11722.46-20753.67)	46199.27 (33346.39-60512.05)	192	51.01 (37.94-66.17)	58.25 (42.28-75.62)	14.2
Saudi Arabia	14145.55 (9556.42-18994.51)	45734.70 (32214.91-60908.36)	223.32	90.45 (61.09-118.41)	93.75 (65.88-124.07)	3.65
Ethiopia	18454.84 (13923.51-24123.89)	44352.83 (33204.86-57274.04)	140.33	47.88 (36.62-62.07)	45.56 (34.27-57.71)	-4.84
Sweden	10810.37 (8926.38-12721.60)	41991.48 (34777.96-49150.85)	288.44	125.47 (103.75-147.84)	430.88 (354.39-505.09)	243.4
Taiwan (Province of China)	35125.43 (28573.48-42123.30)	41919.97 (33592.24-50851.21)	19.34	153.93 (126.31-182.70)	165.50 (130.50-203.15)	7.52
Poland	41604.03 (32041.28-51449.00)	41146.13 (32491.35-50033.59)	-1.1	109.63 (83.82-136.69)	115.61 (90.21-142.29)	5.46
Yemen	10372.16 (7560.71-13972.06)	39974.70 (29242.70-52827.54)	285.4	106.37 (78.72-141.04)	133.48 (98.84-174.39)	25.5

Uzbekistan	21110.01 (14847.86-28366.74)	38852.01 (27983.51-48594.19)	84.05	105.49 (74.75-138.41)	107.79 (77.72-134.72)	2.18
Myanmar	27429.01 (19364.40-37600.94)	36112.18 (24967.26-47949.34)	31.66	65.89 (47.11-87.85)	61.29 (42.51-81.05)	-6.98
Peru	17297.85 (12739.44-22246.43)	34169.34 (26341.98-42675.53)	97.54	81.89 (61.06-104.08)	87.87 (68.13-109.57)	7.3
North Korea	28825.30 (22061.44-36111.82)	32977.93 (25136.30-42224.87)	14.41	133.18 (102.78-166.23)	113.50 (85.73-146.30)	-14.78
Netherlands	20743.20 (15467.15-26299.71)	27563.17 (22125.72-33464.41)	32.88	126.71 (94.18-160.63)	168.58 (133.69-205.61)	33.04
Finland	14864.79 (12648.70-17098.21)	26799.59 (23007.65-30716.38)	80.29	273.71 (231.04-315.97)	517.31 (441.03-595.09)	89
Uganda	8318.39 (5990.13-10798.09)	26233.80 (19419.64-34434.39)	215.37	65.87 (47.32-86.26)	77.94 (57.52-102.99)	18.33
Norway	12210.54 (9876.70-14614.16)	25752.48 (21407.85-29953.86)	110.9	276.95 (224.16-331.68)	466.20 (387.62-544.62)	68.33
Belarus	26411.20 (19609.20-33966.94)	24556.30 (19974.27-29342.59)	-7.02	241.42 (178.96-311.95)	249.96 (203.26-299.17)	3.54
Malaysia	12170.93 (8437.34-16335.78)	24479.33 (17277.75-32258.86)	101.13	68.10 (47.71-89.57)	67.79 (48.14-88.82)	-0.45
United Arab Emirates	2991.76 (2237.56-3815.96)	24149.81 (17987.69-30404.48)	707.21	133.16 (101.20-169.22)	200.82 (149.90-252.32)	50.81
Chile	14643.83 (10357.07-19187.47)	23927.01 (17769.61-30523.25)	63.39	102.20 (72.67-134.01)	121.03 (89.38-154.42)	18.42
Belgium	14099.55 (10956.22-17422.36)	23874.76 (19933.02-27918.96)	69.33	135.58 (105.48-168.10)	214.45 (177.84-253.00)	58.17
Switzerland	40934.07 (33369.16-48026.18)	23726.41 (19859.50-27775.09)	-42.04	553.92 (451.47-652.04)	269.87 (224.64-317.70)	-51.28
Greece	14520.87 (11396.92-17609.99)	23603.94 (19830.10-27178.65)	62.55	138.98 (109.01-168.52)	256.32 (212.32-296.43)	84.42
Denmark	18685.90 (15670.32-21958.19)	22161.65 (18557.73-25555.66)	18.6	339.41 (283.80-400.08)	378.43 (314.58-437.72)	11.5
Austria	16963.46 (13156.51-20718.38)	21747.87 (17849.27-25514.27)	28.2	200.99 (155.81-245.30)	251.89 (204.95-297.04)	25.33
Kenya	7514.57 (5622.06-9559.18)	21683.09 (16466.06-26836.87)	188.55	45.29 (33.32-56.53)	48.60 (37.50-58.62)	7.32
Tunisia	10256.69 (7906.60-12899.18)	20564.29 (15802.74-25526.17)	100.5	129.05 (100.36-161.74)	165.06 (127.32-203.38)	27.9
Nepal	10437.11 (7697.82-13676.95)	20468.72 (15064.43-26465.41)	96.11	63.57 (47.44-81.53)	63.95 (47.60-81.63)	0.59
Libya	5311.09 (4100.30-6601.06)	20407.25 (16607.01-25162.83)	284.24	146.97 (116.72-179.59)	251.47 (204.48-310.75)	71.11
Guatemala	7523.33 (6340.00-8774.40)	20305.95 (16931.04-23914.76)	169.91	117.52 (99.96-135.90)	124.14 (104.01-145.53)	5.63
Sri Lanka	21527.00 (16450.08-26271.81)	20070.13 (15191.84-25317.44)	-6.77	121.71 (94.23-146.76)	88.35 (66.28-111.91)	-27.41
Ireland	6450.55 (5216.37-7863.13)	19792.15 (16466.93-22921.86)	206.83	180.28 (145.99-220.03)	414.53 (342.12-481.53)	129.93
Syria	12143.58 (9365.09-15377.64)	17882.23 (13826.30-21895.19)	47.26	113.84 (89.17-141.36)	134.46 (103.85-164.48)	18.11
Ecuador	7503.72 (5431.45-9723.75)	17797.96 (13958.66-22248.78)	137.19	76.57 (55.49-98.07)	94.00 (73.58-116.86)	22.77

Madagascar	5914.47 (4466.93-7666.04)	17132.20 (12463.98-22366.71)	189.67	64.61 (48.51-84.26)	69.86 (51.13-91.07)	8.13
Venezuela	13083.94 (9283.99-17421.15)	16597.29 (11744.44-21426.11)	26.85	69.35 (49.93-91.04)	63.70 (45.37-82.72)	-8.15
Romania	21035.18 (16624.53-25701.91)	16443.12 (13629.75-19271.15)	-21.83	88.48 (69.61-108.59)	86.52 (71.04-102.34)	-2.21
Angola	4696.06 (3426.46-5971.38)	16071.16 (11900.02-20681.43)	242.23	55.06 (40.52-69.71)	60.50 (44.59-77.78)	9.87
Mozambique	4921.19 (3634.29-6380.89)	15691.79 (11574.65-20502.95)	218.86	46.73 (34.85-58.99)	64.16 (48.75-82.74)	37.28
Ghana	5630.63 (3835.40-7486.89)	14929.99 (10015.38-19936.52)	165.16	43.82 (29.90-58.07)	44.81 (30.18-59.47)	2.25
Zambia	4674.67 (3499.61-6028.12)	14882.00 (10778.18-19886.40)	218.35	81.46 (60.77-107.02)	90.76 (66.66-120.63)	11.42
New Zealand	7205.06 (5324.06-9093.37)	14155.13 (11367.97-16868.77)	96.46	197.94 (146.45-249.87)	273.84 (217.67-327.59)	38.34
Zimbabwe	8196.94 (6004.83-10498.62)	14067.52 (10693.23-17644.91)	71.62	105.20 (79.08-133.93)	106.19 (81.88-130.44)	0.94
Israel	6278.71 (4802.81-7693.27)	14044.73 (11250.32-16816.25)	123.69	129.87 (99.73-158.19)	153.65 (122.92-184.31)	18.32
Portugal	13687.96 (10731.04-16440.75)	13840.38 (11179.16-16538.41)	1.11	133.31 (104.49-160.30)	138.20 (110.96-165.59)	3.67
Czech Republic	10342.53 (8020.01-12931.49)	13676.03 (10920.76-16658.24)	32.23	101.69 (78.00-127.98)	143.42 (113.30-176.56)	41.04
Malawi	5002.67 (3717.65-6678.04)	12937.97 (9407.04-17366.35)	158.62	66.67 (48.95-89.28)	79.89 (57.75-106.44)	19.83
Cameroon	3742.37 (2614.35-4979.93)	12492.22 (8637.13-16651.80)	233.8	44.31 (31.76-58.10)	43.97 (30.73-57.49)	-0.76
Bolivia	5740.62 (4220.90-7287.73)	12393.19 (9211.97-15650.09)	115.89	97.74 (72.52-122.82)	99.48 (74.10-125.62)	1.78
Azerbaijan	8896.41 (6274.09-11640.53)	12309.28 (9076.40-15759.03)	38.36	114.06 (80.39-147.76)	105.87 (77.21-135.95)	-7.18
Jordan	3281.43 (2245.30-4419.34)	12170.99 (8556.13-16214.01)	270.91	94.55 (65.81-123.25)	90.59 (63.86-119.88)	-4.18
Turkmenistan	4325.59 (3070.90-5581.91)	11629.85 (8851.16-14632.03)	168.86	121.97 (87.65-156.87)	215.12 (164.10-270.38)	76.37
Haiti	4521.12 (3226.54-5876.49)	11613.00 (8525.28-15083.00)	156.86	77.46 (56.62-99.34)	85.99 (63.57-110.51)	11.02
Kyrgyzstan	6712.00 (5189.44-8319.33)	11521.91 (9242.40-14181.75)	71.66	161.32 (126.42-198.84)	166.43 (134.29-204.05)	3.17
Somalia	3991.60 (2824.81-5871.79)	11136.80 (7646.89-16033.19)	179.01	65.49 (46.35-95.44)	65.97 (44.87-95.54)	0.72
Cambodia	5608.17 (3933.04-7502.70)	11135.35 (7753.82-15060.49)	98.56	60.78 (43.82-80.82)	60.94 (43.08-81.77)	0.25
Honduras	3964.55 (3214.41-4859.32)	11129.33 (8389.85-14210.07)	180.72	103.91 (84.40-125.53)	109.67 (83.47-138.69)	5.53
Tajikistan	5519.28 (3887.54-7321.23)	10834.25 (7787.53-13768.03)	96.3	111.75 (79.52-146.65)	103.68 (75.10-130.88)	-7.22
Lithuania	7781.03 (6209.41-9108.15)	10056.85 (8329.33-11910.94)	29.25	201.04 (160.17-235.54)	386.60 (317.58-458.25)	92.3
Rwanda	4178.86 (3124.04-5506.75)	10024.81 (7111.81-13396.36)	139.89	76.81 (57.99-102.27)	80.54 (57.06-106.74)	4.85

Cote d'Ivoire	4089.76 (2878.75-5543.45)	9828.45 (6733.77-13019.06)	140.32	39.59 (27.92-52.68)	38.76 (26.67-50.76)	-2.1
Hungary	9205.57 (6759.40-11799.37)	9575.33 (7309.64-12025.06)	4.02	90.67 (65.79-117.38)	109.92 (82.51-140.09)	21.22
Serbia	8713.51 (7032.22-10659.25)	9528.37 (7559.46-11531.67)	9.35	88.22 (70.84-108.46)	105.93 (83.54-127.73)	20.07
Estonia	5058.24 (4146.88-5857.79)	8760.94 (7066.45-10562.38)	73.2	315.86 (258.12-366.21)	733.92 (586.79-889.92)	132.36
Lebanon	3066.04 (2246.19-3946.34)	8718.23 (6503.56-11045.44)	184.35	107.35 (79.07-136.87)	141.39 (104.26-179.73)	31.71
Cuba	11481.91 (8135.53-14987.42)	8530.23 (6113.80-11237.54)	-25.71	92.18 (65.71-119.95)	78.16 (55.30-102.49)	-15.2
Dominican Republic	4542.23 (3142.26-6147.03)	8243.89 (5800.76-10930.36)	81.49	62.47 (43.47-82.80)	69.62 (48.97-92.23)	11.45
Puerto Rico	6080.78 (4492.73-7544.43)	8197.18 (6920.84-9685.82)	34.8	165.36 (122.48-205.14)	251.83 (212.35-298.31)	52.29
Burundi	2954.84 (2159.20-4012.93)	7868.22 (5638.83-10623.13)	166.28	69.16 (50.63-94.44)	71.49 (50.65-96.40)	3.37
Bulgaria	7965.06 (6120.15-9909.94)	7756.02 (6166.99-9369.25)	-2.62	93.68 (71.23-118.27)	130.03 (101.41-161.12)	38.79
Kuwait	2172.78 (1582.47-2873.05)	7513.36 (5828.98-9342.42)	245.8	106.42 (78.85-138.35)	128.51 (98.92-162.26)	20.76
Niger	2396.00 (1666.23-3316.49)	7311.48 (5087.03-10038.45)	205.15	39.11 (27.35-53.26)	38.58 (26.92-51.90)	-1.35
Croatia	6533.34 (5212.06-7808.18)	7248.93 (5835.67-8610.64)	10.95	128.87 (103.24-155.02)	179.36 (142.59-214.50)	39.18
Burkina Faso	2604.11 (1840.04-3419.89)	7212.61 (4952.82-9520.33)	176.97	36.85 (26.19-48.02)	38.59 (26.98-50.61)	4.74
Mali	2616.53 (1790.15-3471.58)	7137.02 (4933.03-9650.46)	172.77	38.49 (26.58-50.17)	36.87 (25.47-48.51)	-4.2
Papua New Guinea	2655.75 (1823.08-3621.85)	6965.66 (4861.90-9321.23)	162.29	66.36 (46.28-89.06)	65.57 (46.23-87.11)	-1.19
Paraguay	2817.81 (1910.22-3742.69)	6092.34 (4391.33-7937.68)	116.21	74.34 (50.87-98.76)	78.01 (56.42-101.02)	4.94
Moldova	7347.57 (5493.57-9165.46)	5875.32 (4602.94-7151.44)	-20.04	162.24 (121.95-201.48)	154.35 (119.94-191.23)	-4.86
Senegal	2626.65 (1844.42-3527.65)	5844.04 (3974.18-7879.93)	122.49	43.45 (30.68-57.11)	40.25 (27.73-53.38)	-7.36
Singapore	3673.95 (2556.40-4880.88)	5618.81 (4018.64-7430.74)	52.94	95.99 (67.14-127.03)	94.91 (66.68-126.65)	-1.13
Slovakia	6067.84 (4567.27-7510.96)	5558.29 (4250.61-6844.57)	-8.4	113.36 (85.21-140.66)	105.55 (80.68-130.45)	-6.88
El Salvador	4105.19 (3212.43-5157.81)	5475.88 (4188.36-6867.80)	33.39	84.95 (66.85-106.25)	81.50 (62.50-101.45)	-4.07
Eritrea	1778.22 (1287.08-2378.43)	5233.86 (3626.71-7176.51)	194.33	69.14 (50.71-93.30)	85.17 (59.14-117.54)	23.19
Oman	1804.71 (1299.62-2419.24)	5226.39 (3781.67-6776.66)	189.6	93.09 (67.00-121.43)	89.80 (64.70-115.19)	-3.53
Chad	1850.05 (1263.53-2508.84)	5042.20 (3434.73-6757.68)	172.54	39.65 (27.37-52.08)	37.39 (25.90-49.29)	-5.7
South Sudan	2974.90 (2175.20-3899.15)	5026.12 (3608.01-7036.91)	68.95	65.40 (47.63-86.70)	66.87 (48.19-94.65)	2.25

Latvia	6278.75 (4996.98-7385.62)	4784.32 (3905.12-5665.64)	-23.8	229.13 (181.71-270.11)	272.59 (220.70-323.57)	18.96
Benin	1475.72 (1012.52-1983.73)	4637.42 (3260.71-6228.14)	214.25	39.11 (27.24-52.25)	39.58 (28.24-52.56)	1.21
Laos	2122.21 (1498.30-2816.38)	4549.39 (3211.98-6037.02)	114.37	57.80 (41.42-75.92)	56.93 (40.92-74.67)	-1.5
Nicaragua	2370.85 (1682.69-3097.07)	4541.39 (3236.05-5852.36)	91.55	71.06 (50.77-92.33)	63.46 (45.27-80.97)	-10.7
Palestine	1624.55 (1083.40-2240.99)	4534.82 (3019.45-6147.71)	179.14	91.66 (62.55-124.23)	84.69 (56.46-114.03)	-7.6
Costa Rica	2411.40 (1731.46-3163.97)	4427.35 (3430.48-5592.32)	83.6	78.50 (56.71-101.67)	85.71 (66.16-107.90)	9.18
Mongolia	2347.33 (1657.96-3087.85)	4321.35 (3218.70-5432.58)	84.1	114.09 (80.19-147.85)	129.55 (96.31-163.39)	13.55
Uruguay	3712.53 (2620.80-4914.01)	4186.53 (3082.13-5300.22)	12.77	120.78 (85.22-160.32)	122.11 (89.36-155.52)	1.1
Georgia	7149.41 (5069.30-9370.33)	4181.31 (3140.65-5262.28)	-41.52	123.99 (88.26-162.37)	119.48 (87.84-151.18)	-3.64
Guinea	1773.17 (1249.50-2335.22)	4055.41 (2884.34-5363.52)	128.71	36.90 (25.80-48.04)	35.54 (25.19-46.40)	-3.67
Qatar	522.99 (376.63-700.47)	3719.32 (2640.72-4932.59)	611.17	92.01 (66.28-121.21)	87.42 (63.03-116.31)	-4.99
Panama	2002.67 (1464.83-2575.12)	3635.22 (2764.65-4527.50)	81.52	81.47 (59.97-104.45)	82.51 (62.54-102.86)	1.27
Albania	3274.42 (2578.48-4029.94)	3518.12 (2754.23-4366.39)	7.44	93.43 (73.86-115.37)	125.50 (97.97-155.66)	34.32
Armenia	4537.44 (3210.88-5963.10)	3497.52 (2481.31-4456.25)	-22.92	124.44 (88.42-161.92)	113.38 (79.07-145.88)	-8.88
Congo	1220.95 (913.47-1555.51)	3445.73 (2565.08-4362.05)	182.22	60.99 (45.44-76.91)	66.07 (49.55-83.27)	8.33
Sierra Leone	1594.01 (1119.21-2116.50)	3392.21 (2354.65-4563.26)	112.81	43.88 (30.89-57.28)	40.61 (28.44-53.56)	-7.44
Slovenia	2338.03 (1919.11-2812.12)	3257.26 (2591.37-3889.53)	39.32	112.48 (92.10-135.21)	178.61 (141.47-215.11)	58.79
Togo	1061.98 (709.86-1428.65)	2922.36 (1994.24-3891.47)	175.18	36.25 (24.77-47.70)	37.80 (26.10-49.61)	4.27
Jamaica	2185.88 (1577.20-2877.00)	2826.66 (2071.66-3728.06)	29.31	90.87 (65.50-118.73)	89.88 (65.52-118.55)	-1.09
Central African Republic	1267.45 (904.32-1689.13)	2663.75 (1888.95-3418.12)	110.17	54.85 (39.36-72.30)	54.61 (38.63-69.96)	-0.42
Mauritius	1137.88 (784.64-1517.46)	2518.34 (2107.89-2946.89)	121.32	92.85 (64.41-122.79)	180.59 (150.11-213.30)	94.49
Namibia	1122.48 (822.20-1446.75)	2282.68 (1698.50-2860.56)	103.36	95.67 (71.45-122.29)	96.73 (73.34-119.31)	1.1
Cyprus	1194.97 (944.93-1456.25)	2217.97 (1744.52-2714.68)	85.61	146.07 (115.48-178.24)	152.61 (119.09-186.84)	4.48
Botswana	975.61 (709.95-1268.05)	2202.18 (1619.07-2787.48)	125.72	91.73 (67.41-117.34)	88.09 (65.96-110.86)	-3.96
Macedonia	1816.17 (1323.86-2335.92)	2195.84 (1648.89-2748.27)	20.91	86.68 (63.23-111.32)	96.50 (71.81-121.50)	11.32
Liberia	864.57 (604.63-1162.20)	2120.61 (1467.49-2848.20)	145.28	42.02 (29.64-55.62)	41.44 (29.48-55.64)	-1.38

Bosnia and Herzegovina	2841.73 (2076.68-3655.37)	1984.62 (1441.71-2515.53)	-30.16	57.23 (41.81-73.60)	62.65 (45.37-81.24)	9.47
Iceland	693.42 (566.24-822.63)	1807.94 (1533.53-2059.96)	160.73	255.14 (208.42-302.00)	510.21 (432.42-583.44)	99.98
Bahrain	586.96 (416.20-783.70)	1784.83 (1282.59-2292.82)	204.08	95.62 (68.03-126.16)	93.48 (67.05-120.03)	-2.24
Luxembourg	1127.39 (920.60-1323.82)	1758.74 (1444.38-2069.81)	56	271.05 (221.32-318.46)	261.12 (213.41-307.89)	-3.66
Lesotho	1032.88 (750.29-1337.91)	1732.15 (1290.72-2209.87)	67.7	85.11 (62.46-108.35)	96.68 (73.24-122.71)	13.59
Mauritania	739.75 (516.44-998.80)	1610.82 (1088.54-2167.79)	117.75	43.17 (30.31-57.31)	42.00 (28.27-55.52)	-2.7
Trinidad and Tobago	982.61 (686.03-1306.62)	1389.48 (1067.74-1723.71)	41.41	78.99 (55.07-104.10)	97.73 (73.59-122.05)	23.73
Gabon	569.23 (431.35-728.56)	1219.64 (911.89-1553.61)	114.26	67.43 (51.12-85.07)	69.92 (52.73-89.23)	3.7
Djibouti	246.05 (174.88-329.56)	1116.30 (767.52-1498.75)	353.68	71.07 (49.49-95.31)	84.04 (58.17-112.06)	18.25
Swaziland	582.41 (431.67-743.02)	1107.04 (834.41-1401.37)	90.08	94.50 (70.82-118.94)	100.77 (78.29-125.21)	6.64
Equatorial Guinea	185.02 (139.50-244.73)	1005.40 (741.03-1306.54)	443.4	55.41 (41.67-73.03)	68.49 (50.91-89.21)	23.62
Malta	530.93 (405.97-648.55)	936.71 (759.68-1105.19)	76.43	140.23 (107.69-171.52)	226.98 (182.96-268.43)	61.87
The Gambia	358.31 (244.23-478.33)	889.53 (619.98-1169.83)	148.26	43.41 (29.72-57.15)	40.46 (28.72-52.18)	-6.79
Timor-Leste	486.91 (330.07-668.73)	886.73 (602.48-1221.56)	82.11	63.17 (43.43-85.30)	63.34 (44.08-85.88)	0.26
Guinea-Bissau	304.48 (208.87-416.81)	684.09 (474.91-893.11)	124.68	37.94 (26.70-50.46)	36.97 (26.06-48.15)	-2.55
Brunei	395.47 (301.30-490.47)	605.09 (455.36-754.54)	53.01	141.63 (108.60-174.48)	112.67 (84.86-140.97)	-20.45
Comoros	234.83 (158.35-312.76)	603.98 (443.61-792.58)	157.2	68.76 (46.25-92.21)	82.95 (61.11-109.32)	20.63
Guyana	564.68 (397.01-751.06)	602.90 (435.33-781.16)	6.77	69.69 (49.25-92.12)	73.59 (53.67-95.06)	5.59
Fiji	561.15 (401.44-751.15)	598.11 (421.18-787.77)	6.59	71.86 (51.64-94.58)	64.06 (45.16-84.29)	-10.85
Suriname	316.41 (226.22-413.57)	590.78 (452.98-745.29)	86.71	78.46 (56.43-101.83)	100.07 (76.21-126.53)	27.55
Bhutan	336.62 (244.07-450.72)	546.60 (402.96-707.78)	62.38	60.85 (45.45-79.24)	65.05 (48.01-83.53)	6.9
Maldives	123.36 (85.09-170.14)	506.90 (364.03-660.97)	310.92	63.54 (45.07-85.07)	76.24 (53.87-98.94)	19.98
Solomon Islands	214.24 (151.91-282.47)	477.02 (347.46-626.13)	122.66	70.93 (51.46-92.02)	70.61 (51.45-91.58)	-0.45
Montenegro	471.51 (342.45-607.44)	438.90 (319.00-565.91)	-6.92	71.49 (51.99-92.41)	72.47 (52.23-94.27)	1.37
Kiribati	190.33 (150.94-237.75)	395.33 (297.04-521.03)	107.7	259.38 (209.52-319.63)	322.76 (242.47-419.61)	24.43
The Bahamas	262.90 (194.24-350.94)	388.35 (280.96-507.91)	47.72	91.26 (67.93-120.58)	92.01 (66.36-120.85)	0.82

Belize	145.49 (102.59-195.06)	383.31 (273.74-512.89)	163.47	82.20 (58.48-108.76)	80.53 (57.42-107.07)	-2.02
Cape Verde	134.38 (96.34-178.05)	279.72 (196.75-365.50)	108.15	45.49 (32.37-60.00)	44.78 (31.60-58.30)	-1.56
Barbados	248.35 (172.32-333.00)	260.87 (189.77-336.35)	5.04	89.93 (62.68-120.41)	89.49 (64.57-116.73)	-0.48
Vanuatu	94.38 (66.82-125.28)	206.60 (144.98-271.31)	118.91	67.97 (49.15-88.79)	66.53 (47.13-86.28)	-2.12
Greenland	191.09 (148.62-235.68)	197.90 (158.82-237.69)	3.56	282.91 (221.18-346.83)	332.53 (267.32-400.83)	17.54
Saint Lucia	126.57 (87.12-169.17)	166.59 (120.40-212.53)	31.62	90.66 (62.86-120.03)	87.99 (62.82-112.50)	-2.94
Samoa	124.29 (86.86-165.31)	161.02 (116.79-208.34)	29.55	77.45 (55.55-100.94)	78.49 (57.44-101.01)	1.35
Virgin Islands, U.S.	175.73 (141.96-212.47)	155.36 (121.42-203.39)	-11.59	164.08 (132.75-198.59)	221.66 (170.49-294.74)	35.09
Grenada	74.06 (53.59-97.72)	132.45 (101.03-166.23)	78.84	91.59 (66.71-120.04)	119.18 (91.04-149.39)	30.13
Guam	136.61 (100.97-176.14)	116.39 (82.25-151.97)	-14.81	87.71 (65.75-113.56)	73.34 (51.31-96.78)	-16.38
Sao Tome and Principe	45.42 (32.49-59.82)	112.56 (82.79-144.71)	147.81	47.81 (34.06-62.33)	53.84 (39.92-68.23)	12.62
Seychelles	67.44 (49.15-88.24)	111.45 (86.52-135.95)	65.25	87.45 (64.41-111.77)	100.58 (76.69-124.08)	15.01
Saint Vincent and the Grenadines	86.54 (58.60-115.23)	98.17 (71.41-127.80)	13.44	78.80 (54.27-104.76)	84.79 (61.41-111.00)	7.59
Andorra	65.66 (46.97-86.00)	87.30 (62.28-112.89)	32.96	99.48 (70.88-131.10)	105.00 (74.81-137.71)	5.55
Antigua and Barbuda	54.73 (38.67-72.11)	86.32 (64.25-109.92)	57.73	85.28 (60.35-112.26)	89.05 (66.09-113.69)	4.43
Federated States of Micronesia	74.52 (53.07-96.90)	84.20 (60.08-108.82)	12.99	80.86 (59.31-103.52)	77.86 (55.88-100.16)	-3.71
Dominica	69.32 (49.53-92.96)	77.72 (59.03-98.54)	12.13	94.29 (68.33-124.08)	110.45 (83.99-140.26)	17.14
Bermuda	76.27 (57.86-97.43)	76.93 (60.18-93.02)	0.86	110.85 (83.70-141.53)	126.52 (97.57-155.03)	14.14
Tonga	63.93 (44.67-84.78)	72.20 (51.42-95.27)	12.92	71.42 (50.64-94.16)	72.51 (51.89-95.98)	1.54
Saint Kitts and Nevis	35.10 (24.78-46.49)	54.81 (39.79-69.70)	56.16	84.88 (61.16-111.55)	85.06 (61.34-108.84)	0.22
Marshall Islands	30.53 (21.78-40.87)	45.43 (32.59-59.13)	48.8	77.78 (57.33-99.98)	76.99 (56.01-99.43)	-1.02
Northern Mariana Islands	47.18 (33.85-62.42)	40.65 (29.31-53.45)	-13.83	84.09 (61.15-109.94)	86.24 (61.67-113.89)	2.56
Monaco	34.26 (23.86-44.72)	40.46 (29.54-51.29)	18.09	116.23 (80.41-152.32)	128.07 (92.24-165.49)	10.18
American Samoa	36.17 (25.69-48.01)	36.96 (26.68-47.16)	2.16	74.42 (54.39-97.12)	78.45 (56.67-99.53)	5.41
San Marino	29.97 (21.15-39.63)	34.98 (25.20-45.11)	16.71	115.63 (81.32-153.89)	119.60 (86.43-156.97)	3.44
Palau	11.85 (8.17-16.04)	12.84 (8.77-16.74)	8.29	69.63 (48.11-92.92)	72.25 (48.71-95.44)	3.77

Cook Islands	13.47 (9.34-18.16)	12.33 (8.88-15.92)	-8.47	70.76 (49.12-94.02)	71.35 (50.48-91.88)	0.84
Tuvalu	6.81 (4.85-8.87)	9.52 (6.75-12.58)	39.74	74.80 (53.99-96.41)	74.55 (53.49-98.20)	-0.34
Nauru	7.65 (5.49-10.07)	8.61 (6.08-11.26)	12.67	79.19 (57.68-103.19)	76.29 (54.26-98.73)	-3.66
Niue	1.66 (1.21-2.14)	1.22 (0.88-1.58)	-26.54	79.20 (57.65-101.55)	75.32 (53.91-97.89)	-4.89
Tokelau	1.10 (0.80-1.46)	0.99 (0.71-1.27)	-9.92	76.42 (56.09-100.03)	74.47 (52.98-95.36)	-2.56

Table S10. Incidence, prevalence, deaths, DALYs, and their ASRs for opioid use disorders in 2021 and percentage changes from 1990 to 2021

	Incidence (95%UI)	Change (%)	ASIR (95%UI)	Change (%)	Prevalence (95%UI)	Change (%)	ASPR (95%UI)	Change (%)	Deaths (95%UI)	Change (%)	ASMR (95%UI)	Change (%)	DALYs (95%UI)	Change (%)	ASDR (95%UI)	Change (%)
Global	1942525.32 (1643342.29-2328363.17)	49.25	24.54 (20.74-29.48)	5.03	16164875.92 (14133119.75-18431509.71)	99.05	198.49 (173.42-227.22)	28.40	99555.46 (92947.93-108049.55)	139.51	1.19 (1.12-1.29)	39.12	11218518.58 (9188657.50-13159551.37)	107.17	137.15 (112.29-161.39)	32.27
Middle SDI	469928.34 (393636.59-562644.07)	4.25	18.82 (15.81-22.76)	-22.75	2832680.96 (2376556.56-3335824.12)	12.82	108.64 (90.53-128.61)	-26.96	11215.61 (9703.09-12660.87)	-33.12	0.42 (0.36-0.47)	-63.74	1667041.77 (1293206.12-2026943.33)	-10.95	63.60 (49.05-77.63)	-43.6
High SDI	609680.53 (518566.06-721842.39)	193.59	68.52 (57.67-82.33)	200.52	8316982.38 (7416372.86-9351503.51)	328	761.65 (674.95-864.14)	276.53	67688.86 (61163.60-75435.12)	665.43	5.47 (4.97-6.06)	503.4	6548594.74 (5421925.75-7567176.58)	421.26	587.41 (484.84-680.69)	348.93
Low-middle SDI	370910.63 (305808.87-455797.91)	94.24	18.22 (15.29-22.17)	6.67	2014727.50 (1651316.22-2416976.91)	104.82	102.41 (85.01-121.39)	6.73	7098.60 (6031.16-8161.99)	132.45	0.42 (0.36-0.48)	9.36	1140801.62 (861828.95-1406316.16)	109.26	58.69 (44.52-71.94)	8.3
High-middle SDI	335809.93 (284830.54-398898.71)	-14.05	27.16 (23.02-32.63)	-19.3	2208011.14 (1910876.26-2541927.15)	-6.9	159.87 (135.83-186.50)	-22.83	10285.21 (9480.06-10983.63)	-12.42	0.67 (0.62-0.71)	-37.47	1377430.08 (1108116.51-1649451.94)	-11.71	98.70 (78.06-119.48)	-27.81

Low SDI	155169.71 (124662.80-194320.30)	155.57	14.35 (11.93-17.42)	4.74	785959.83 (630138.16-959125.38)	155.87	80.76 (66.91-96.27)	5.16	3236.34 (2472.90-3940.78)	184.29	0.43 (0.34-0.51)	12.46	480610.28 (364315.24-591723.13)	168.74	50.81 (39.15-61.76)	10.07
Western Sub-Saharan Africa	49843.06 (39458.80-63152.49)	160.87	10.88 (8.95-13.31)	-1.46	253226.94 (198683.51-317066.54)	158.20	61.75 (49.74-74.58)	-1.66	118.80 (79.26-153.11)	66.94	0.05 (0.04-0.07)	-27.90	109727.47 (72681.28-147987.82)	154.95	26.84 (18.22-35.30)	-3.19
Western Europe	90781.79 (78907.06-104670.69)	7.88	24.07 (20.69-28.09)	11.32	1023831.48 (932726.71-1126292.27)	36.29	237.54 (213.94-263.02)	29.33	8949.90 (8426.08-9403.08)	103.72	1.67 (1.59-1.74)	57.13	778891.89 (657867.11-897778.66)	41.20	178.12 (149.99-207.30)	30.79
Tropical Latin America	37570.36 (29895.48-46843.20)	34.87	15.82 (12.43-19.74)	-7.40	225236.02 (177263.79-278465.73)	48.43	90.82 (71.67-113.42)	-9.07	86.82 (78.77-95.04)	655.7	0.03 (0.03-0.04)	261.40	95394.57 (63997.42-126549.21)	51.61	38.51 (25.72-51.57)	-6.74
Southern Sub-Saharan Africa	19234.08 (16063.75-23405.95)	11.96	23.31 (19.68-28.10)	-29.22	110212.87 (92590.14-129710.92)	16.67	134.96 (114.30-157.31)	-33.21	618.92 (548.57-703.66)	75.69	0.88 (0.78-0.99)	-4.50	71748.94 (57387.93-85223.33)	27.77	88.95 (71.72-105.11)	-27.01
Southern Latin America	12389.41 (9945.65-15253.04)	38.28	17.75 (14.11-22.10)	-0.70	80166.08 (63224.83-98118.46)	36.22	110.90 (86.57-136.22)	-7.81	107.19 (94.87-123.19)	370.45	0.14 (0.12-0.16)	185.28	37408.76 (26191.49-48810.64)	47.23	51.70 (36.07-67.75)	-0.42
Southeast Asia	68526.44 (56760.59-82426.84)	57.54	9.36 (7.79-11.23)	1.56	405625.59 (339421.19-477195.89)	75.99	54.30 (45.42-63.78)	3.15	1684.74 (1371.95-2077.11)	121.9	0.25 (0.20-0.30)	15.98	244550.83 (187103.57-300794.06)	81.90	32.90 (25.23-40.51)	7.04
South Asia	378428.49 (309714.62-466046.24)	116.24	18.90 (15.73-23.12)	13.05	2068682.48 (1684430.65-2497346.16)	127.42	105.86 (87.16-126.68)	13.27	8064.39 (6706.18-9346.32)	131.49	0.48 (0.40-0.55)	2.82	1190532.08 (891410.06-1478434.67)	124.04	61.71 (46.68-75.92)	10.76

Oceania	1737.37 (1407.02-2154.91)	126.42	12.49 (10.20-15.23)	3.57	9177.50 (7461.89-11119.92)	134.80	68.86 (56.55-82.59)	2.72	14.75 (10.45-20.08)	50.88	0.13 (0.09-0.17)	-33.27	4603.29 (3268.19-6103.50)	112.24	34.29 (24.39-44.64)	-6.37
North Africa and Middle East	245270.70 (203638.33-296380.08)	101.89	37.82 (31.50-45.62)	8.78	1456242.46 (1241717.57-1694858.65)	135.07	222.34 (190.30-258.56)	12.10	4903.39 (4140.26-5667.18)	142.6	0.81 (0.68-0.93)	1.29	842161.26 (650327.92-1031713.65)	133.42	128.78 (99.59-157.46)	8.82
High-income North America	456336.88 (382679.81-549886.38)	425.34	144.24 (120.13-174.95)	377.88	6894161.34 (6086133.84-7821274.98)	577.95	1890.26 (1659.84-2156.24)	477.38	58205.46 (51549.04-65872.21)	1179.33	14.50 (12.92-16.30)	914.00	5570171.42 (4605348.50-6442015.38)	759.15	1502.44 (1235.96-1740.10)	623.56
High-income Asia Pacific	23884.50 (19434.42-28719.24)	-14.64	14.92 (12.05-18.42)	-1.47	162536.23 (132441.29-194090.55)	-8.58	90.22 (71.08-109.56)	-4.81	293.23 (270.14-314.18)	109	0.12 (0.11-0.13)	68.96	78890.36 (55977.92-100982.85)	-2.56	43.74 (30.90-57.08)	1.30
Eastern Sub-Saharan Africa	42754.96 (34331.90-53621.37)	145.90	10.77 (8.94-12.99)	0.27	214835.72 (171817.08-262360.99)	147.83	60.32 (49.34-71.65)	0.27	1785.43 (1210.58-2304.85)	224.56	0.62 (0.43-0.79)	11.90	177705.83 (136840.71-219976.63)	187.39	51.62 (40.04-62.98)	11.95
Eastern Europe	129174.80 (110164-153540.37)	-15.56	73.32 (61.90-87.26)	5.34	890549.07 (787614.12-1008612.34)	-5.83	431.53 (379.31-493.25)	9.05	6016.21 (5461.80-6599.42)	35.15	2.55 (2.33-2.81)	43.48	657684.54 (555403.19-766235.56)	9.47	311.15 (259.01-365.83)	24.09
East Asia	244997.87 (202875.87-293576.86)	-41.65	16.71 (13.88-20.27)	-44.89	1533265.15 (1278054.78-1804685.35)	-37.12	94.72 (77.62-112.73)	-49.93	6019.50 (4808.14-7354.37)	-68.84	0.33 (0.26-0.40)	-80.43	887517.67 (681703.67-1086677.91)	-54.70	54.37 (40.97-67.47)	-64.58
Central Sub-Saharan Africa	16045.17 (12747.85-20234.11)	187.10	12.49 (10.24-15.37)	9.45	81131.58 (64601.38-100052.60)	189.33	69.81 (56.86-83.82)	9.50	360.22 (203.28-548.06)	225.17	0.35 (0.20-0.53)	18.15	52140.46 (37993.80-67999.35)	205.18	45.14 (32.46-58.02)	14.45

Central Latin America	40991.33 (32758.11-50724.74)	48.75	15.25 (12.19-18.85)	-5.01	235672.24 (186457.35-288977.95)	63.64	87.65 (69.37-107.42)	-6.19	238.67 (206.02-277.16)	91.01	0.09 (0.08-0.10)	-11.96	108440.22 (75531.51-142294.78)	64.26	40.33 (28.12-52.90)	-6.54
Central Europe	16499.13 (14140.58-19429.49)	-0.09	16.10 (13.62-18.96)	21.27	102510.59 (89594.96-116807.56)	8.69	89.23 (77.04-104.10)	21.83	778.56 (715.05-845.84)	30.17	0.56 (0.52-0.61)	25.03	74952.81 (62010.21-87477.29)	10.76	64.41 (53.16-75.80)	22.81
Central Asia	35075.15 (29584.40-41820.03)	35.38	36.68 (30.96-43.70)	0.93	213900.74 (186052.08-247374.62)	51.18	213.65 (185.05-247.50)	2.42	603.36 (496.88-709.82)	169.60	0.63 (0.52-0.74)	66.66	117883.36 (89535.47-144813)	67.57	117.72 (89.49-144.38)	12.61
Caribbean	7626.92 (6095.73-9436.55)	9.77	15.61 (12.48-19.26)	-13.58	43793.37 (35073.62-53682.11)	13.29	88.28 (70.52-108.35)	-18.84	46.37 (36.67-56.21)	108.63	0.09 (0.07-0.11)	28.31	20232.02 (14015.11-26534.75)	17.74	40.77 (28.15-53.52)	-15.93
Australasia	12977.25 (11088.88-15009.39)	40.75	44.87 (38.68-51.99)	3.34	89881.68 (82239.96-98308.74)	59.58	284.21 (259.19-311.84)	10.15	582.26 (507.54-669.12)	75.68	1.74 (1.51-1.99)	14.50	65324.68 (53569.67-76293.57)	54.20	205.48 (168.06-240.26)	5.71
Andean Latin America	12379.64 (9822.74-15470.69)	84.37	17.39 (13.80-21.67)	2.27	70236.78 (54745.32-86630.89)	102.21	99.23 (77.66-122.43)	1.92	77.29 (61.20-100.95)	268.73	0.12 (0.09-0.16)	56.06	32556.12 (22899.62-43838.92)	110.14	46.21 (32.59-61.99)	5.76

Table S11. Incidence, Prevalence, Deaths, DALYs and their ASRs for amphetamine use disorders in 2021 and percentage changes from 1990 to 2021

	Incidence (95%UI)	Change (%)	ASIR (95%UI)	Change (%)	Prevalence (95%UI)	Change (%)	ASPR (95%UI)	Change (%)	Deaths (95%UI)	Change (%)	ASMR (95%UI)	Change (%)	DALYs (95%UI)	Change (%)	ASDR (95%UI)	Change (%)
Global	1069011.05 (1482513.97-757796.92)	-21.65	13.72 (9.70-19.07)	-39.56	9172894.90 (6702516.23-12083457.81)	-15.41	115.99 (84.63-153.55)	-37.92	9879.87 (8919.92-11061.27)	103.61	0.12 (0.11-0.13)	27.98	1677366.57 (1171027.18-2343857.57)	-1.64	20.98 (14.56-29.33)	-29.18
Middle SDI	393277.57	-39.79	16.65	-45.46	3501223.91	-32.78	142.22	-45.19	2600.71	-11.31	0.10	-43.2	588140.05	-31.27	23.63 (15.53-	-45.55

	(277522.45-551726.87)		(11.67-23.46)		(2542487.27-4671305.46)		(102.85-192.42)		(2102.31-3112.42)		(0.08-0.12)		(389598.11-847276.42)		34.41)	
High SDI	269459.33 (194652.84-362302.87)	17.84	30.22 (21.39-41.49)	17.78	2295881.42 (1730438.75-2926049.57)	28.2	237.37 (177.48-305.30)	23.24	5584.46 (5068.59-6575.82)	1217.3	0.44 (0.41-0.52)	910.92	557965.21 (436124.14-711934.20)	116.66	52.96 (40.45-69.35)	91.55
Low-middle SDI	111604.28 (76809.72-158241.23)	56.68	5.28 (3.66-7.46)	-9.2	849567.70 (588997.34-1176014.52)	62.54	40.67 (28.60-55.91)	-9.4	281.58 (239.05-373.17)	178.72	0.02 (0.01-0.02)	41.23	127282.27 (77033.94-189721.04)	70.19	6.15 (3.75-9.10)	-5.15
High-middle SDI	244575.81 (172533.77-341002.29)	-37.42	22.78 (15.75-32.04)	-29.88	2182468.45 (1615165.36-2883241.10)	31.4	185.54 (133.58-250.49)	-29.82	1346.29 (1204.05-1520.20)	-1.85	0.09 (0.08-0.11)	-22.61	354761.50 (236125.52-519899.90)	-28.69	29.48 (18.97-43.15)	-29.06
Low SDI	49205.41 (32654.14-70375.58)	150.3	4.17 (2.85-5.84)	2.49	336500.53 (226267.89-481724.80)	150.16	30.09 (21.03-42.28)	2.63	64.20 (46.20-122.05)	181.75	0.01 (0.01-0.01)	14.49	48125.67 (27512.10-74117.01)	154.16	4.36 (2.56-6.63)	4.47
Western Sub-Saharan Africa	23350.27 (15277.66-33394.29)	159.17	4.66 (3.16-6.60)	-2.88	159851.39 (106779.34-230265.22)	155.35	33.73 (23.57-47.81)	-3.68	4.51 (2.54-7.52)	474.92	0.00 (0.00-0.00)	103.93	21579.73 (11726.76-34496.18)	159.23	4.55 (2.54-7.32)	-2.41
Western Europe	86604.46 (60432.91-119219.80)	-2.93	25.31 (17.31-35.24)	8.78	691670.26 (511029.02-911455.11)	4.14	187.82 (135.17-251.47)	12.74	594.96 (546.92–632.45)	376.04	0.14 (0.13-0.15)	349.02	120761.16 (83029.70-170592.71)	28.79	32.15 (21.68-45.88)	36.64
Tropical Latin America	44109.58 (29562.95-62514.62)	26.63	19.40 (12.91-27.72)	-2.77	416441.73 (291501.94-567028.75)	35.84	174.84 (121.64-239.06)	-4.82	24.22 (20.47–26.76)	429.9	0.01 (0.01-0.01)	225.24	55392.40 (31740.53-85992.10)	37.84	23.26 (13.32-36.42)	-3.32
Southern Sub-Saharan Africa	10025.69 (6950.06-14005.89)	37.91	11.74 (8.21-16.33)	-7.28	98764.20 (69327.70-133499.03)	45.46	113.54 (80.78-152.22)	-11.74	48.82 (37.76–61.12)	261.01	0.07 (0.05-0.08)	104.04	14996.54 (9560.91-21632.14)	56.76	17.50 (11.31-24.89)	-4.33

Southern Latin America	7327.63 (4965.41-10307.59)	35.81	10.73 (7.23-15.12)	0.98	50716.44 (35087.07-69595.62)	42.39	72.35 (49.93-99.31)	1.67	9.45 (7.51–11.17)	1066.8	0.01 (0.01-0.01)	625.32	7084.26 (4207.91-11209.69)	50.54	10.07 (5.93-15.92)	7.08
Southeast Asia	163201.96 (109538.47-236146.72)	27.87	22.65 (15.16-32.80)	-3.20	1400321.53 (965409.04-1934874.06)	35.76	189.63 (130.40-262.39)	-4.57	247.20 (170.63–347.38)	463.81	0.03 (0.02-0.05)	189.98	198299.60 (116046.93-306865.28)	42.88	26.84 (15.71-41.66)	0.11
South Asia	34755.44 (23974.61-48952.14)	100.13	1.73 (1.21-2.43)	4.01	233077.26 (162876.01-321166.46)	105.96	11.62 (8.13-15.85)	5.40	310.20 (244.33–439.39)	229.86	0.02 (0.01-0.02)	59.15	47242.19 (31994.33-66992.40)	129.36	2.39 (1.63-3.36)	18.16
Oceania	2612.98 (1701.09-3829.01)	105.95	17.24 (11.45-25)	0.12	20312.13 (13586.70-28439.92)	111.33	136.57 (92.19-189.06)	-0.98	0.94 (0.63–1.80)	265.43	0.01 (0.01-0.02)	48.36	2733.43 (1478.29-4487.31)	114.84	18.44 (10.09-30.05)	0.62
North Africa and Middle East	30940.62 (21502.60-43477.06)	92.62	4.75 (3.30-6.67)	6.78	245067.88 (174873.12-333079.05)	112.79	37.14 (26.45-50.55)	9.93	256.71 (177.79-362.25)	312.42	0.04 (0.03-0.06)	71.88	44898.96 (29573.69-62268.52)	144.27	6.83 (4.51-9.43)	22.27
High-income North America	134154.29 (96486.60-180863.55)	50.95	42.00 (29.89-56.72)	33.28	1115781.71 (842665.08-1429137.48)	65.80	334.25 (249.54-432.38)	48.87	4764.19 (4231.08–5795.52)	2267.3	1.15 (1.03-1.39)	1687.86	361323.06 (295662.78-445786.90)	267.44	98.53 (79.65-122.87)	201.54
High-income Asia Pacific	21213.42 (14724.40-29587.10)	-24.07	15.15 (10.11-21.23)	-2.05	162801.35 (114718.82-221266.41)	-20.41	110.32 (76.10-153.56)	-2.89	23.60 (21.87–25.52)	339.8	0.01 (0.01-0.01)	296.65	22674.11 (13620.26-34979.55)	-17.13	15.20 (8.70-23.86)	0.09
Eastern Sub-Saharan Africa	22939.45 (14930.45-33013.19)	147.02	4.98 (3.36-7.00)	0.06	157639.41 (105432.70-226422.81)	147.82	36.30 (25.41-51.02)	-0.12	27.35 (17.04–64.03)	386.69	0.01 (0.01-0.02)	97.04	22341.99 (12701.36-34402.52)	157.22	5.24 (3.06-8.03)	4.46
Eastern Europe	40586.03 (29385.00-	-22.64	26.25 (18.74-	5.43	346165.99 (266197.84-	-18.31	203.57 (153.65-	7.54	462.17 (402.69–	201.63	0.22 (0.20-	238.86	70582.56 (52501.44-94339.57)	9.78	39.53 (28.60-53.32)	37.84

	54218.22)		35.39)		441437.79)		264.85)		511.51)		0.25)					
East Asia	360476.41 (255958.55-500266.35)	-55.35	32.31 (22.40-45.25)	-36.19	3361886.46 (2502702.12-4421368.16)	-48.59	269.62 (195.03-362.11)	-37.16	2733.56 (2199.81-3331.29)	-32.93	0.17 (0.14-0.21)	-45.55	574681.33 (386256.01-831811.12)	-47.63	44.67 (29.64-65.46)	-39.09
Central Sub-Saharan Africa	7572.62 (4982.17-10714.86)	163.43	5.31 (3.64-7.43)	1.44	52362.58 (34986.09-74638.54)	163.96	38.70 (26.71-54.28)	1.69	6.15 (3.19–14.33)	559.42	0.01 (0.00-0.01)	155.73	7256.92 (3989.96-11452.57)	175.15	5.41 (3.00-8.36)	6.34
Central Latin America	18139.04 (12330.22-25214.61)	47.11	6.75 (4.59-9.38)	0.45	135833.68 (94553.25-186853.53)	55.63	50.38 (35.05-69.23)	-0.16	111.22 (88.33–130.56)	424.1	0.04 (0.03-0.05)	208.23	23612.87 (15425.90-33765.90)	84.33	8.76 (5.72-12.52)	17.97
Central Europe	20754.02 (14565.27-28716.06)	-16.28	23.83 (16.19-33.54)	14.56	174621.24 (126966.67-231212.08)	-7.35	181.72 (129.43-246.17)	17.05	52.29 (46.69–57.89)	111.4	0.05 (0.04-0.05)	145.37	25907.36 (16326.99-39168.50)	-1.11	26.84 (16.42-40.57)	24.43
Central Asia	15659.15 (10909.41-21926.13)	33.08	16.61 (11.63-23.13)	5.57	126307.45 (90686.30-169093.91)	46.83	127.60 (91.05-172.64)	9.03	70.49 (56.28–89.29)	694.59	0.07 (0.06-0.09)	374.89	20080.86 (12922.32-29012.74)	70.46	20.19 (12.99-29.11)	25.26
Caribbean	3361.62 (2331.66-4741.16)	25.50	6.95 (4.79-9.85)	3.97	25091.91 (17637.31-34498.39)	30.76	51.23 (35.87-70.57)	3.44	18.35 (14.07–22.60)	834.92	0.04 (0.03-0.04)	508.42	4181.65 (2652.26-6159.35)	58.35	8.49 (5.37-12.56)	23.87
Australasia	14589.80 (9985.08-20499.25)	18.64	55.68 (38.08-78.43)	-4.91	148282.99 (108709.31-194960.58)	38.66	513.42 (371.55-682.47)	3.61	86.22 (72.60–99.30)	1726.3	0.27 (0.23-0.31)	1163.76	23837.98 (15810.33-34074.88)	68.70	81.67 (53.61-117.56)	24.73
Andean Latin America	6636.56 (4509.29-9426.71)	80.65	9.29 (6.33-13.13)	5.76	49897.29 (34288.26-69286.54)	94.22	68.86 (47.60-95.21)	6.14	27.25 (20.57–36.27)	597.38	0.04 (0.03-0.05)	257.72	7897.61 (4878.23-11789.78)	118.58	11.02 (6.90-16.44)	20.09

Table S12. Incidence, Prevalence, Deaths, DALYs, and their ASRs for cannabis use disorders in 2021 and percentage changes from 1990 to 2021

	Incidence (95%UI)	Change (%)	ASIR (95%UI)	Change (%)	Prevalence (95%UI)	Change (%)	ASPR (95%UI)	Change (%)	Deaths (95%UI)	Change (%)	ASMR (95%UI)	Change (%)	DALYs (95%UI)	Change (%)	ASDR (95%UI)	Change (%)
Global	3625507.41 (2738090.17-4737132.26)	27.22	46.77 (35.25-61.17)	-3.49	22639631.67 (17652673.71-30335210.55)	33.03	286.23 (222.58-384.31)	-4.18	no data	no data	no data	no data	653848.54 (389510.18-1017565.33)	32.9	8.27 (4.90-12.86)	-4.17
Middle SDI	1019872.54 (764481.69-1336829.54)	30.61	42.89 (31.99-56.76)	10.72	6412252.21 (4989963.34-8547117.48)	46.24	258.47 (198.74-350.17)	12.7	no data	no data	no data	no data	185665.55 (109868.43-289618.26)	46.12	7.49 (4.41-11.73)	12.84
High SDI	859562.34 (662900.46-1090967.18)	-2.17	106.41 (80.40-136.64)	-1.46	6027323.23 (4755966.73-7730721.27)	1.17	655.96 (511.77-852.96)	-2.31	no data	no data	no data	no data	173222.80 (106687.04-264113.14)	0.69	18.89 (11.53-28.83)	-2.64
Low-middle SDI	824188.53 (618668.26-1106024.45)	63.94	38.86 (29.16-51.76)	-3.63	4697927.25 (3565098.27-6470399.81)	73.94	227.75 (174.78-308.98)	-3.8	no data	no data	no data	no data	135460.09 (79054.45-215589.92)	74.11	6.56 (3.88-10.40)	-3.58
High-middle SDI	472414.86 (365966.98-603616.18)	-4.04	44.92 (34.37-57.91)	4.22	3085894.55 (2487155.35-3954551.99)	5.12	264.53 (208.24-342.36)	5.45	no data	no data	no data	no data	89656.16 (54528.79-133534.17)	5.2	7.69 (4.66-11.79)	5.66
Low SDI	446113.02 (312979.23-614513.33)	132.34	35.15 (25.65-47.64)	-3.6	2394593.86 (1703321.46-3442479.60)	134.9	209.94 (155.68-290.75)	-3.18	no data	no data	no data	no data	69217.49 (39096.28-113009.83)	136.2	6.06 (3.54-9.54)	-2.65
Western Sub-Saharan Africa	116242.93 (82564.91-160552.90)	173.76	21.72 (15.81-29.29)	1.56	598225.61 (420595.33-868711.89)	170.83	124.85 (91.97-173.12)	1.34	no data	no data	no data	no data	17401.64 (9777.88-28521.28)	172.82	3.63 (2.08-5.73)	2.02

Western Europe	296650.20 (238105.63-365090.75)	-14.96	96.20 (76.54-119.49)	-8.38	2052327.60 (1711692.43-2514747.52)	-10.76	598.62 (494.10-743.63)	-3.45	no data	no data	no data	no data	59284.93 (38329.41-87470.68)	-10.89	17.31 (11.24-25.56)	-3.53
Tropical Latin America	139924.32 (105960.53-180856.54)	7.84	64.81 (48.52-84.57)	-11.26	980118.21 (735128.73-1307247.29)	21.13	421.92 (314.68-564.89)	-12.93	no data	no data	no data	no data	28082.77 (17026.64-43786.04)	20.69	12.10 (7.27-19.12)	-12.98
Southern Sub-Saharan Africa	40078.34 (28056.57-55104.14)	59.67	46.14 (32.43-63.24)	12.66	251053.30 (176417.83-358192.62)	76.63	291.16 (205.32-413.03)	15.38	no data	no data	no data	no data	7234.22 (4044.59-11724.71)	75.44	8.39 (4.76-13.56)	14.81
Southern Latin America	37312.56 (30469.83-45350.60)	44.05	59.35 (48.47-72.21)	19.22	247682.34 (218245.15-285763.73)	67.71	369.24 (325.22-426.04)	27.61	no data	no data	no data	no data	7150.85 (4794.62-10423.83)	67.01	10.67 (7.19-15.54)	27.17
Southeast Asia	330569.09 (243203.92-449144.70)	39.25	46.30 (33.74-63.67)	4.64	1985877.05 (1451670.51-2793634.71)	52.61	269.94 (194.27-381.28)	4.57	no data	no data	no data	no data	57712.21 (33184.25-92541.81)	52.79	7.85 (4.52-12.65)	4.88
South Asia	921896.64 (671942.01-1243197.50)	76.05	44.47 (32.43-59.54)	-1.40	5287616.79 (4007906.25-7133128.43)	86.22	260.40 (198.16-346.85)	-1.61	no data	no data	no data	no data	152019.00 (90185.70-245155.89)	86.35	7.48 (4.47-11.97)	-1.41
Oceania	11114.51 (7189.98-15898.89)	101.39	72.23 (47.14-102.46)	0.88	67710.99 (43403.96-101998.73)	110.78	455.60 (298.54-672.23)	0.78	no data	no data	no data	no data	1960.07 (1041.95-3354.10)	110.73	13.18 (7.15-22.17)	0.87
North Africa and Middle East	148859.63 (105670.95-205530.10)	90.47	22.73 (16.10-31.39)	11.17	874292.51 (614895.56-1226849.29)	112.69	133.59 (93.62-187.44)	13.68	no data	no data	no data	no data	25513.92 (14844.12-40835.20)	112.95	3.90 (2.27-6.24)	13.77

High-income North America	453989.25 (343610.30-580296.80)	12.29	151.30 (114.29-196.40)	-5.07	3203119.58 (2506547.24-4146498.57)	10.90	973.88 (752.87-1275.29)	-4.95	no data	no data	no data	no data	91512.76 (55642.31-140439.51)	10.04	27.88 (16.97-42.78)	-5.59
High-income Asia Pacific	97833.29 (71174.99-132375.41)	-31.24	78.59 (55.32-109.81)	-1.22	671712.18 (500134.03-931657.57)	-21.88	466.09 (328.88-669.92)	-1.20	no data	no data	no data	no data	19468.47 (11263.67-31469.65)	-21.95	13.53 (7.70-22.38)	-1.14
Eastern Sub-Saharan Africa	186404.35 (125188.85-268405.72)	136.23	36.35 (25.69-50.62)	-1.02	998842.32 (664372.63-1470597.08)	140.22	216.64 (152.35-307.84)	-1.77	no data	no data	no data	no data	28941.15 (15637.27-48027.02)	141.77	6.27 (3.62-10.11)	-1.11
Eastern Europe	84503.59 (59783.33-115642.66)	-20.72	55.89 (37.85-79.13)	6.21	556058.38 (401315.96-756841.18)	-18.95	336.82 (230.68-483.34)	6.01	no data	no data	no data	no data	16108.61 (9335.8-25285.02)	-18.82	9.78 (5.45-15.57)	6.25
East Asia	435242.57 (321483.11-570773.19)	-0.14	36.16 (26.09-49.11)	21.95	2786338.91 (2136491.81-3697617.65)	17.31	205.90 (154.31-284.04)	23.61	no data	no data	no data	no data	81299.11 (47622.61-124806.45)	17.45	6.02 (3.44-9.65)	23.98
Central Sub-Saharan Africa	47455.35 (31794.14-70628.45)	162.51	30.72 (21.35-43.62)	0.29	250152.55 (163268.89-387539.48)	162.80	180.66 (124.03-266.94)	0.45	no data	no data	no data	no data	7220.77 (3830.18-12315.81)	166.18	5.21 (2.80-8.54)	1.71
Central Latin America	111124.44 (85701.91-142883.51)	61.09	42.34 (32.65-54.46)	17.41	736482.23 (592142.12-929124.77)	89.67	274.83 (220.77-346.77)	23.88	no data	no data	no data	no data	21323.52 (13293.22-31776.50)	89.03	7.96 (4.96-11.83)	23.73
Central Europe	44855.81 (35084.16-56457.42)	-36.18	55.12 (42.33-71.28)	-6.11	310649.88 (249056.16-391732.19)	-28.02	342.62 (264.57-440.97)	-3.86	no data	no data	no data	no data	9036.94 (5660.15-13633.34)	-27.95	9.98 (6.13-15.25)	-3.72

Central Asia	30893.35 (20961.56-45407.52)	24.60	33.79 (22.62-50.51)	3.01	187930.48 (125282.18-282767.76)	33.75	198.04 (129.47-302.45)	3.61	no data	no data	no data	no data	5477.07 (2895.86-9110.35)	34.05	5.78 (3.01-9.70)	3.91
Caribbean	34582.19 (23028.14-49380.18)	20.83	74.97 (49.43-108.44)	6.02	228897.72 (153560.32-332134.40)	28.67	476.43 (315.55-696.65)	5.20	no data	no data	no data	no data	6595.39 (3621.57-10842.93)	28.32	13.73 (7.44-22.62)	5.07
Australasia	28370.79 (22417.85-35637.59)	-22.24	114.65 (90.13-144.62)	-37.44	197336.84 (165507.29-236191.74)	-23.57	718.11 (599.67-860.36)	-41.41	no data	no data	no data	no data	5665.37 (3695.89-8515.56)	-23.56	20.65 (13.45-31.16)	-41.32
Andean Latin America	27604.19 (20105.31-36916.12)	55.94	39.76 (29.10-52.90)	-2.21	167206.21 (122190.01-232741.50)	71.45	236.64 (172.76-327.74)	-2.83	no data	no data	no data	no data	4839.74 (2833.38-7766.98)	70.78	6.85 (4.03-10.91)	-3.12

Table S13. Incidence, Prevalence, Deaths, DALYs and their ASRs for cocaine use disorders in 2021 and percentage changes from 1990 to 2021

	**Incidence (95%UI)**	**Change (%)**	**ASIR (95%UI)**	**Change (%)**	**Prevalence (95%UI)**	**Change (%)**	**ASPR (95%UI)**	**Change (%)**	**Deaths (95%UI)**	**Change (%)**	**ASMR (95%UI)**	**Change (%)**	**DALYs (95%UI)**	**Change (%)**	**ASDR (95%UI)**	**Change (%)**
Global	219145.41 (158098.10-298956.60)	17.5	2.87 (2.06-3.93)	-7.32	4073254.62 (3203308.08-5120095.80)	37.55	50.63 (39.74-63.79)	-7.35	12555.16 (11405.88-14508.40)	254.88	0.15 (0.14-0.17)	107.5	1133623.69 (917462.38-1427944.23)	94.18	13.88 (11.18-17.52)	27.2
Middle SDI	53204.16 (36484.17-75212.15)	19.45	2.33 (1.59-3.33)	10.79	812008.33 (618549.16-1070924.69)	60.89	32.48 (24.64-43.22)	18.6	2355.48 (2138.99-2606.83)	115.55	0.09 (0.08-0.10)	22.57	224861.57 (177672.92-284275.44)	78.41	8.82 (6.96-11.21)	23.83
High SDI	102712.56 (76248.81-141967.45)	13.85	13.52 (10.02-18.91)	19.87	2359632.44 (1872150.35-2886933.97)	28.88	228.90 (179.18-285.81)	15.97	7958.45 (7162.63-9580.21)	659.42	0.62 (0.56-0.73)	474.26	675910.38 (552476.41-853433.09)	123.4	60.5 (48.28-77.26)	86.62
Low-middle	23448.82 (15824.97-	75.03	1.10 (0.75-	6.96	292121.90	117.52	14.55 (11.04-	19.55	1328.84	187.31	0.07	37.94	104352.10	150.64	5.33 (4.09-6.78)	32.51

SDI	32196.06)		1.49)		(218751.25-387669.11)		19.07)		(1006.28-1708.35)		(0.06-0.10)		(80380.90-132399.74)			
High-middle SDI	32042.97 (22233.23-44566.38)	-8.98	3.29 (2.23-4.73)	7.53	538615.23 (411642.02-697651.36)	16.32	42.67 (31.64-56.57)	5.76	631.40 (573.35-692.47)	-20.58	0.04 (0.04-0.05)	-42.14	103573.69 (73936.08-143209.96)	1.07	7.96 (5.57-11.14)	-11.43
Low SDI	7536.19 (5092.69-10588.19)	156.52	0.63 (0.45-0.89)	7.29	67875.98 (50646.32-89782.21)	163.6	6.86 (5.34-8.62)	10.12	275.54 (139.16-494.94)	101.67	0.04 (0.02-0.06)	-19.28	24252.05 (15665.50-37947.68)	129.18	2.62 (1.68-4.07)	-6.41
Western Sub-Saharan Africa	2853.83 (2001.34-3942.60)	212.08	0.70 (0.53-0.90)	24.08	24614.14 (19154.43-31391.73)	204.29	7.80 (6.52-9.37)	30.43	5.83 (1.65–9.48)	130.54	0.00 (0.00-0.00)	-19.93	4325.14 (2932.57-6196.79)	172.09	1.27 (0.87-1.81)	12.91
Western Europe	29067.80 (19503.20-42953.54)	-4.51	9.53 (6.20-14.54)	5.48	563854.78 (424299.71-757969.63)	10.58	138.33 (100.60-190.17)	8.60	510.64 (489.52–540.54)	96.66	0.11 (0.11-0.12)	79.34	100910.53 (69960.10-139400.93)	21.65	24.70 (16.67-35.05)	19.15
Tropical Latin America	24310.22 (16549.49-36226.57)	68.09	12.04 (8.21-18.04)	53.10	462729.37 (345222.11-603899.38)	122.89	195.30 (144.63-256.82)	47.51	1047.40 (977.69–1128.21)	1015.2	0.42 (0.40-0.46)	547.23	117508.65 (92242.28-150144.51)	252.67	49.14 (38.40-63.14)	131.82
Southern Sub-Saharan Africa	4691.37 (3311.29-6582.77)	54.76	5.51 (3.89-7.67)	7.26	72549.46 (54375.11-94965.67)	101.00	89.26 (67.95-114.91)	14.18	96.70 (74.84–117.37)	89.18	0.14 (0.11-0.17)	1.00	13830.97 (9845.47-18884.50)	87.18	17.28 (12.43-23.48)	4.99
Southern Latin America	9046.29 (5996.63-13795.19)	19.78	14.82 (9.74-22.82)	4.26	176560.92 (134233.96-233150.54)	51.38	250.22 (188.83-332.21)	6.85	21.71 (18.74–25.84)	412.62	0.03 (0.02-0.03)	202.66	24741.16 (14934.32-37543.51)	53.78	35.08 (21.05-53.62)	8.66
Southeast	1699.02 (1044.34-	17.69	0.24 (0.14-	-12.33	14640.17	31.76	1.99 (1.22-	-12.74	150.86 (113.52–	143.4	0.02	15.94	8415.91	90.09	1.13 (0.84-1.66)	8.38

Asia	2601.75)		0.36)		(8907.41-21594.31)		2.92)		229.10)		(0.02-0.03)		(6210.55-12339.41)			
South Asia	7534.18 (4912.50-10938.63)	79.28	0.37 (0.24-0.54)	-1.75	62374.03 (42168.31-88673.90)	92.90	3.18 (2.19-4.42)	-1.38	1057.82 (699.96–1500.10)	138.84	0.06 (0.04-0.09)	10.51	54579.76 (37892.94-75331.70)	110.50	2.91 (2.02-4.03)	5.42
Oceania	42.03 (24.11-63.51)	93.12	0.28 (0.16-0.42)	-6.06	333.55 (191.54-495.88)	103.16	2.30 (1.35-3.42)	-6.97	1.48 (0.94–2.53)	84.8	0.01 (0.01-0.02)	-18.20	124.94 (87.66-193.30)	85.92	0.92 (0.65-1.41)	-16.51
North Africa and Middle East	10523.34 (6971.10-15194.67)	61.87	1.62 (1.07-2.34)	-3.60	112383.26 (79764.45-150781.99)	95.52	17.26 (12.39-23.08)	-1.88	654.25 (498.07–847.75)	100.02	0.11 (0.08-0.14)	-22.09	46010.38 (35312.84-60136.29)	95.76	7.17 (5.50-9.34)	-13.55
High-income North America	69310.59 (51403.30-97643.89)	29.01	23.87 (17.69-33.93)	8.38	1695549.51 (1350180.89-2073361.42)	39.26	479.97 (379.72-592.54)	16.82	7384.56 (6591.84-8993.36)	899.78	1.75 (1.57-2.11)	651.12	558103.21 (464194.95-689281.48)	174.87	147.83 (121.82-183.99)	118.06
High-income Asia Pacific	7766.37 (5368.56-10825.76)	-36.00	6.50 (4.43-9.50)	-3.81	177748.44 (133915.05-235128.80)	-9.59	101.46 (72.92-139.47)	-3.23	39.67 (36.28–44.69)	105.99	0.02 (0.02-0.02)	73.71	25844.28 (16005.38-39579.79)	-7.34	14.74 (8.79-23.21)	-0.92
Eastern Sub-Saharan Africa	2139.22 (1442.15-3120.34)	178.10	0.48 (0.34-0.68)	14.10	18276.21 (13537.54-24063.30)	182.85	5.17 (4.11-6.43)	15.07	85.77 (32.71–223.64)	117.62	0.03 (0.01-0.07)	-14.55	7914.19 (4900.41-14130.12)	139.53	2.35 (1.45-4.26)	-4.24
Eastern Europe	6373.89 (4516.28-8787.37)	-18.97	4.35 (3.13-6.05)	12.61	88303.19 (67796.70-110414.75)	-5.51	48.53 (36.71-62.33)	17.64	280.99 (253.57–311.75)	-48.16	0.12 (0.11-0.13)	-43.37	26056.54 (21115.93-32795.73)	-32.81	13.06 (10.35-16.89)	-19.58
East Asia	8358.49 (5471.72-	-37.39	0.72 (0.46-	-18.39	85080.34	-21.63	6.13 (4.18-	-20.06	202.39 (113.45–	-56.73	0.01	-69.48	22211.93	-43.86	1.51 (0.99-2.13)	-48.87

	12305.02)		1.05)		(58771.69-116626.32)		8.59)		277.37)		(0.01-0.02)		(14769.89-30682.20)			
Central Sub-Saharan Africa	1180.26 (772.91-1675.24)	190.04	0.80 (0.54-1.14)	11.07	9700.56 (6925.54-13216.39)	193.78	7.95 (5.88-10.39)	11.57	18.88 (5.08–49.87)	217.58	0.02 (0.00-0.05)	20.60	2621.89 (1689.31-4160.48)	202.01	2.25 (1.47-3.63)	14.90
Central Latin America	19767.94 (12654.86-30206.64)	18.49	7.56 (4.83-11.58)	-4.35	284386.93 (204761.93-398511.66)	50.08	106.31 (76.67-148.96)	-5.18	567.62 (500.56-641.98)	100.34	0.21 (0.19-0.24)	9.33	68736.03 (50778.77-91954.76)	63.32	25.64 (18.93-34.27)	0.99
Central Europe	2566.80 (1709.02-3684.92)	-27.12	3.08 (2.00-4.57)	4.70	38530.52 (28062.51-51779.20)	-11.05	35.91 (25.34-49.44)	4.34	35.80 (31.54–40.49)	-54.47	0.03 (0.02-0.03)	-54.71	6993.69 (4796.47-10184.69)	-29.67	6.34 (4.17-9.49)	-18.60
Central Asia	1927.36 (1304.81-2758.03)	30.35	2.10 (1.42-2.99)	7.58	21948.63 (15703.48-29275.13)	53.88	22.51 (16.26-30.00)	10.81	103.67 (73.90-122.88)	258.65	0.10 (0.07-0.12)	108.50	8086.28 (6252.91-10171.06)	139.25	8.07 (6.23-10.21)	57.81
Caribbean	3551.88 (2205.89-5402.48)	8.64	7.82 (4.84-11.94)	2.13	51733.83 (37817.54-71489.28)	30.31	106.38 (77.47-147.92)	2.46	100.74 (82.45-119.76)	205.96	0.20 (0.16-0.24)	100.13	11995.12 (8918.45-16349.22)	67.54	24.47 (18.12-33.54)	27.35
Australasia	2861.08 (1957.29-4183.14)	21.90	12.43 (8.34-18.59)	6.10	67122.09 (49214.00-93126.84)	47.42	225.80 (164.31-314.59)	6.98	12.57 (10.87-14.64)	1046.07	0.04 (0.03-0.05)	694.06	9728.07 (6064.43-14948.21)	56.22	32.77 (20.01-51.15)	13.48
Andean Latin America	3573.42 (2248.83-5471.13)	50.51	5.27 (3.32-8.08)	5.02	44834.67 (32060.62-63967.40)	89.81	64.08 (46.11-90.87)	6.26	175.82 (143.60-211.24)	215.47	0.26 (0.21-0.32)	51.02	14885.03 (11528.06-19083.86)	136.90	21.44 (16.63-27.46)	28.03

Table S14. **Incidence, Prevalence, Deaths, DALYs, and their ASRs for Other drug use disorders in 2021 and percentage changes from 1990 to 2021**

	Incidence (95%UI)	Change (%)	ASIR (95%UI)	Change (%)	Prevalence (95%UI)	Change (%)	ASPR (95%UI)	Change (%)	Deaths (95%UI)	Change (%)	ASMR (95%UI)	Change (%)	DALYs (95%UI)	Change (%)	ASDR (95%UI)	Change (%)
Global	6753173.20 (5195841.70-8590638.31)	55.56	81.49 (62.45-103.64)	-5.99	1513973.95 (1235824.47-1848403.90)	63.39	18.17 (14.82-22.12)	-2.61	15287.43 (14298.81-16286.68)	29.36	0.18 (0.17-0.20)	-22	878804.15 (800305.99-971466.00)	23.05	10.69 (9.74-11.80)	-21.29
Middle SDI	2036084.24 (1540073.83-2620924.18)	54.36	74.50 (56.28-95.40)	-10.01	352729.13 (277412.56-444026.13)	40.25	12.82 (10.13-16.20)	-19.23	4426.46 (3678.95-5126.92)	-31.17	0.17 (0.14-0.19)	-59.17	247035.42 (210328.75-283824.82)	-33.03	9.36 (7.99-10.74)	-56.32
High SDI	1553605.28 (1217723.21-1936480.80)	36.46	132.22 (102.22-166.22)	13.08	591427.09 (500544.63-692165.42)	88.35	49.09 (41.37-57.69)	52.03	6704.88 (6165.04-7366.97)	319.26	0.54 (0.50-0.60)	231.01	377878.56 (343216.17-418353.36)	227.68	32.84 (29.88-36.31)	173.3
Low-middle SDI	1275856.94 (965507.05-1639069.56)	124.67	67.09 (51.04-86.16)	10.89	213143.80 (169299.07-266414.81)	131.29	11.32 (9.07-14.14)	13.47	1404.11 (1220.01-1783.81)	210.35	0.09 (0.07-0.11)	55.78	85638.40 (71420.77-104299.97)	164.36	4.55 (3.82-5.54)	36.79
High-middle SDI	1380038.76 (1049481.92-1780210.21)	23.22	91.49 (69.02-117.21)	-9.32	279998.44 (224207.19-350233.57)	16.78	18.38 (14.55-22.89)	-15.27	2433.24 (2230.43-2643.86)	-25.23	0.16 (0.15-0.18)	-45.07	144176.60 (128332.35-160696.40)	-24.32	10.09 (9.05-11.21)	-39.9
Low SDI	502409.30 (373474.96-643697.00)	161.45	56.51 (42.82-71.90)	7.56	75631.18 (59271.89-96353.85)	166.22	8.65 (6.89-10.93)	9.81	312.25 (218.65-584.05)	305.44	0.04 (0.03-0.08)	70.22	23659.42 (17312.25-38003.83)	243.11	2.61 (1.96-4.13)	41.85
Western Sub-Saharan Africa	206507.47 (153016.52-265267.14)	191.91	56.73 (43.19-71.98)	11.84	31264.21 (24142.93-39681.70)	203.82	8.81 (7.03-11.16)	17.54	0.08 (0.05-0.15)	555.23	0.00 (0.00-0.00)	111.20	3447.79 (2066.08-5206.39)	204.27	0.97 (0.59-1.44)	17.72
Western Europe	655562.35 (516233.91-	20.51	146.89 (114.29-	11.64	224210.06 (189249.87-	44.53	49.29 (41.46-57.13)	31.61	1686.36 (1590.09-	144.74	0.36 (0.34-0.38)	119.15	103173.02 (93153.47-	96.99	24.08 (21.83-26.68)	85.96

	819995.15)		184.79)		261206.52)				1787.79)				114623.66)			
Tropical Latin America	176311.20 (132995.60-228103.98)	124.79	68.33 (51.33-88.43)	20.41	28113.66 (21923.36-35994.01)	142.77	10.86 (8.44-13.92)	28.79	259.07 (237.04-283.61)	1207.54	0.11 (0.10-0.12)	656.71	16177.83 (14555.22-18164.76)	583.67	6.65 (5.99-7.45)	308.80
Southern Sub-Saharan Africa	62324.97 (47753.25-78153.48)	108.63	74.81 (57.43-93.96)	7.05	10616.38 (8411.09-13374.16)	113.45	12.85 (10.24-16.05)	8.53	167.46 (146.54-190.32)	82.43	0.23 (0.20-0.27)	6.65	9042.14 (7940.35-10280.5)	66.01	11.18 (9.83-12.74)	-2.91
Southern Latin America	69072.09 (50694.10-89693.63)	54.62	93.48 (68.39-121.15)	0.03	13062.57 (9927.49-17395.39)	54.38	17.59 (13.28-23.51)	-1.07	17.28 (14.34-20.18)	159.57	0.02 (0.02-0.03)	51.24	2058.85 (1460.9-2848.96)	70.55	2.77 (1.96-3.84)	9.70
Southeast Asia	485720.94 (363293.94-627683.16)	94.94	62.94 (46.97-81.27)	4.53	79327.35 (62163.44-101988.31)	101.88	10.24 (8.04-13.11)	6.41	253.68 (173.44-347.02)	143.47	0.04 (0.03-0.06)	17.04	18256.55 (13272.78-23776.26)	104.03	2.49 (1.81-3.22)	12.63
South Asia	1254965.67 (947460.59-1618087.59)	141.61	65.94 (49.97-84.78)	15.43	205833.73 (163791.33-257715.69)	152.63	10.92 (8.67-13.69)	20.02	1370.53 (1149.46-1725.96)	241.19	0.09 (0.07-0.11)	56.08	76944.37 (63757.32-95544.63)	180.76	4.19 (3.49-5.16)	38.65
Oceania	9304.63 (7035.54-11855.92)	139.75	71.01 (54.14-90.31)	-0.01	1519.41 (1196.31-1928.98)	141.90	11.67 (9.17-14.79)	0.09	1.77 (1.13-3.17)	71.45	0.02 (0.01-0.03)	-25.30	257.93 (174.16-375.63)	107.68	1.98 (1.36-2.89)	-10.55
North Africa and Middle East	514580.49 (385279.13-673505.39)	162.03	76.60 (57.60-99.30)	5.26	91016.35 (70486.42-116465.79)	175.82	13.53 (10.54-17.30)	9.13	1765.03 (1472.3-2128.19)	175.02	0.28 (0.24-0.34)	21.08	100912.59 (85524.16-121684.86)	158.56	15.27 (12.94-18.46)	16.81
High-income North America	599916.99 (472870.88-761638.71)	54.61	158.67 (122.50-205.36)	28.49	297541.94 (250531.59-353769.25)	153.17	76.38 (64.09-90.25)	102.45	4096.57 (3628.99-4784.07)	545.20	1.02 (0.91-1.19)	408.82	226352.07 (200183.50-259663.52)	401.20	59.67 (52.73-68.53)	314.20
High-income	174199.51	-2.01	89.22	-2.85	33795.05	-1.97	16.87 (12.49-	-4.39	64.43 (57.63-	58.68	0.02 (0.02-	4.44	5746.92	1.46	2.85 (2.06-	-2.91

Asia Pacific	(129849.28-227804.76)		(64.91-117.30)		(25018.89-44225.19)		22.85)		70.37)		0.02)		(4189.25-7984.36)		3.91)	
Eastern Sub-Saharan Africa	163723.58 (121268.45-212430.68)	167.03	48.51 (36.55-62.36)	5.27	23998.48 (18491.23-30664.92)	175.40	7.20 (5.67-9.18)	8.36	21.33 (13.14-44.53)	337.69	0.01 (0.01-0.02)	59.03	3616.28 (2344.68-5447.61)	207.84	1.11 (0.72-1.66)	21.20
Eastern Europe	269137.71 (203148.13-340992.32)	-1.29	115.92 (87.05-146.22)	4.08	68339.71 (55483.58-83572.80)	3.48	29.41 (23.88-35.65)	9.12	1164.63 (1057.35-1282.9)	46.57	0.51 (0.46-0.56)	55.47	63648.51 (57532.39-70106.59)	42.69	29.60 (26.91-32.45)	58.03
East Asia	1504423.92 (1140049.15-1925289.55)	12.51	88.03 (66.19-112.56)	-17.46	274586.61 (217404.80-344605.99)	-1.53	15.84 (12.50-20.06)	-29.12	3031.89 (2290.43-3783.99)	-61.94	0.18 (0.14-0.22)	-72.44	170396.33 (134389.71-207487.73)	-62.06	10.66 (8.40-12.98)	-68.48
Central Sub-Saharan Africa	66098.93 (49484.12-85654.68)	177.59	60.73 (46.06-77.73)	1.95	10352.62 (8256.51-13035.64)	180.15	9.69 (7.75-12.09)	2.81	4.36 (2.09-9.8)	310.56	0.00 (0.00-0.01)	49.20	1364.91 (819.13-2027.13)	198.80	1.26 (0.76-1.87)	9.34
Central Latin America	194420.82 (145771.64-250918.65)	92.71	72.15 (54.14-93.22)	-3.27	32799.63 (25541.15-41984.45)	85.89	12.15 (9.46-15.57)	-8.57	219.48 (193.94-247.06)	153.87	0.08 (0.07-0.09)	45.56	15365.86 (13361.55-17759.70)	118.83	5.71 (4.97-6.60)	27.65
Central Europe	110262.80 (83113.58-143124.93)	3.72	86.12 (63.91-111.03)	7.48	21506.56 (17055.88-27429.10)	10.43	16.52 (13.06-21.12)	12.83	108.97 (98.56-118.83)	-3.83	0.09 (0.08-0.09)	-2.71	7242.98 (6169.15-8517.31)	-7.59	6.22 (5.37-7.19)	1.79
Central Asia	82325.07 (61067.32-105555.07)	69.18	80.53 (60.00-102.81)	1.99	15398.59 (12226.37-19277.81)	73.28	15.04 (11.97-18.87)	3.14	110.8 (90.99-130.73)	199.02	0.12 (0.10-0.14)	84.21	7049.94 (5829.49-8355.00)	154.28	7.02 (5.82-8.32)	59.02
Caribbean	37327.82 (28486.20-47680.85)	48.50	74.74 (56.94-95.65)	-3.60	7193.99 (5813.57-9033.59)	42.64	14.34 (11.57-17.95)	-9.44	36.97 (31.39-44.11)	261.55	0.07 (0.06-0.09)	120.05	2407.60 (1998.12-2905.92)	133.90	4.83 (4.01-5.83)	54.70

Australasia	64924.03 (51473.27-80495.02)	62.47	197.85 (155.75-246.44)	9.79	34591.89 (29304.52-39677.01)	98.29	102.72 (87.20-118.37)	29.75	840.17 (749.3-940.8)	505.75	2.36 (2.11-2.64)	273.99	41159.67 (36652.09-45609.13)	356.04	123.67 (109.73-137.69)	199.35
Andean Latin America	52062.21 (38515.53-67301.35)	125.06	75.53 (55.90-97.52)	3.14	8905.18 (6861.42-11499.32)	126.74	12.96 (10.02-16.73)	3.39	66.57 (52.43-85.98)	98.26	0.10 (0.08-0.13)	0.03	4181.99 (3315.47-5282.40)	80.34	6.07 (4.81-7.66)	-4.22

Table S15. Global projections of incidence, prevalence, deaths, DALYs, and ASRs of drug use disorders from 2022 to 2036 (ARIMA model)

year	Absolute numbers (95%CI)				Age-standardized rate (per 100,000 population, 95%CI)			
	Incidence	Prevalence	Deaths	DALYs	ASIR	ASPR	ASMR	ASDR
2022	13696753.02 (13625382.46-13768123.59)	53182957.21 (52927309.37-53438605.06)	141913.73 (138964.23-144863.23)	15736655.45 (15547321.54-15925989.37)	171.09 (169.63-172.55)	660.95 (657.37-664.54)	1.68 (1.64-1.73)	191.67 (188.95-194.38)
2023	13784143.67 (13660343.87-13907943.47)	53181080.49 (52372651.02-53989509.96)	146549.53 (139954.25-153144.81)	15881462.71 (15426075.71-16336849.72)	174.51 (169.91-179.12)	657.77 (646.44-669.11)	1.72 (1.62-1.82)	192.06 (185.57-198.56)
2024	13871534.31 (13691333.59-14051735.04)	53110306.21 (51376418.17-54844194.25)	151185.33 (140149.32-162221.35)	15996583.31 (15182761.81-16810404.81)	179.66 (169.79-189.53)	654.27 (629.95-678.59)	1.75 (1.59-1.92)	192.16 (180.60-203.72)
2025	13958924.96 (13717458.82-14200391.10)	52970634.36 (49881629.70-56059639.01)	155821.14 (139666.07-171976.21)	16082017.25 (14815610.48-17348424.01)	186.53 (168.94-204.12)	650.44 (607.11-693.77)	1.79 (1.55-2.03)	191.95 (174.02-209.88)
2026	14046315.61 (13738741.92-14353889.30)	52762064.95 (47837939.72-57686190.19)	160456.94 (138582.88-182331.01)	16137764.52 (14321732.71-17953796.33)	195.13 (167.09-223.17)	646.28 (577.21-715.35)	1.83 (1.51-2.15)	191.44 (165.80-217.08)
2027	14133706.25	52484597.98 (45199755.77-	165092.74 (136956.32-	16163825.13 (13697741.05-	205.44 (163.96-	641.80 (539.61-	1.86 (1.45-2.28)	190.63 (155.91-

	(13755376.07-14512036.44)	59769440.19)	193229.17)	18629909.20)	246.93)	743.98)		225.35)
2028	14221096.90 (13767580.89-14674612.91)	52138233.45 (41925110.07-62351356.83)	169728.55 (134829.61-204627.48)	16160199.07 (12940042.58-19380355.56)	217.49 (159.33-275.64)	636.98 (493.73-780.24)	1.90 (1.39-2.41)	189.52 (144.29-234.74)
2029	14308487.54 (13775566.25-14841008.84)	51722971.35 (37974915.78-65471026.93)	174364.35 (132237.09-216491.61)	16126886.35 (12044977.27-20208897.86)	231.25 (152.97-309.54)	631.85 (439.01-824.69)	1.94 (1.32-2.55)	188.10 (130.90-245.30)
2030	14395878.19 (13779523.48-15012223.90)	51238811.70 (33312446.53-69165176.87)	179000.15 (129206.89-228793.42)	16063886.97 (11008887.03-21118886.91)	246.74 (144.67-348.82)	626.38 (374.93-877.83)	1.97 (1.24-2.70)	186.38 (115.69-257.08)
2031	14483268.84 (13779624.58-15186913.10)	50685754.48 (27902955.61-73468553.36)	183635.96 (125762.62-241509.30)	15971200.92 (9828151.23-22114250.61)	263.96 (134.23-393.69)	620.59 (301.02-940.16)	2.01 (1.16-2.86)	184.36 (98.61-270.12)
2032	14570659.48 (13776023.81-15365295.15)	50063799.70 (21713387.40-78414212.00)	188271.76 (121924.44-254619.09)	15848828.21 (8499204.96-23198451.47)	282.89 (121.46-444.33)	614.47 (216.81-1012.13)	2.04 (1.07-3.02)	182.04 (79.62-284.46)
2033	14658050.13 (13768859.85-15547420.42)	49372947.36 (14712152.68-84033742.04)	192907.56 (117709.82-268105.31)	15696768.84 (7018548.05-24374982.65)	303.56 (106.19-500.92)	608.02 (121.85-1094.20)	2.08 (0.98-3.18)	179.42 (58.67-300.17)
2034	14745440.77 (13758257.83-15732623.71)	48613197.46 (6868949.52-90357445.39)	197543.37 (113134.10-281952.63)	15515022.80 (5382748.88-25647296.73)	325.94 (88.24-563.64)	601.25 (15.72-1186.78)	2.12 (0.88-3.35)	176.49 (35.71-317.27)
2035	14832831.42 (13744331.23-15921331.61)	47784549.99 (-1845382.05-97414482.03)	202179.17 (108210.91-296147.43)	15303590.11 (3588445.25-27018734.96)	350.05 (67.45-632.65)	594.15 (-101.99-1290.29)	2.15 (0.77-3.53)	173.27 (10.71-335.82)

2036	14920222.07 (13727183.40-16113260.73)	46887004.96 (-11458980.04-105232990.00)	206814.97 (102952.42-310677.53)	15062470.74 (1632343.62-28492597.86)	375.88 (43.65-708.12)	586.73 (-231.67-1405.13)	2.19 (0.66-3.71)	169.74 (-16.38-355.85)

Table S16. Global projections of incidence, prevalence, deaths, DALYs, and ASRs of drug use disorders from 2022 to 2036 (BAPC model)

Years	Absolute numbers (95%CI)				Age-standardized rate (per 100,000 population, 95%CI)			
	Incidence	Prevalence	Deaths	DALYs	ASIR	ASPR	ASMR	ASDR
2022	13856986.07 (13723102.51-13990869.64)	53809090.24 (52819962.2-54798218.28)	142247.39 (138275.19-146219.59)	15715261.56 (15319710.22-16110812.89)	168.46 (166.05-170.86)	657.13 (641.25-673)	1.65 (1.58-1.73)	187.94 (181.49-194.38)
2023	13926378.76 (13772262.42-14080495.11)	54055274.54 (52972436.09-55138113)	146446.43 (141653.18-151239.67)	15973429.65 (15536394.48-16410464.81)	167.9 (164.32-171.49)	655.41 (634.22-676.59)	1.68 (1.55-1.8)	189.32 (180.46-198.18)
2024	13986433.68 (13800329.24-14172538.12)	54271409.32 (53039093.2-55503725.44)	150774.31 (144685.42-156863.2)	16228673.32 (15726571.91-16730774.74)	167.25 (162.13-172.37)	653.33 (624.97-681.69)	1.7 (1.52-1.88)	190.64 (178.52-202.76)
2025	14037485.38 (13808767.91-14266202.85)	54459481.63 (53022870.27-55896092.99)	155220.75 (147390.32-163051.17)	16481641.13 (15890564.38-17072717.89)	166.51 (159.61-173.42)	650.88 (613.97-687.78)	1.72 (1.47-1.98)	191.9 (175.86-207.94)
2026	14082956.37 (13802454.85-14363457.89)	54626765.89 (52936193.33-56317338.46)	159720.28 (149743.78-169696.78)	16731440.91 (16029051.18-17433830.63)	165.73 (156.84-174.62)	648.1 (601.58-694.63)	1.75 (1.41-2.08)	193.06 (172.55-213.56)
2027	14123431.4 (13783256.02-14463606.78)	54777903.9 (52790065.93-56765741.86)	164276.83 (151774.35-176779.32)	16979028.71 (16145015.4-17813042.01)	164.9 (153.84-175.96)	645.02 (587.98-702.05)	1.77 (1.35-2.19)	194.1 (168.66-219.54)
2028	14156091.29 (13749819.36-14562363.23)	54907565.05 (52586423.81-57228706.28)	169024.95 (153621.86-184428.05)	17225551.79 (16241853.95-18209249.63)	164.02 (150.65-177.38)	641.57 (573.29-709.85)	1.79 (1.27-2.3)	195.05 (164.25-225.84)
2029	14180775.46 (13702962.69-14658588.24)	55009733.2 (52324739.3-57694727.11)	174059.62 (155366.49-192752.76)	17470637.38 (16320789.4-18620485.35)	163.06 (147.26-178.87)	637.75 (557.61-717.89)	1.8 (1.18-2.43)	195.9 (159.36-232.43)
2030	14197716.9 (13643532.95-14751900.85)	55084229.73 (52008357.16-58160102.31)	179377.74 (156982.76-201772.71)	17714699.43 (16382983.79-19046415.07)	162.04 (143.69-180.4)	633.58 (541.05-726.1)	1.82 (1.09-2.56)	196.65 (154.03-239.28)
2031	14209974.94 (13574821.32-	55137115.86 (51645092.55-	184909.15 (158380.24-	17957381.38 (16428324.41-	161 (139.99-	629.15 (523.75-	1.84 (0.98-	197.31 (148.25-

	14845128.56)	58629139.16)	211438.07)	19486438.36)	182.01)	734.55)	2.7)	246.36)
2032	14219194.98 (13498563.05-14939826.91)	55176421.28 (51244227.02-59108615.55)	190660.28 (159529.82-221790.75)	18199995.33 (16458162.27-19941828.39)	159.93 (136.17-183.69)	624.52 (505.8-743.24)	1.86 (0.87-2.84)	197.86 (142.06-253.65)
2033	14217465.18 (13408028.23-15026902.12)	55177259.27 (50785453.83-59569064.71)	196777.93 (160513.27-233042.59)	18440506.84 (16471036.24-20409977.43)	158.82 (132.22-185.42)	619.65 (487.23-752.07)	1.87 (0.75-2.99)	198.3 (135.47-261.13)
2034	14207737.01 (13306340.19-15109133.83)	55154844.19 (50284878.66-60024809.72)	203400.49 (161382.02-245418.97)	18681856.18 (16469843.85-20893868.52)	157.66 (128.15-187.16)	614.53 (468.09-760.96)	1.88 (0.63-3.14)	198.65 (128.52-268.79)
2035	14190932.31 (13194621.07-15187243.55)	55110236.18 (49745054.72-60475417.64)	210556.52 (162073.49-259039.55)	18924681.97 (16454804.58-21394559.36)	156.44 (123.97-188.91)	609.19 (448.47-769.9)	1.9 (0.49-3.3)	198.92 (121.22-276.61)
2036	14170302.17 (13076016.51-15264587.83)	55048416.8 (49171531.51-60925302.09)	218197.36 (162453-273941.73)	19169001.14 (16425188.91-21912813.37)	155.2 (119.7-190.7)	603.71 (428.45-778.98)	1.91 (0.35-3.46)	199.09 (113.59-284.59)
